# A eutherian-specific microRNA controls the translation of Satb2 in a model of cortical differentiation

**DOI:** 10.1016/j.stemcr.2021.04.020

**Published:** 2021-05-20

**Authors:** Manuella Martins, Silvia Galfrè, Marco Terrigno, Luca Pandolfini, Irene Appolloni, Keagan Dunville, Andrea Marranci, Milena Rizzo, Alberto Mercatanti, Laura Poliseno, Francesco Morandin, Marco Pietrosanto, Manuela Helmer-Citterich, Paolo Malatesta, Robert Vignali, Federico Cremisi

**Affiliations:** 1 Scuola Normale, Pisa, Italy; 2 Istituto di Biofisica CNR, Pisa, Italy; 3 Dipartimento di Biologia, Università Roma Tor Vergata, Roma, Italy; 4 Dipartimento di Medicina Sperimentale, Università di Genova, Genova, Italy; 5 Ospedale Policlinico San Martino, IRCCS per l'Oncologia, Genova, Italy; 6 Istituto di Fisiologia Clinica CNR, Pisa, Italy; 7 Oncogenomics Unit, Core Research Laboratory, ISPRO, Pisa, Italy; 8 Dipartimento di Scienze Matematiche, Fisiche e Informatiche, Università di Parma, Parma, Italy; 9 Dipartimento di Biologia, Università di Pisa, Pisa, Italy

**Keywords:** cortex, microRNA, cortical layering, cell fate, developmental timing, post-transcriptional control, mammalian evolution, cell identity, *in vitro* corticogenesis, miR-catch, neural stem cells, corpus callosum, SATB2

## Abstract

Cerebral cortical development is controlled by key transcription factors that specify the neuronal identities in the different layers. The mechanisms controlling their expression in distinct cells are only partially known. We investigated the expression and stability of *Tbr1*, *Bcl11b*, *Fezf2*, *Satb2*, and *Cux1* mRNAs in single developing mouse cortical cells. We observe that *Satb2* mRNA appears much earlier than its protein and in a set of cells broader than expected, suggesting an initial inhibition of its translation, subsequently released during development. Mechanistically, *Satb2* 3′UTR modulates protein translation of GFP reporters during mouse corticogenesis. We select miR-541, a eutherian-specific miRNA, and miR-92a/b as the best candidates responsible for SATB2 inhibition, being strongly expressed in early and reduced in late progenitor cells. Their inactivation triggers robust and premature SATB2 translation in both mouse and human cortical cells. Our findings indicate RNA interference as a major mechanism in timing cortical cell identities.

## Highlights

• mRNAs for key transcription factors are differentially stable during corticogenesis
• miRNAs show a dynamic profile of expression in a model of mouse corticogenesis
• miR-541 and miR-92a/b bind *Satb2* 3′UTR and prevent SATB2 translation
• Antagonizing mir-541 and miR-92a/b anticipates SATB2 protein production

## Graphical abstract

**Figure:**
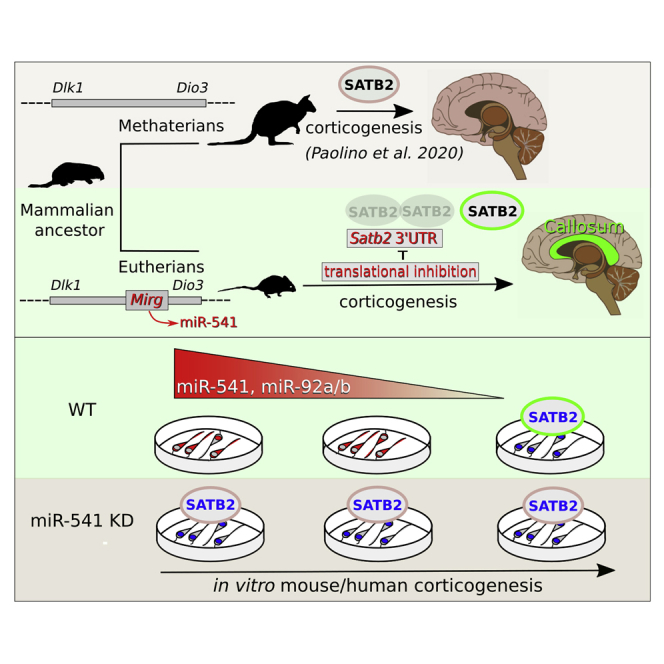

## Teaser

In this article, Cremisi and colleagues show that post-transcriptional mechanisms are involved in controlling key functional aspects of SATB2-expressing cortical neurons. They show that mir-541, a eutherian-specific miRNA, delays SATB2 protein production in an *in vitro* model of cortical cell differentiation. These results may explain the heterochronic shift of SATB2 appearance in the eutherian compared with the metatherian cortex.

## Introduction

**Figure 1:**
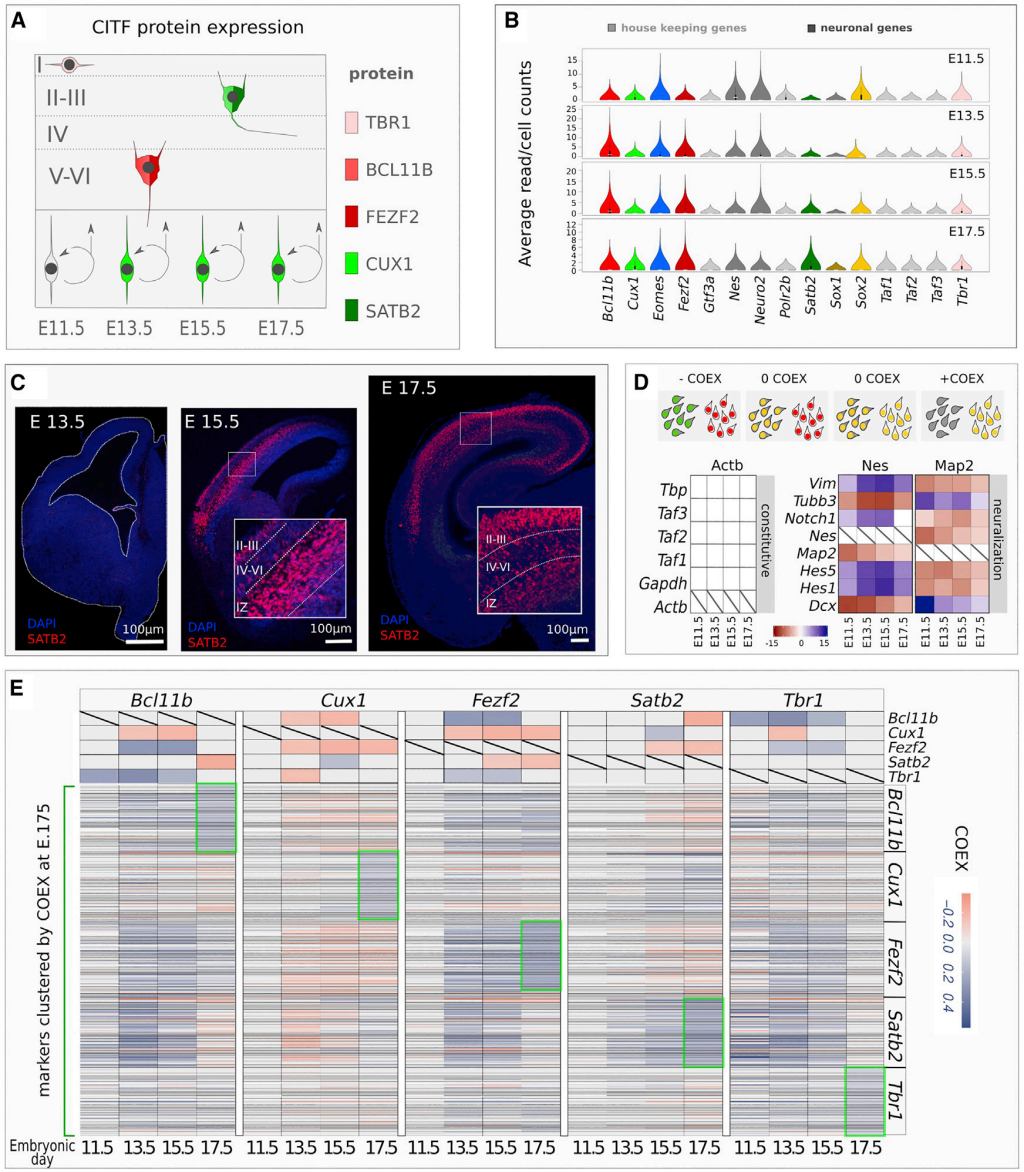
CITF expression analysis (A) Simplified outline of cortical layering. Layers are labeled by Roman numerals. (B) Violin plots show average raw counts/cell of indicated genes. Constitutive genes are in light gray. (C) Coronal sections of mouse embryonic brain showing SATB2 immunodetection at different embryonic (E) developmental times of corticogenesis. Roman numerals indicate cortical layers. IZ, intermediate zone. (D) Top schematic shows COTAN COEX relation to the pattern of expression of two genes (red and green) in single cell. Bottom shows COEX values of pairs of constitutive genes (left matrix) or neural differentiation markers (right matrix) at the different developmental times shown in labels. (E) COTAN COEX values of CITFs and of their most closely associated genes at E17.5. The top side of the matrix shows the COTAN COEX relation between pairs of CITFs. The bottom part of the matrix reports COTAN COEX values between distinct CITFs and the genes that are more highly co-expressed with each of them at E17.5 (green boxes).

The mammalian neocortex consists of six cell layers (I–VI) generated by radial migration of neuroblasts following an inside-out mechanism (Greig et al., 2013). Glutamatergic projection neurons are formed after the generation of layer I neurons in two main neurogenetic waves: deep projection neurons (DPNs) of layers V–VI are generated first, followed by superficial projection neurons (SPNs) of the supra-granular layers II–III (Figure 1A). Generation of layer IV neurons follows the generation of DPNs and precedes SPNs formation. Proper regulation of this developmental process is crucial and its impairment results in various disorders such as brain malformations or psychiatric diseases (Sun and Hevner, 2014). The capability to generate distinct classes of neurons depends on the progenitor cell (PC) cycle state and neuron birth date (McConnell and Kaznowski, 1991). Epigenetic birthmarks may regulate the ability of PCs to establish neuron identity in the first hour following the last cell division (Telley et al., 2019). After this, the expression of a few cell identity transcription factors (CITFs) is necessary to impart distinct cell fates, with TBR1, BCL11B, FEZF2, SATB2, and CUX1 playing an important role among them (Alcamo et al., 2008; Cubelos et al., 2010; Hevner et al, 2001, 2003; Leone et al., 2015; Srinivasan et al., 2012). These factors may initially establish early mutual activating or repressive interactions; beyond this early phase, depending on the cell context and the timing of corticogenesis, some of these interactions may change and combinatorial action may ensue to refine terminal cell phenotype (Alcamo et al., 2008; Britanova et al., 2008; Chen et al., 2008; Harb et al., 2016; Jaitner et al., 2016; McKenna et al., 2015). A precise timing of expression of these and other factors is required to ensure appropriate differentiation of the neocortex. The exact mechanisms dictating the timely expression of CITFs in one given PC and its progeny is still under scrutiny.

The evolution of the mammalian cortex is characterized by the progressive thickening of the supra-granular cell layer(s) (Dehay et al., 2015; Dehay and Kennedy, 2007). A sudden evolutionary change during mammalian cortex evolution may be the heterochronic appearance of the cortical transcription factor SATB2 with respect to the corresponding mRNA. Indeed, it was recently shown that SATB2 protein expression is delayed in eutherians compared with metatherians, and such delay seems responsible for the development of the inter-hemispheric callosal connections generated from the supra-granular cells in eutherians (Paolino et al., 2020). After its evolutionary appearance, the continuous expansion of the corpus callosum (CC), and of the supra-granular cell layer it stems from, represents the distinguishing feature of the placental neocortex, including that of higher primates. Notably, in higher primates, SATB2 protein appearance is delayed over an extended period, possibly crucial for supra-granular cell layer expansion (Otani et al., 2016). In this aspect, the control of developmental timing of SATB2 during cortical neurogenesis may be of crucial importance. In this paper, we have first investigated the differential stability of mRNAs for key CITFs involved in mammalian corticogenesis, namely *Bcl11b*, *Cux1*, *Tbr1*, *Fezf2*, and *Satb2*, by exon/intron (E/I) stability analysis (EISA) (Gaidatzis et al., 2015). We find that among them only *Satb2* mRNA shows an increase in E/I ratio due to an improved stability and rate of its transcription. We then show that a post-transcriptional control is played by microRNAs (miRNAs) acting on *Satb2* 3′UTR. We isolated miRNAs that bind to this region and focus on miR-541, a new, eutherian-specific miRNA; we show that miR-541 delays, both *in vivo* and *in vitro*, SATB2 protein production with respect to *Satb2* mRNA transcription. We discuss the potential implications of miR-541 action in the scenario of cortical evolution.

## Results

### *Satb2* is co-transcribed with other CITFs in early cortical cells before its translation

Since DPNs and SPNs are sequentially generated in an inside-out fashion from embryonic day 11.5 (E11.5) to E17.5 in mouse (Figure 1A), we expect that the mRNA of CITFs is regulated in selected PCs in this time window. We tested this assumption by re-analyzing single-cell RNA sequencing (scRNA-seq) datasets of mouse cortex at E11.5, E13.5, E15.5, and E17.5, generated by droplet sequencing from dissociated whole embryonic cortices (average depth more than 50,000 reads/cell; transcriptomes from 2,000 cells at E11.5, E13.5, and E17.5; 5,000 cells at E15.5) (Yuzwa et al., 2017). We compared the average expression levels of the 5 CITFs (raw counts/cell) with those of constitutively expressed transcription factors (Figure 1B). The mRNA expression levels of all 5 CITFs are comparable with those of constitutive transcription factors since E11.5, indicating that these five mRNAs could have a biological relevance since very early stages of corticogenesis. However, we did not detect SATB2 translation before E15.5 (Figure 1C), although a dynamic pattern of *Satb2* transcriptional activation in the dorsal telencephalon starts from E11.5 (Tashiro et al., 2011). Although a minority of SATB2-positive cells were reported at E13.5 (Alcamo et al., 2008; Britanova et al., 2008), a reliable onset of SATB2 protein expression was not described earlier than E14 (Paolino et al., 2020), suggesting a post-transcriptional regulation of *Satb2* mRNA.

To get insights into the pattern of CITF transcriptional activation in specific cell subsets, we analyzed CITF co-expression in single cells by co-expression table analysis (COTAN) (Galfrè et al., 2020). COTAN can assess the co-expression of gene pairs in a cell and, by extending this analysis to all gene pairs in the whole transcriptome, can indicate the tendency of a gene to be constitutively expressed or expressed in a subset of differentiating/differentiated cells. Positive co-expression index (COEX) denotes co-expression of two genes, while negative COEX indicates disjoint expression; COEX near zero is expected if one or both are constitutive genes (Figure 1D, top) or when the statistical power is too low. Accordingly, our analysis gives COEX values close to zero for constitutive mRNA pairs (Figure 1D, left; Data S1). Conversely, high co-expression (positive COEX) is found for mRNA pairs of known molecular markers of neural PCs (*Nestin*, *Vimentin*, *Notch1*, *Hes1-5*) or post-mitotic cells and differentiating neurons (*Dcx*, *Tubb3*, *Map2*). Finally, negative COEX (disjoint expression) is detected between mRNA pairs of these two groups at all developmental stages (Figure 1D). All CITFs show reciprocal mRNA co-expression patterns consistent with their known protein expression pattern in different cell types, except *Satb2*, whose COEX with each of the other four CITFs at E11.5 and E13.5 is comparable with that of constitutive genes (compare Figures 1A and 1E, top).

We considered the genes most highly co-expressed with each CITF gene at E17.5 (Figure 1E bottom, Figure S1). At this stage, the final pattern of co-expression of each CITF gene with co-clustered markers (green boxes in Figure 1E) differs from the patterns at earlier stages (Figure 1E). This suggests that initial CITF gene expression is not cell layer specific, but cell-specific CITF gene expression is reached toward the end of layer formation.

**Figure 2:**
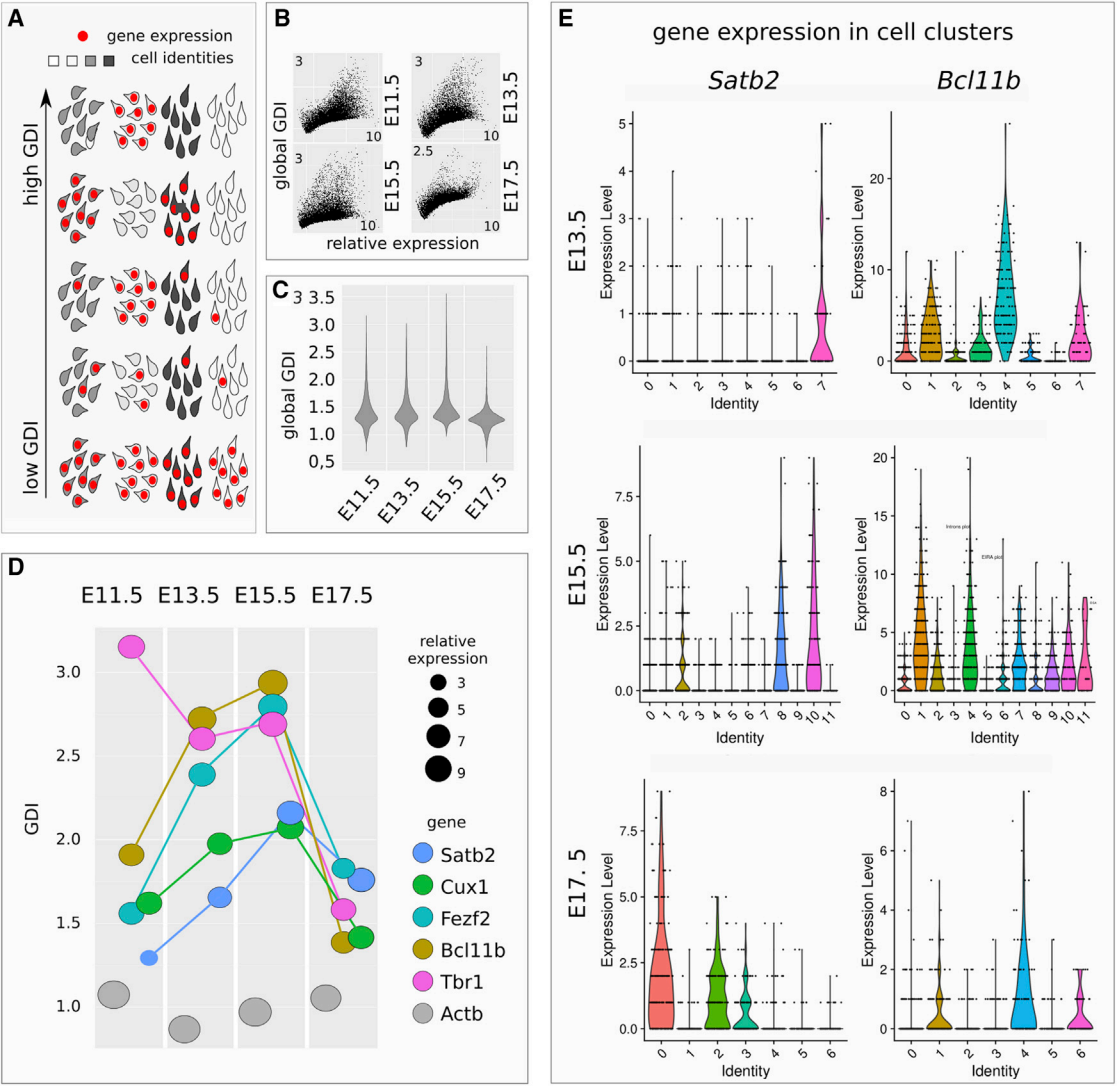
CITF transcription in distinct cell clusters (A) Schematic shows how GDI can indicate the degree of gene pair co-expression in cell populations with different cell identities. (B) Plots show GDI and gene mRNA expression levels at different developmental times. (C) Violin plots report global GDI distribution during corticogenesis. (D) Distinct CITFs show different GDI according to their translational onset. (E) Clustering of DIV13.5, DIV15.5, and DIV17.5; violin plots show count distribution for the indicated gene in cell clusters. Analysis was performed by R package Seurat 4.0.

COTAN Gene Differentiation Index (GDI) discerns between constitutive and non-constitutive genes by globally integrating COEX values (Galfrè et al., 2020) (Figure 2A). We used GDI analysis to infer the propensity of CITFs to be expressed in restricted cell subsets during corticogenesis. Notably, the global relation between GDI and mRNA levels (Figure 2B), and the global GDI distribution (Figure 2C), are comparable in the four analyzed stages. This observation supports the use of GDI analysis to evaluate whether an mRNA species changes its pattern of cell distribution during corticogenesis, and becomes restricted to a particular cell lineage/layer. Unlike constitutive genes such as *Actb*, CITFs showed marked GDI changes during corticogenesis (Figure 2D). *Tbr1* mRNA shows a peak at E11.5, consistent with early localized TBR1 protein expression in layer 1 neurons (Hevner et al., 2001). *Bcl11b* and *Fezf2*, followed by *Satb2* and *Cux1*, increase their GDIs until E15.5, paralleling their respective onset of protein expression (compare Figures 2D with 1A).

The drop of GDI observed at E17.5 correlates with, and might be explained by, the increased heterogeneity of the cell types co-expressing different combinations of CITF proteins at the end of corticogenesis (Lodato and Arlotta, 2015), although it may also be due to post-transcriptional CITF regulation. Notably, *Satb2* displays the lowest GDI levels among CITFs at E11.5–13.5, when its protein is not yet detectable, suggesting that post-transcriptional control accounts, at least in part, for the subsequent restricted expression of SATB2 protein in SPNs. Finally, we used a conventional scRNA-seq data clustering (Figure 2E). The lack of a cell-type restricted distribution of *Satb2* mRNA at early stages is also suggested by its partial overlap with *Bcl11b* mRNA in E13.5 and E15.5 cell clusters, compared with E17.5 clusters.

### *Satb2* 3′UTR drives RNA-induced silencing complex-dependent translational inhibition in early cortical cells

**Figure 3:**
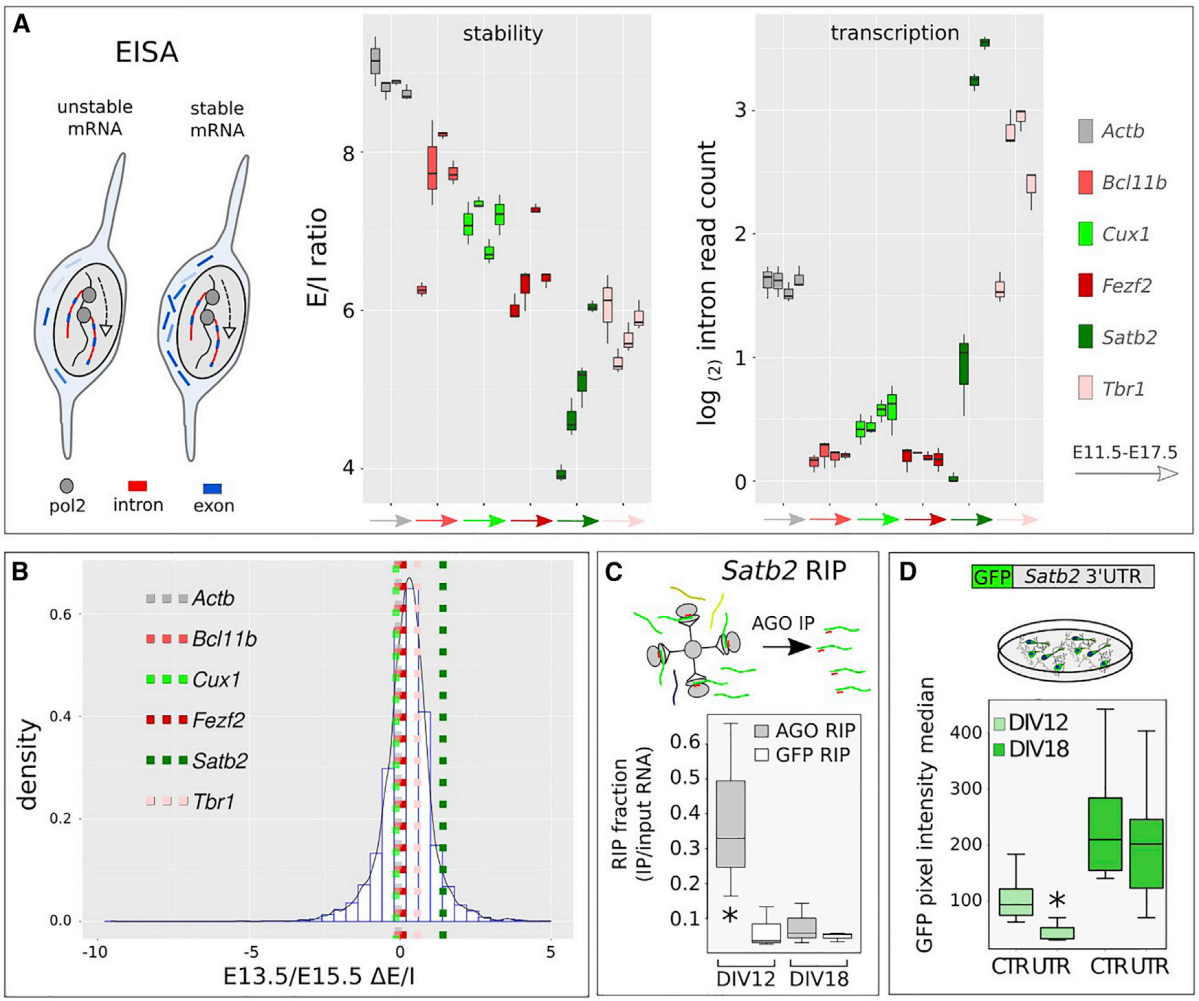
Cortical mRNA E/I analysis and *Satb2* translational inhibition (A) EISA of CITF mRNAs. Outline shows different ratios of exonic and intronic sequences in relation to mRNA stability as rationale at the basis of EISA. Box plots show the ratio of E/I read counts, and intron read counts, for distinct CITFs and *Actb* (constitutive control gene) in cortical progenitors at different *in vivo* embryonic times. (B) Density plot of E/I ratio fold change between E13.5 and E15.5. (C) qRT-PCR evaluation of Argonaute (AGO)-interacting *Satb2* mRNA. Values on y axis report the ratio of RT-PCR-detected, immunoprecipitated *Satb2* mRNA with respect to the input (AGO RIP). GFP RIP, control immunoprecipitation with anti-GFP Ab. N = 3 independent experiments. Asterisk indicates p-value = 0.049 (Student’s t test). (D) Expression of *Satb2* 3′ UTR-bearing GFP reporter after lipofection in corticalized mESCs. N = 3 independent experiments. Cells were transfected 48 h before the time of analysis indicated in labels. Asterisk indicates p-value = 0.000061 (Wilcoxon signed rank test).

We then took advantage of EISA (Gaidatzis et al., 2015; La Manno et al., 2018) to verify whether a time-dependent instability of *Satb2* mRNA could account for the inability to detect SATB2 protein at E13.5, when *Satb2* transcription is already robust and coincident with that of *Bcl11b*. EISA evaluates changes of stability of specific mRNAs during developmental processes, assuming that the intronic sequences are rapidly spliced and that their levels reflect the gene transcriptional rate (see schematic in Figure 3A, left). Because layer identity is assigned before neuron birth date (McConnell and Kaznowski, 1991; Telley et al., 2019), we analyzed RNA-seq datasets of PCs (Chui et al., 2020). We observed that *Satb2* E/I ratio significantly increases from E11.5 to E17.5, *Bcl11b* E/I increases from E11.5 to E13.5, and *Fezf2* E/I increases from E13.5 to E15.5, while the E/Is of the other CITFs and of *Actb* show no significant changes (Figure 3A, middle panel). Notably, *Satb2* E/I increase is paralleled by a dramatic increase of its transcription levels from E11.5 to E17.5 (Figure 3A, right), as measured by intron read abundance, making its E/I increase more relevant than that of *Bcl11b* and *Fezf2*. *Satb2* E/I fold change between E13.5 and E15.5 settles in the highest quartile of the E/I increase (Figure 3B and Data S2), suggesting high biological relevance and supporting a close relationship between the increase of *Satb2* mRNA stability and the onset of SATB2 translation. We thus focused our attention on *Satb2* post-transcriptional regulation.

We reasoned that changes in *Satb2* mRNA stability could be induced by miRNAs. Indeed, by high-throughput analysis of miRNA-mRNA interactions at single-cell level, distinct miRNAs were recently associated to functional modules involved in the control of cortical cell identities (Nowakowski et al., 2018). To gain insights on RNA interference in early corticogenesis, we employed mouse embryonic stem cells (mESCs), whose *in vitro* neural differentiation can closely reproduce the early stages of cortical development, including time-regulated expression of TBR1, BCL11B, and SATB2 protein (Bertacchi et al., 2015; Gaspard et al., 2008). In these corticalized mESCs, we measured the enrichment of *Satb2* mRNA after AGO2 immunoprecipitation. By qRT-PCR, a significant enrichment of AGO2-bound *Satb2* mRNA is detected after 12 days *in vitro* (DIV) compared with control anti-GFP immunoprecipitation, indicating a strong miRNA silencing activity in early *in vitro* corticogenesis (Figure 3C). Notably, we found no enrichment at DIV18, consistent with a significant increase of SATB2-positive cells at this time (Bertacchi et al., 2015).

The changing ability of *Satb2* mRNA to bind AGO2 during development is in line with the ability of its 3′UTR to inhibit protein translation in early, but not late, cortical cells. Indeed, at DIV12 the transfection of a GFP reporter carrying *Satb2* 3′UTR yields decreased fluorescence levels compared with control, while at DIV18 the reporter activity is not significantly affected (Figure 3D), consistent with robust SATB2 translation at this late stage (Bertacchi et al., 2015). *Satb2* 3′UTR is able to control translation also *in vivo*, as shown by *in utero* electroporation (IUE) of a GFP reporter/sponge. At stage E13.5, the ratio of SATB2-GFP double-positive cells to GFP-positive cells is significantly higher in a cortex electroporated with a 3′UTR-bearing sensor compared with a control cortex (Figure S2). These results show that *Satb2* 3′UTR can inhibit the translation of its mRNA in early-generated neurons.

### MiRNAome time trajectories describe cortical development progression

**Figure 4:**
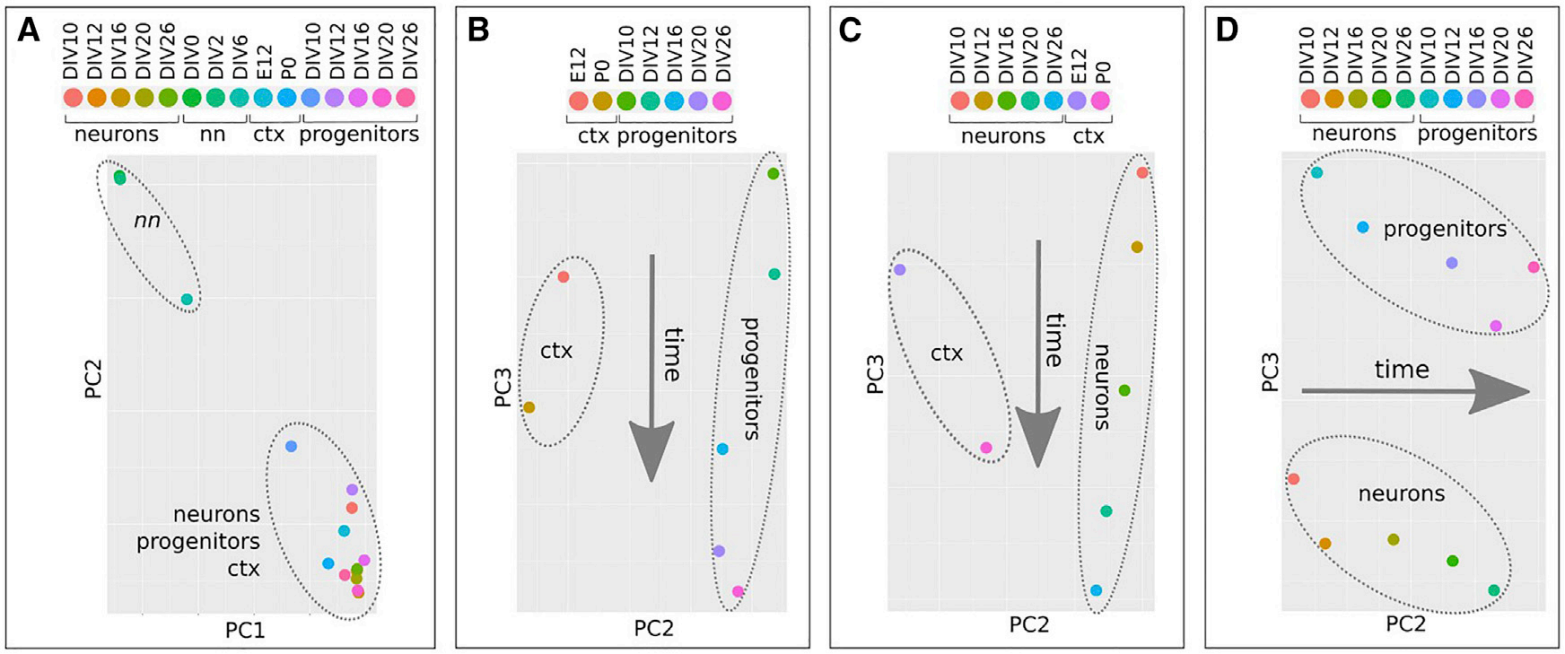
MiRNAome time trajectories in corticogenesis (A–C) PCA of miRNA global profiles of non-neuralized mESCs (nn), neural progenitors (*Sox1*::GFP corticalized mESCs), post-mitotic neurons (Ara-C-treated corticalized mESCs) and mouse cortex (ctx) at different developmental times. Four different combinations of the four groups are shown.

We then set out to identify miRNA candidates regulating *Satb2* expression. With this aim, we sorted *Sox1*::GFP corticalized mESCs, which are enriched in PCs, and first compared their global miRNA profiles with those of non-neuralized mESCs, of post-mitotic corticalized mESCs obtained by AraC treatment, or of mouse cortex, at different developmental times (Figures 4A–4D and Data S3). MiRNAome principal component analysis (PCA) shows high consistency between miRNA profile and cell identity. MiRNAomes of non-neuralized mESCs are well separated from those of corticalized mESCs and of cortex, which instead cluster together, confirming that our *in vitro* protocol mimics a genuine cortical identity (Figure 4A). The time of *in vitro* differentiation distributes both PC (Figure 4B) and neuron (Figure 4C) miRNAomes along PC3, in agreement with the relative position of E12 and P0 cortex miRNAomes, denoting high conservation of the mechanisms accounting for the timing of layer formation in our *in vitro* conditions. Finally, PC3 distinguishes between progenitor and neuron miRNAomes (Figure 4D), indicating that these distinct cell states are maintained throughout the differentiation process.

### Selected miRNAs directly bind *Satb2* 3′UTR in early cortical cells

**Figure 5:**
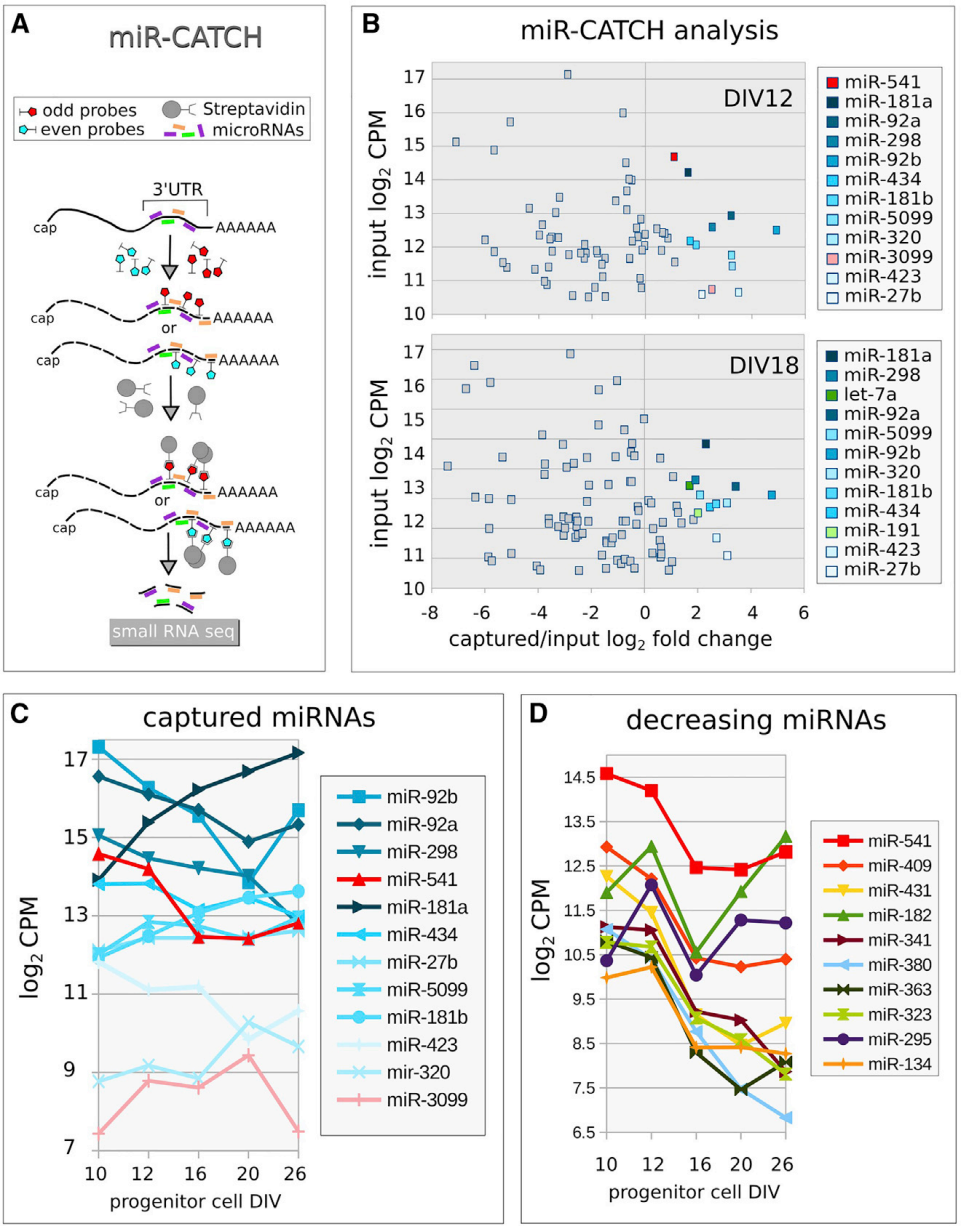
*Satb2* interacting miRNAs (A) Outline of the miR-CATCH method. (B) Enrichment of captured miRNAs (x axis) with respect to input (y axis) at the indicated time. CPM, counts per million. Color labels indicate significantly enriched miRNAs (non-parametric noiseqbio test probability >0.9) (Tarazona et al., 2012). (C) Developmental expression patterns of *Satb2*-captured miRNAs in *Sox1*::GFP PCs. (D) Developmental expression of miRNAs with highest monotonic developmental decrease in *Sox1*::GFP PCs.

To select miRNAs that directly interact with *Satb2* 3′UTR at DIV12 and DIV18, we employed miR-CATCH analysis, which recovers mRNA/RNA-induced silencing complex (RISC)/miRNA complex by biotin-labeled probes complementary to the target mRNA (Marranci et al., 2019; Vencken et al., 2015). Bound miRNAs were quantified through small RNA-seq, and miRNA enrichment was measured with respect to the input (total miRNAs) (Figure 5A). We found that 12 miRNAs bind to *Satb2* mRNA and are significantly enriched at DIV12; of these, miR-541 and miR-3099 are not enriched at DIV18, thus representing candidates for SATB2 inhibition in early, but not late, cortical cells (Figures 5B and S3 and Data S3). Because of its extremely low expression (Figure 5C), we did not further investigate miR-3099 and focused on the other miRNAs.

We analyzed the abundance of the captured miRNAs in PCs and found that only miR-92a/b and miR-541 show robust decrease between DIV12 and DIV16, when SATB2 translation is de-inhibited (Figure 5C). We thus focused our attention on these three miRNAs. miR-92 was already shown to play a major role in inhibiting EOMES (TBR2) translation and preventing early generation of basal PCs, which in mouse give rise to supra-granular neurons (Bian et al., 2013; Nowakowski et al., 2013). Conversely, miR-541 has never been involved in cortical development and belongs to an evolutionary new miRNA cluster (mir-379-mir-410 in mouse, mir-379-656 in humans), located in a large miRNA-containing gene (*Mirg*) inside the *DLK-DIO3* locus (Edwards et al., 2008; Glazov et al., 2008; Winter, 2015) (see discussion). *Mirg* orthologues have been found in all eutherians, which hold inter-hemispheric cortical connections forming the CC, but not in metatherians, prototherians, or any other vertebrates, which lack CC.

miR-541 *in vitro* pattern of expression closely matches the time-dependent inhibition of SATB2 translation and follows a sudden downregulation between DIV12 and DIV16 (Figure 5C). In addition, at E13.5, miR-541 is widely expressed in the ventricular zone (VZ), subventricular zone (SVZ), and mantle zone (MZ), when SATB2 protein is undetectable; at E15.5, the miRNA is expressed in the cortical plate (CP), when the protein is detected in VZ, SVZ, intermediate zone (IZ), and migrating cells (Figure S4). Finally, miR-541 developmental decrease is comparable with that of the most heavily downregulated miRNAs from DIV12 to DIV16 (Figure 5D), strengthening its candidacy for the control of SATB2 inhibition in early corticogenesis.

### miR-541 and miR-92a/b inhibit SATB2 translation in mouse and human early cortical cells

**Figure 6:**
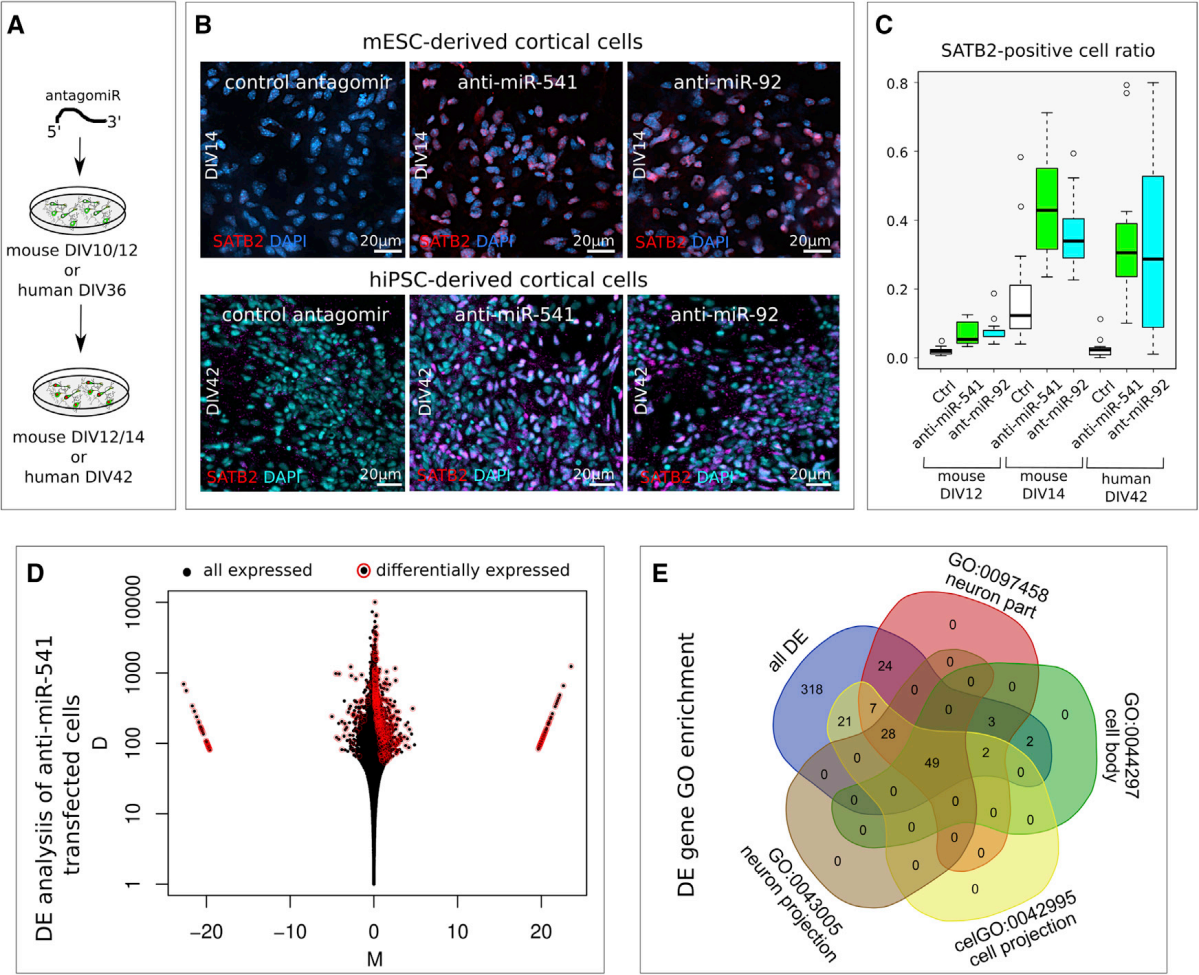
miR-92a/b and miR-541 function in mouse and human cortical cells (A) Outline of the *in vitro* assay of miR-541 inhibition by locked nucleic acid (LNA)-antisense oligonucleotide lipofection in corticalized mESCs (n = 3 independent experiments) or hiPSCs (n = 3 independent experiments). (B) Immunocytodetection shows SATB2-positive nuclei 2 days after mESC lipofection and 6 days after hiPSC lipofection, respectively. (C) Box plots report SATB2-positive nuclei proportion. Ctr, scrambled sequence LNA lipofection. An anti-miR-92a/b LNA oligonucleotide was used to inhibit both miR-92a and miR-92b, which share the seed sequence. (D) Mean-difference plot showing log-fold change (M) and the absolute value of the difference in mRNA expression (D) between antago-miR-541 and control antago-miR transfections (n = 3 independent experiments). (E) Venn diagram showing the distribution of the genes differentially expressed after antago-miR transfection in the four most enriched GO terms.

We then inhibited miR-541 and mir-92a/b by transfection of a complementary locked-RNA (antago-miR) in mouse ES corticalized cultures (Figure 6A). This results in a premature onset of SATB2 protein detection and in a massive increase of SATB2-positive cells compared with control-transfected cells, as found in transfection at DIV10, and in an increase of the efficiency of translation at later time points, as found in DIV12 transfection (Figures 6B top, 6C). Notably, miR-541 has no predicted binding site on *Eomes* 3′UTR; thus, its effect on SATB2 translation is unlikely mediated by increased EOMES translation and consequent induction of basal PC identity (Sessa et al., 2008), as may be the case with miR-92a/b inhibition. We observed similar effects when downregulating miR-541 and miR-92a/b in corticalized human induced pluripotent stem cells (hiPSCs) (Figures 6B bottom, 6C), denoting evolutionary conservation of this control mechanism.

We then transfected antago-miR-541 and control antago-miR at DIV12 and analyzed transcriptomes at DIV17, a time of *in vitro* development when many markers of terminal differentiation can be evaluated. We found that 489 mRNA species significantly change their expression compared with control (Figure 6D and S5 and Data S4). Gene Ontology (GO) analysis of these genes shows that many of them are significantly enriched in terms related to cell differentiation and neuron projection (Figure 6E and Data S4). Notably, many of the genes are involved in CC development and malformation (Data S4).

### GO enrichment of miR-541 target genes

**Figure 7:**
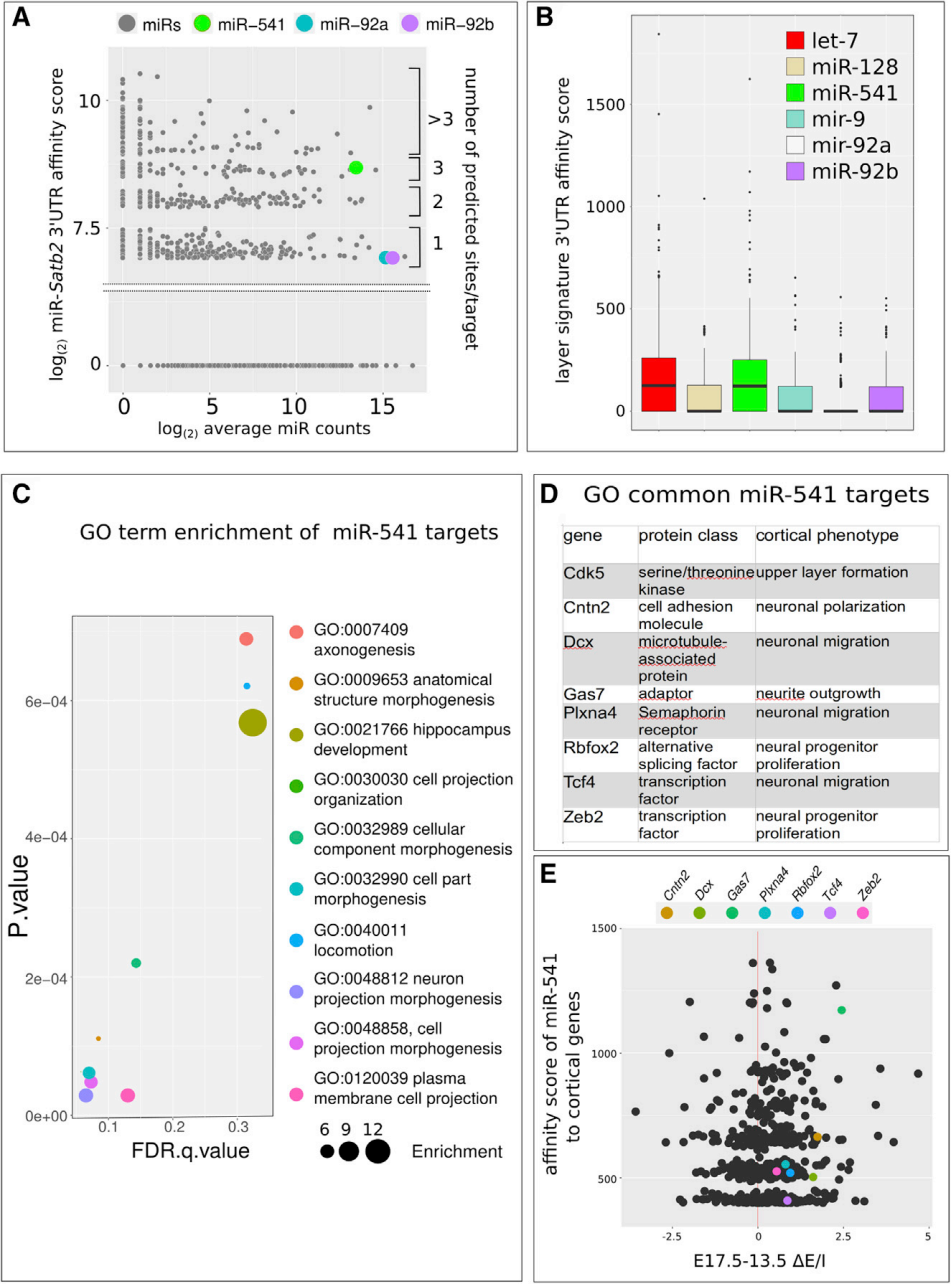
*In silico* analysis of miRNA/mRNA interactions (A) *In silico* comparison of the affinity of mouse miRNAome (gray dots), miR-92a/b, and miR-541 (colored dots) with *Satb2* 3′UTR (Ensembl *Mus musculus Satb2*-201 cDNA 3′UTR), in relation to the average miRNA expression levels during corticogenesis. (B) *In silico* affinity of cortical miRNAs to the 3′UTR of an embryonic cortical layer gene signature (395 genes) (Galfrè et al., 2020). (C) GO enrichment of the mir-541 gene targets with high *in silico* affinity to *Satb2* 3′UTR (cumulative score higher than 400, n = 48) (Enright et al., 2003) with respect to the layer gene signature employed in (B). (D) List of the eight genes common to all the GO terms shown in (C). (E) Plot showing E/I read counts developmental increase (x axis) with respect to miR-541/mRNA affinity score (y axis) to genes of the embryonic cortical marker signature (Galfrè et al., 2020). Colored dots indicate genes listed in (D). Names in labels indicate the five genes with the highest E/I read count ratio increase and mir-541/mRNA affinity score.

To infer the biological relevance of miR-92a/b and miR-541, we evaluated their degree of miRNA-mRNA target affinity using miRanda (Enright et al., 2003) as *in silico* prediction tool. miRanda was more sensitive than TargetScan (Bartel, 2009), TargetSpy (Sturm et al., 2010) and TarPmiR (Ding et al., 2016) in predicting miRNA interactions with *Satb2* 3′UTR (Figure S6A and Data S5), and predicted two sites of miR-541 interaction that were validated by the transfection of a GFP reporter carrying a mutated seed sequence in *Satb2* 3′UTR (Figures S6B–S6D). First, we analyzed the affinity of miRNA-*Satb2* 3′UTR interaction in relation to the average expression of mouse embryonic cortical miRNAs. Among the annotated miRNAs with significant affinity to *Satb2* 3′UTR (Data S6), miR-92a/b and miR-541 show high expression in cortical PCs (miR-92a/b) or high *in silico* affinity to *Satb2* 3′UTR (miR-541) (Figure 7A), in line with their high miR-CATCH enrichment (Figure 5B). miR-541 shares less than half of its targets with miR-92a/b, while miR-92a/b share most of theirs with miR-541 (Figure S6E). We then compared miR-92a/b and miR-541 targets with those of three recently described miRNAs of corticogenesis, namely let7, miR-9, and miR-128 (Shu et al., 2019). For this, we selected a subset of 395 genes associated with an embryonic cortical marker signature (Galfrè et al., 2020). Among the six miRNAs analyzed, let-7 and miR-541 showed *in silico* affinity with more than half of the signature genes (Figure 7B and Data S6), suggesting a more relevant role for them in corticogenesis. Interestingly, among the mRNAs with the highest *in silico* affinity (total score higher than 400) for the six miRNAs, only the putative targets of miR-541 showed significant enrichment in GO terms. It may be notable that terms related to neuronal projection development (axogenesis, neuron projection morphogenesis, cell projection morphogenesis, plasma membrane cell projection) (Figure 7C) are the most represented and that at least eight out of the 11 putative target genes are related to cortical neuronal layering and migration, axon guidance, and CC disturbances (Figure S7). Interestingly, all these 8 genes might be involved in basic processes controlling polarization, proliferation, and migration of late cortical PCs (Figure 7D; Figure S7 and references therein). Figure 7E compares the change of E/I read counts by EISA of seven out of the eight genes (not enough *Cdk5r* read counts were available for a significant analysis) with those of the genes of the embryonic cortical marker signature. The results indicate that all these seven genes increase their E/I read count ratios between E13.5 and E17.5 and that there is a general correlation between E/I read count increase and mir-541/mRNA affinity score, supporting a relevant role of miR-541 in their post-transcriptional control during early corticogenesis.

## Discussion

Translational control exerted by RNA-binding proteins or miRNAs plays a crucial role for the appropriate time of production of key proteins that govern the potential of cortical PCs as well as the differentiative program of the post-mitotic neurons (Kosik and Nowakowski, 2018; Nowakowski et al., 2018; Shu et al., 2019; Zahr et al., 2018). For example, cortical PCs express *Brn1* and *Tle4* mRNAs, for both deep and superficial layer fates, respectively, but translation into their corresponding proteins is initially repressed and subsequently released in due time (Zahr et al., 2018). miRNAs are especially interesting as heterochronic modulators of vertebrate development (Gulman et al., 2019; Robinton et al., 2019), also in the nervous system (Chiu et al., 2014; Nowakowski et al., 2018; Zahr et al., 2019).

In this paper, we found evidence for differential stability of *Satb2* mRNA compared with other key mRNAs. SATB2 protein plays a central role in cortical neurogenesis, both in the early embryo and at later postnatal stages. Early *Satb2* inactivation leads to absence of CC, with upper layer neurons diverting their axons to subcortical targets (Alcamo et al., 2008; Britanova et al., 2008; Leone et al., 2015; McKenna et al., 2015; Srinivasan et al., 2012). Conversely, later *Satb2* inactivation leaves the CC intact, although there are effects on plasticity and long-term memory storage (Jaitner et al., 2016). Finally, *Satb2* also plays a role in layer V subcortical projection neurons (Srinivasan et al., 2012). These data indicate that SATB2 acts in a cell context- and time-dependent multifaceted way, and that precise control of its expression may be relevant for cortical development. Significantly, Paolino et al. (2020) have shown that accurate timing of SATB2 protein appearance in mouse is crucial for axonal projection of layer II–III neurons through the CC. In fact, while SATB2 protein is readily translated from its mRNA in the dunnart marsupial model (where layer II–III axons travel through the anterior commissure and the CC is absent), in the mouse SATB2 protein appearance is delayed with respect to its mRNA expression (and axons go through the CC). Strikingly, anticipated SATB2 protein production in the mouse reroute layer II–III commissural axons toward the anterior commissure instead of the CC. Thus, a post-transcriptional control may be relevant in timing SATB2 protein appearance within the developing early placental neocortex (Paolino et al., 2020).

Our results provide evidence that *Satb2* 3′UTR contributes to this control. *Satb2* 3′UTR drives a significant translational inhibition of a GFP reporter at an early (DIV12), but not at a late (DIV18), stage of *in vitro* differentiation. Moreover, it is bound by the AGO/RISC complex in a much stronger way at an early (DIV12) than at a late (DIV18) stage, suggesting its regulation by miRNAs. We identified miR-92a/b and miR-541 as candidate miRNAs to modulate SATB2 onset of translation, on the basis of their temporal dynamics of expression and of direct miR-CATCH biochemical selection. Significantly, antagonizing these miRNAs anticipates the appearance of SATB2-positive cells in both mESCs and hiPSCs induced to cortical differentiation *in vitro*. While the antagonism of miR-92a/b might exert this effect by anticipating the translation of EOMES (TBR2), and then the differentiation of intermediate PC progeny expressing SATB2 (Bian et al., 2013; Nowakowski et al., 2013), miR-541 has no predicted binding sites on *Eomes* mRNA. Thus, miR-541’s effect on the onset of appearance of SATB2-positive neurons is directly due to its binding to *Satb2* 3′UTR. miR-541 is likely targeting a high number of genes, as suggested by the transcriptome change observed after its inhibition by antago-miR transfection and the *in silico* analysis of its targets.

Unlike miR-92a/b, let-7b, miR-128, and miR-9, and other evolutionarily conserved miRNAs involved in cortical development (Chiu et al., 2014; Nowakowski et al, 2013, 2018; Shu et al., 2019; Zahr et al., 2018), mir-541 is only present in eutherians (see below) and the only functional report shows its role in inhibiting neurite growth in PC2 cells (Zhang et al., 2011). miR-541 expression declines during corticogenesis in a temporal pattern opposite to that of SATB2 protein, and its presence in eutherians, but not in metatherians or any other vertebrates, suggests that it might be involved in the heterochronic shift of SATB2 translation between dunnart and mouse (Paolino et al., 2020). Our demonstration that miR-541 can bind *Satb2* 3′UTR and inhibit translation both *in vitro* and *in vivo* provides a molecular mechanism contributing to this heterochronic shift.

Together with about 40 miRNAs, miR-541 is encoded by *Mirg* (miRNA-containing gene), present only in eutherians inside the *Dlk1-Dio3* locus (Edwards et al., 2008; Glazov et al., 2008; Marty and Cavaillé, 2019; da Rocha et al., 2008; Winter, 2015). *Mirg* mRNA was detected in the developing early nervous system and in other organs, including the liver (Han et al., 2012). Constitutive *Mirg* deletion affects energy homeostasis, causing neonatal lethality (Labialle et al., 2014), and behavioral disturbances (Lackinger et al., 2019; Marty et al., 2016). However, the overall role of *Mirg* and its individual miRNAs in the early nervous system and cortical layering has not been deeply defined, with few exceptions (Marty and Cavaillé, 2019; Winter, 2015). For some of these miRNAs, a neurogenic function has been shown or proposed, but several seem involved in brain disorders (Gallego et al., 2016; Shi et al., 2015; Tsan et al., 2016; Winter, 2015). An overall GO analysis of the targets of these miRNAs pointed to embryonic and neural development and especially at axon guidance as key enriched terms; the possible involvement of *Mirg* in the regulation of key factors for CC formation was suggested by *in silico* target analysis (Glazov et al., 2008). It may be notable that mRNAs for axon guidance molecules, identified as targets of other miRNAs of *Mirg* (Glazov et al., 2008), are also *in silico* targets of miR-541; conversely, some of miR-541’s most relevant targets (Figure S7) are also targets of other miRNAs of *Mirg*. Thus, the coordinate action of *Mirg* miRNAs in endowing the eutherian brain with some of its characters is an attractive hypothesis.

*Satb2* is present in all vertebrates (Sheehan-Rooney et al., 2010) and is expressed with other CITF genes in the dorsal telencephalon (pallium) of birds, reptiles, and mammals, although with different patterns of mutual co-expression (Nomura et al., 2018; Tosches and Laurent, 2019). In the early mammalian neocortex, SATB2 efficiently binds the *Bcl11b* promoter and prevents its expression, although at later stages LMO4 relieves this inhibition (Alcamo et al., 2008; Britanova et al., 2008; Harb et al., 2016). In contrast, in reptilian and avian pallial cells, SATB2 cannot silence *Bcl11b*, due to inefficient binding to *Bcl11b cis*-regulatory sequences, and SATB2 and BCL11B are co-expressed (Nomura et al., 2018). By partitioning these two proteins in separate layers, this change may have increased cortical heterogeneity in the mammalian brain (Nomura et al., 2018). It looks possible that, on top of mutual transcriptional regulation, heterochronic gene modulation also takes place in brain development. Notably, in higher primates, SATB2 appearance is delayed over an extended period, possibly crucial for cortical expansion, during which deep layer neurogenesis is balanced with the expansion of PCs (Otani et al., 2016). Altogether, these observations indicate that tight temporal control and initial repression of SATB2 expression (Paolino et al., 2020) (present work) may hold a crucial role in pallial evolution.

## Experimental procedures

mESC corticalization *in vitro*, cell transfection, and analysis were performed as previously described (Terrigno et al., 2018a, 2018b). hiPSCs (ATCC-DYS0100 line, American Type Culture Collection) were neuralized according to Chambers et al. (2009).

COTAN was performed on previously published datasets (Yuzwa et al., 2017) according to Galfrè et al., (2020). EISA was performed as described (Gaidatzis et al., 2015; La Manno et al., 2018) on available datasets (Chui et al., 2020). RNA immunoprecipitation, small RNA-seq, and miR-CATCH were carried out as described (Marranci et al., 2019; Pandolfini et al., 2016), with minor modifications.

miRNA-mRNA *in silico* affinity was predicted as described (Enright et al., 2003), using score >120, energy < −18 kcal/mol as thresholds. 3′UTR sequences were obtained from Ensembl resources (Hunt et al., 2018), using Cran Biomart package. miRNA sequences were obtained from miRBase database (v.22) (Kozomara et al., 2019). Detailed material and methods are described in supplemental information.

### Data and code availability

The accession numbers for the sequencing data reported in this paper are GEO: GSE172502 (small RNA-seq) and GEO: GSE172503 (RNA-seq).

## Author contributions

M.M., R.V., L. Poliseno, and F.C. designed the experiments. M.M. performed cell culture, molecular biology, imaging, and gene expression data computation. P.M. and I.A. planned and carried out IUE. S.G. and F.M. performed COTAN and EISA. M.P. and M.H.C. advised on computational methods of COTAN, EISA, and small RNA-seq analysis. K.D. performed hiPSC cultures. M.T. and L. Pandolfini. performed small RNA-seq. L. Pandolfini. carried out AGO RIP and RNA-seq. M.R. and A. Marranci carried out miR-CATCH. A. Mercatanti performed the computational analysis of captured miRNAs. R.V. and F.C. wrote the paper.

## Declaration of interests

The authors declare no competing interests.

## Supplemental information

Document S1. Supplemental experimental procedures, Figures S1–S7, and Table S1

Data S1. COTAN coex-values and p-values of selected genes

Data S2. EISA

Data S3. microRNA read counts

Data S4. Gene DE and GO enrichment of antagomiR-transfected cells

Data S5. Comparison of mRNA/miRNA in silico interaction predictors

Data S6. microRNA targeting analysis

Document S2. Article plus supplemental information

## Footnotes

Supplemental information can be found online at https://doi.org/10.1016/j.stemcr.2021.04.020.
