# Supplementary material for: A eutherian-specific microRNA controls the translation of Satb2 in a model of cortical differentiation
Source: Stem Cell Reports. 2021 May 20;16(6):1496–509. doi: 10.1016/j.stemcr.2021.04.020 (PMC8190598; doi:10.1016/j.stemcr.2021.04.020)
Supplement: Document S1. Supplemental experimental procedures, Figures S1–S7, and Table S1 [file mmc1.pdf]

Stem Cell Reports, Volume *16*

Supplemental Information

A eutherian-specific microRNA controls the translation of Satb2 in a model of cortical differentiation

Manuella Martins, Silvia Galfrè, Marco Terrigno, Luca Pandolfini, Irene Appolloni, Keagan Dunville, Andrea Marranci, Milena Rizzo, Alberto Mercatanti, Laura Poliseno, Francesco Morandin, Marco Pietrosanto, Manuela Helmer-Citterich, Paolo Malatesta, Robert Vignali, and Federico Cremisi

Supplemental Figures

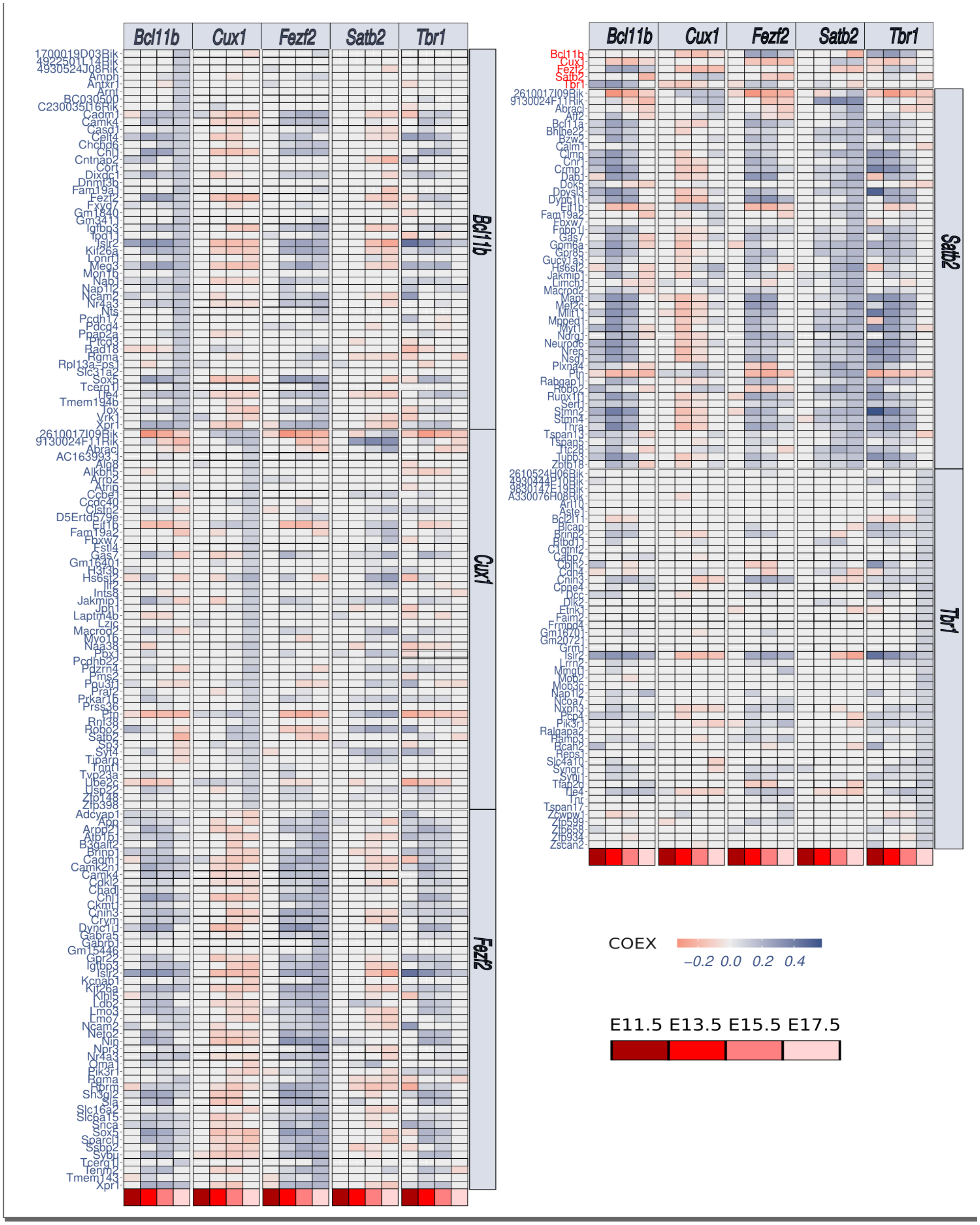

Figure S1. COEX values of genes related to CITFs, Related to Figure 1. Heatmap shows details of Figure 1D.

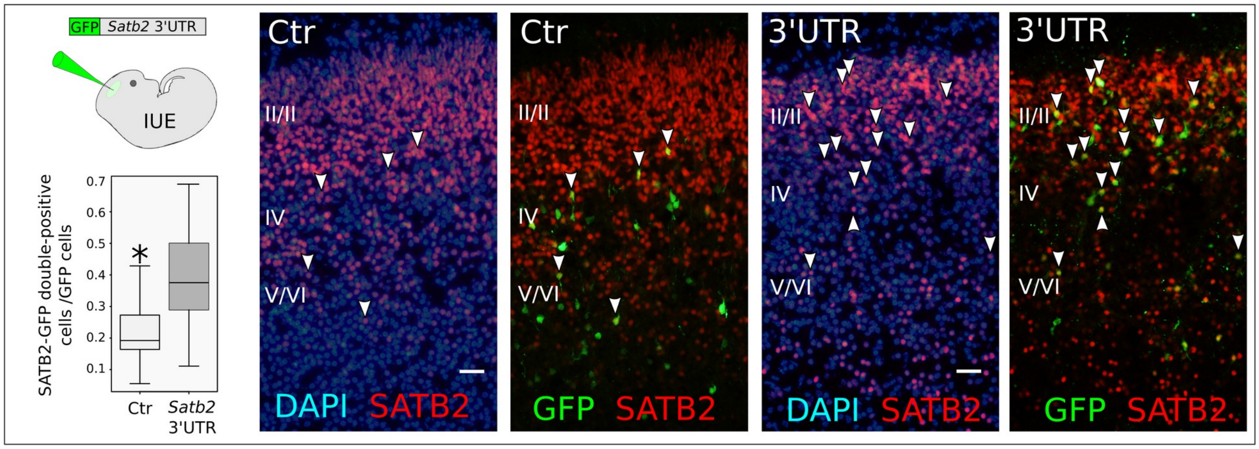

Figure S2. Expression of a GFP reporter bearing *Satb2* 3' UTR, Related to Figure 3. IUE at E13.5 of a reporter as in the scheme. Box plot indicates the ratio of GFP-SATB2 double-positive cells out of all GFP-positive cells. Pictures show examples of IUE cells at E19. Arrowheads point to GFP-SATB2 double positive cells. Roman numbers indicate layers. Scale bar, 50 µm. Data from n= 3 animals for control IUE and n=3 animals for 3' UTR IUE.

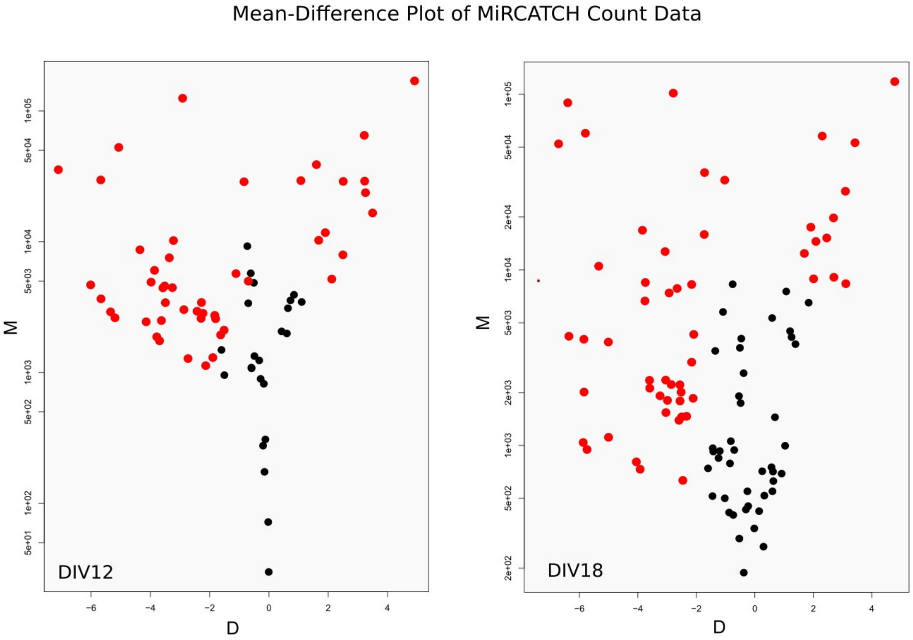

Figure S3. miR-CATCH M-D plot, Related to Figure 5. The Mean (M) - Difference (D) plot reported in figure shows the results of miRNA capturing by miR-CATCH as obtained by analysis with the NOIseqbio R package. Plots show the distribution of miRNAs enriched (positive $\log_2$ fold change, D) or depleted (negative $\log_2$ fold change) after miR-CATCH capturing, with respect to average expression (M, mean CPM), at the indicated time of differentiation (DIV12, DIV18). In red, miRNAs with significant fold change (probability > 0.9).

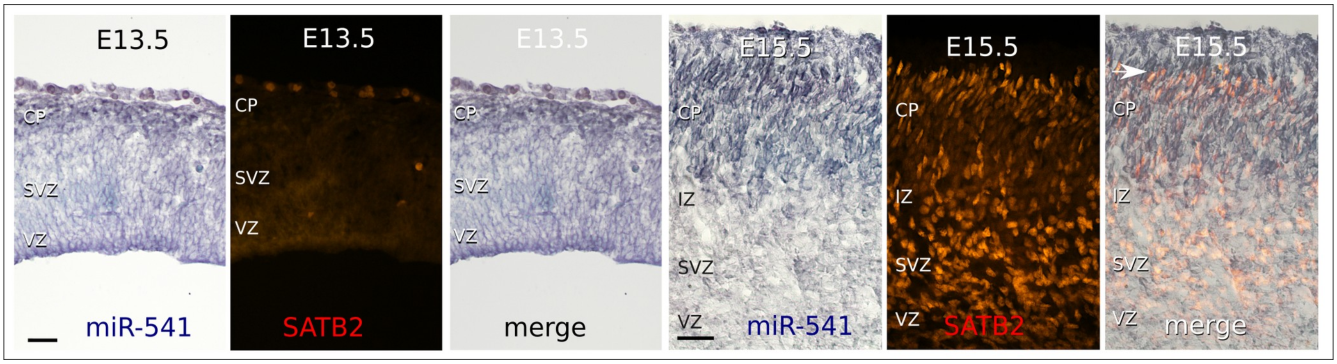

Figure S4. mir-541 expression, Related to Figure 5. *In situ* hybridization shows miR-541 distribution (BM-purple staining) compared to SATB2 immunodetection (red fluorescence). CP, cortical plate; IZ, intermediate zone; SVZ, subventricular zone; VZ, ventricular zone. The arrow indicates newly migrated SATB2-positive cells. Scale bar, 50 µm.

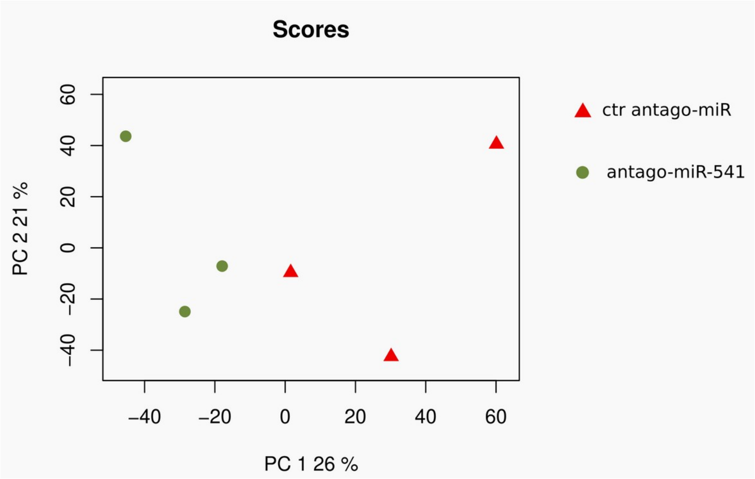

Figure S5. Transcriptome PCA, Related to Figure 6. PCA of corticalized ES cells transfected with antago-miR-541 and control antago-miR at DIV12 and analyzed by RNA-seq at DIV17. N= 3 independent experiments. The first component discriminates between control and experimental cells.

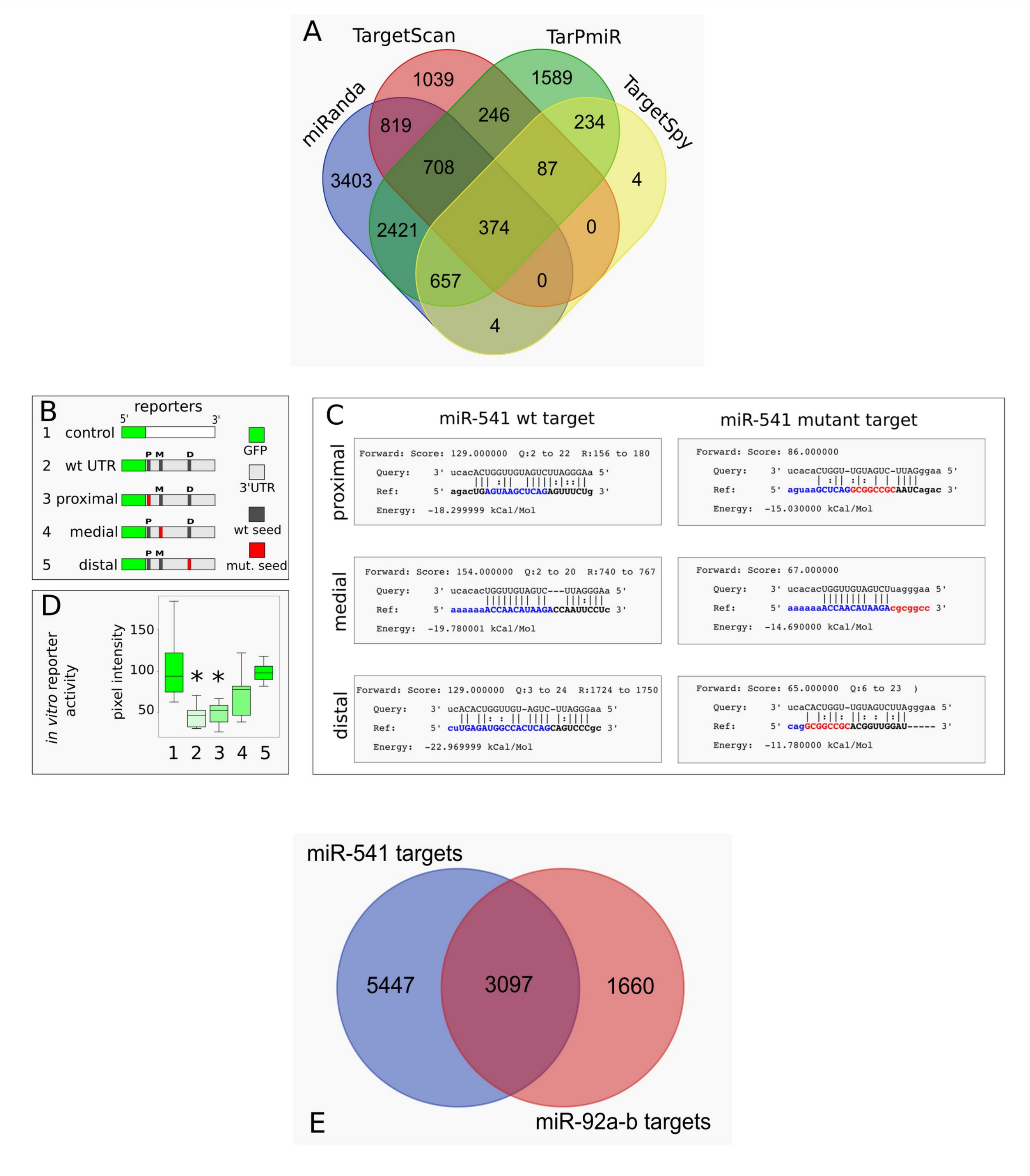

Figure S6. microRNA target analysis, Related to Figure 7. A**,** Venn diagram shows the intersections between genes targeted *in silico* by miR-541 as predicted by the four indicated tools (see Data S5). miRanda was compared to: TargetScan, which especially rewards the seed region (Bartel, 2009); TargetSpy, which predicts targets regardless of the presence of a seed match (Sturm et al., 2010); the ultimate tool TarPmiR, which can utilize miRNA-mRNA binding experiment data such as CLASH and applies a random-forest-based approach to integrate both conventional and new features (Ding et. al, 2016). B-D, GFP reporter analysis of *Satb2* 3'UTR**.** B, bars outline the reporter sequences used in the analysis of the *Satb2* 3' UTR. Wt and mutated sites of miRNA-mRNA interaction predicted by miRanda are depicted in black and red, respectively. The relative position of GFP

coding sequence is in green. C, panels show the interactions predicted by miRanda between mmu-miR-541-5p and *Satb2* 3'UTR outlined in B. Score estimates the probability of interaction, energy reports the predicted chemical binding affinity. Query: miRNA sequence. Ref: UTR sequence, seed in red, upstream bases of interaction in blue. D, box plots report GFP pixel intensity in DIV 12 cells (3 independent experiments), 2 days after transfection of the constructs shown in B. Asterisks indicate significant decrease compared to control (Wilcoxon test). E. Venn diagram shows the intersection between miR541 and miR92a-b gene targets as predicted by miRanda.

Name	Biochemical role	Biological process	Phenotype	References
Gas7	Adaptor protein	Neurite outgrowth, growth arrest	AD, schizophrenia	doi:10.1074/jbc.M113.513119<br>doi:10.1073/pnas.95.19.11423<br>doi: 10.1074/jbc.M109.051094<br>doi: 10.1186/s13041-016-0238-y
Rbfox2	RNA binding protein	Neuronal mRNA splicing		doi:10.1128/MCB.25.22.10005–10016.2005<br>doi:10.1371/journal.pcbi.0030196<br>doi.org/10.1016/j.neuron.2018.01.020
PlexinA4	Semaphorin receptor	Cortical neuronal migration, axon guidance		doi: 10.1016/j.isci.2019.10.034<br>doi:10.1523/JNEUROSCI.4480-04.2005
Cntn2	GPI-linked member of the immunoglobulin superfamily, cell adhesion molecule	Neurite outgrowth, axonal adhesion	Smaller cortex, reduced corticothalamic axons, callosal and commissural defects	doi: 10.3389/fncel.2019.00454
Dcx	Brain-specific microtubule associated protein	Cytoskeletal regulator involved in migration, cortical layering, neurite extension	Lissencephaly, subcortical band heterotopia, epilepsy, cognitive disability	doi:10.1016/s0092-8674(00)80898-3 et al., 1998;<br>doi:10.1038/mp.2017.175
Zeb2	Transcription factor	Regulation of gene expression	Mowat-Wilson syndrome; defects in axonal growth; hippocampal and callosal defects	doi: 10.1093/hmg/ddv350<br>doi:10.1016/j.neuron.2015.01.018
Cdk5	Serine/threonine kinase	phosphorylation of various cytoskeletal proteins	Corticogenesis, neuronal migration, axonal projections, dendrite branching; callosal dysgenesis	doi: 10.1073/pnas.93.20.11173<br>doi: 10.1523/JNEUROSCI.18-16-06370.1998<br>doi:10.1242/dev.02854<br>doi:10.1523/JNEUROSCI.1014-07.2007
TCF4	Transcription factor	Regulation of gene expression	Pitt-Hopkins syndrome; schizophrenia; autism; callosal dysgenesis	doi: 10.1086/515582<br>doi:10.1007/s00439-011-1020-y<br>doi:10.1093/schbul/sbx164<br>doi: 10.1111/ejn.14674

Figure S7. miR541 target genes enriched in GO analysis, Related to Figure 7.

Supplemental Experimental Procedures

Mouse ES cell-derived neural cell cultures

Murine ES cell lines E14Tg2A (passages 25-38) and 46 C (transgenic *Sox1*-GFP ESC kindly provided by A. Smith, University of Cambridge, UK, passages 33–39) were used for *in vitro* corticalization. For expansion, ES cells were grown on gelatin-coated tissue culture dishes (pre-treated 10 minutes with 0.1% gelatin in PBS) at a density of $4x10^4$ cells/cm^2. ES cell medium, changed daily, contained GMEM (G5154, Sigma-Aldrich), 10% Fetal Calf Serum (12133C, Sigma-Aldrich), 2mM Glutamine (25030, ThermoFisher Scientific), 1mM sodium Pyruvate (25030, ThermoFisher Scientific), 1mM non-essential amino acids (NEAA, 11140, Sigma Aldrich), 0.05mM β-mercaptoethanol (M3148, Sigma Aldrich), 100 U/mL Penicillin/Streptomycin (15140, ThermoFisher Scientific) and 1000 U/mL recombinant mouse LIF (PMC9484, ThermoFisher Scientific). Chemically defined minimal medium (CDMM) for neural induction consisted of DMEM/F12 (21331-046, ThermoFisher Scientific), 2mM Glutamine, 1mM sodium Pyruvate, 0.1Mm NEAA, 0.05mM β-mercaptoethanol, 100 U/mL Penicillin/Streptomycin supplemented with N-2 Supplement 100X (175020, ThermoFisher Scientific), and B-27 Supplement minus Vitamin A 50X (125870, ThermoFisher Scientific). ES neuralization was performed in three steps. In Step-I, dissociated ES cells were washed with DMEM/F12, seeded on gelatin-coated culture dishes ($6.5x10^4$ cells per cm^2) and cultured in CDMM plus 2.5μM 53AH Wnt inhibitor (C5324-10, Cellagen Technology) and 0.25μM BMP inhibitor (SML0559, Sigma Aldrich), for 3 days. In Step-II, ES cells were dissociated and seeded ($6.5x10^4$ cells per cm^2) on Poly-ornithine (P3655 Sigma-Aldrich; 20 μg/mL in sterile water, 24 hours coating at 37°C) and natural mouse Laminin (23017015, ThermoFisher Scientific; 2.5 μg/mL in PBS, 24 hours coating at 37°C). Cells were cultured for 4 additional days in CDMM plus Wnt/BMP inhibitors, with daily medium change. Serum employed for Trypsin inactivation was removed by two washes in DMEM/F12. In Step-III, cells were dissociated and seeded ($1.25x10^5$ cells per cm^2) on Poly-Ornithine and Laminin coated wells. Subsequently, isocortical cultures were kept in CDMM plus Wnt/BMP inhibitors for four additional days. On the eleventh day of differentiation, DMEM/F12 was replaced with Neurobasal and NEAA were removed from the CDMM to avoid glutamate-induced excitotoxicity. Medium was changed daily until the day of cell fixation.

hiPSC-derived neural cell cultures

Neural cell cultures were differentiated from a commercial reprogrammed fibroblast line (ATCC-DYS0100 line, American Type Culture Collection). Cell neuralization was carried out essentially as described (Chambers et al., 2009), with minor modifications. Reprogrammed stem cells were seeded at $3x10^4$ cells/cm^2 cultured on 1:100 geltrex and maintained in Essential 8 medium for two days. After two days incubation, cultures were switched to neural differentiation media: DMEM/F12 1:1 (21331-046, ThermoFisher Scientific) containing 2mM Glutamine (25030, ThermoFisher Scientific), 1mM Sodium Pyruvate (11360070, ThermoFisher Scientific), 100 U/mL Penicillin-streptomycin (15140, ThermoFisher Scientific), 1mM Non-essential amino acids (11140, Sigma Aldrich), 0.05mM β-mercaptoethanol (M3148, Sigma Aldrich), 10μM 53AH (C5324-10, Cellagen Technology), 10μM LDN193189 hydrochloride (SML0559, Sigma Aldrich), 1μM RepSox (R0158, Sigma Aldrich), N-2 Supplement 100X (175020, ThermoFisher Scientific), and B-27 Supplement minus Vitamin A 50X (125870, ThermoFisher Scientific). After 10 days in neural differentiation medium, cells were displaced from substrate via incubation at 37ºC for 20 minutes in Accutase solution (A6964, Sigma Aldrich). Cells were harvested, diluted in 5 volumes of 1X PBS, centrifuged for 4 minutes, and replated at 10^5 cells/cm^2 on poly-ornithine (P3655, Sigma Aldrich)/recombinant human Laminin (AMS.892 021, Amsbio) in half volume of neural differentiation media + 5 μM Y-27632 (SM02, Cell Guidance Systems). Cells were maintained for 4 days in fresh neural differentiation media without ROCK inhibitor followed by an expansion of 7 days in neural differentiation media without TGFβ, WNT, and BMP inhibitors. After 11 days, cells were displaced again from substrate via incubation at 37ºC for 20 minutes in Accutase solution. Cells were harvested, diluted in 1X PBS at a volume 5 times that of Accutase, centrifuged for 4 minutes, and replated at $2.5x10^5$ cells/cm^2 on poly-ornithine (P3655, Sigma Aldrich)/purified mouse Laminin (CC095-M, Merck Millipore) in Eppendorf glass bottom dishes (H 0030 741 021, Eppendorf). Cells were maintained in neural differentiation media without inhibitors for 12 days and then switched to neuronal maintenance media based on Neurobasal (21103049, ThermoFisher Scientific) and containing 2mM Glutamine (25030, ThermoFisher Scientific), 1 mM Sodium Pyruvate (11360070, ThermoFisher Scientific), 100 U/mL Penicillin-streptomycin (15140, ThermoFisher Scientific), 0.05mM β-mercaptoethanol (M3148, Sigma Aldrich), Ascorbate, 0.5mM (A92902, Sigma Aldrich), Recombinant human BDNF, 20 ng/mL (NBP2-52006, Novus Biologicals), and B-27 Supplement minus Vitamin A 50X (125870, ThermoFisher Scientific) until fixation at DIV 42. Cells were fixed with 2% PFA warmed to 37ºC for 15 minutes at room temperature.

Cell transfection

Plasmid transfections in mouse cortical cells were performed in 24-multiwell plate using 1 μg plasmid DNA diluted in 2.5 μL/well of Lipofectamine 2000 (12566014, ThermoFisher Scientific) in a final volume of 0.5 mL/well

OPTI-MEM (31985062, ThermoFisher Scientific). Reporter activity plasmids were pEGFP-C1 (Clontech; control) and pEGFP-C1 fused to 3' UTR of *Satb2* between HindIII and XbaI sites.
LNA anti-miRNA (antagoMir) transfections in mouse cortical cells were performed using Lipofectamine 2000 according to the manufacturer's instructions. miRCURY LNA™ microRNA Inhibitors to miR-541-5p, miR92-3p and control antagomiR (MIMAT0003170, YI00199006 and MIMAT0000539, respectively) were resuspended in TE buffer (10mM Tris pH 7.5, 1mM EDTA) to a final concentration of 50μM. Cells were transfected in 24-well plate using 25pmol of LNA diluted in 2.5 μL/well of Lipofectamine 2000 in a final volume of 0.5 mL/well OPTI-MEM. After transfection, cells were incubated at 37°C and 5% CO2 for 4-6 hours and then the medium was replaced with complete Neurobasal medium (mouse cortical cells) or complete McCoy medium (HCT-116 cells).

Satb2 3'UTR cloning

The entire *Satb2*-3'UTR sequence (2802 bp) was obtained from Genome Reference Consortium Mouse Build 38 patch release 6 (GRCm38.p6) and amplified by PCR with Q5 High-Fidelity DNA Polymerase (M0491, NEB) with a forward and reverse primer carrying, correspondingly, a HindIII and XbaI restriction site at their 5'end (forward, CACAAAGCTTGTGAACTCCGCAGGCAGAGC; reverse, CACATCTAGAGCGTTTTATTTAACAACCAAAAAATTCTAACAGCC). The plasmid carrying the *Satb2*-3'UTR was constructed using mammalian expression vector pEGFP-C1 (Clontech) cut at HindIII position and XbaI positions inside the multiple cloning site and ligated with HindIII/XbaI restricted amplification product by T4 DNA ligase (M0202, NEB).
In order to identify potential target sites for our miRNA of interest (mmu-miR-541-5p) in the *Satb2* sequence, miRanda algorithm (v3.3a) (27) was used. MiRanda selected miR-541/*Satb2* binding sites with score >120 and energy < -18kd. Mutations in the three predicted sites were performed. The seed sequence of miR-541 at +156, +740 and +1724 were replaced with NotI restriction sequence (GCGGCCGC). To this aim, upstream and downstream halves of mutated 3'UTR were generated by PCR through external forward or reverse primer NotI together with a mutated internal reverse of forward primer, respectively. The mutated internal primers for miR-541 mutation at position +156 were miR-541_mutA_fw CACAGCGGCCGCAATCAGACGTCACCTTGGCAAAG and miR-541_mutA_rev CACAGCGGCCGCCTGAGCTTACTCAGTCTATAGGCTATCCTGTG. The mutated internal primers for miR-541 mutation at position +740 were mir-541_mutb_fw CACAGCGGCCGCCAGAGGACATAATGCACACCTTAAGAC and miR-541_mutB_rev CACAGCGGCCGCGGTCTTATGTTGGTTTTTTTGACATGCCC. The mutated internal primers for miR-541 mutation at position +1724 were miR-541_mutC_fw CACAGCGGCCGCGAGTTGTATCCTCATGCAACCTTGTC and miR-541_mutC_rev CACAGCGGCCGCCTGAGTGGCCATCTCAAGCC. After PCRs, both upstream and downstream mutated halves were digested with NotI enzyme, ligated and used as a template for PCR together with external forward and reverse primers (forward, CGCAGGCAGAGCAATAGATGG; reverse, GGCGGGAAATTGTGCTTTGTCAAGA). PCR products were cut with HindIII/XbaI restriction enzymes, purified and re-inserted in the pEGFP-C1 vector.

Immunocytodetection (ICD) and imaging

Cells prepared for immunocytodetection experiments were cultured on Poly-Ornithine/Laminin coated round glass coverslips. Cells were fixed using 2% paraformaldehyde for 12 minutes, washed twice with PBS, permeabilized using 0.1% Triton X100 in PBS and blocked using 0.5% BSA in PBS for 1 hr at RT. Embryonic cortical sections were thawed and let dry at room temperature 1 hr, then briefly washed three times (5 minutes each) in PBS before antibody staining.
Cells/slices were pre-treated 1 hr at RT with blocking solution: 1% BSA, 10% goat serum, 0.1% Triton X100 in PBS. Primary antibodies used for microscopy were SATB2 ab (1:1000; ab92446, Abcam), GFP ab (1:1000, ab13970, Abcam). Primary antibodies were incubated overnight at 4°C in PBS containing 1% BSA and 10% goat serum in PBS; cells/slices were then washed three times with PBS (10 minutes each). Alexa Fluor 488 and Alexa Fluor 546 anti-mouse, anti-rabbit or anti-chicken IgG conjugates (1:500; A32723, A-11034, A-11039, A-11003, A-11010, A11040, Molecular Probes) were incubated 1 hour at RT in PBS containing 1% BSA and 10% goat serum, followed by three PBS washes (10' each). Nuclear staining was obtained with DAPI (D1306, ThermoFisher Scientific). Cells/slices were coverslipped with Aqua Poly-mount (18606-100, Polysciences).
Mouse neural cells were imaged using a Nikon Eclipse E600 epifluorescence microscope with a 20X objective and a Photometrics Coolsnap CF camera. Five to ten optic fields from two or more biological replicates were acquired. In the experiments of EGFP pixel intensity quantification, all the pictures were acquired with the same parameters and the median of pixel intensity of the entire acquired field was analysed. For cell counting, double blind analysis was performed.
Human neural cells were imaged using a Leica SP2 confocal microscope with a 40X oil objective. Z-stacks were attained between 9-12 μm thick optical sections. Three biological replicates were attained per treatment group

and subdivided into 5 technological replicate Z-stacks resulting in 15 total acquisitions. Stacks were flattened in ImageJ (RRID:SCR_002285) using the Z-stack projection function, set as a representation of standard deviation, and backgrounds were subtracted as a function of disabled smoothing and rolling ball radius of 20 px2. The resultant Hoechst+ and SATB2+ images were then subjected to an automated cell counter in ImageJ macros which analyzed separate channels at a 16-bit threshold set between 30-65355, and individual cells were counted using the "Analyze Particle" function set at circularity 35-150 px2 and circularity 0.33-0.99 to only include positive nuclei and minimize false positives.

ScRNA-seq datasets

ScRNA-seq datasets available in literature (Yuzwa et al., 2017) were used to analyse cortical gene expression at E11.5, E13.5, E15.5 and E17.5. Raw counts were obtained from GEO:GSE107122 and used to plot counts/cell values by the vioplot R package.

COTAN

Co-expression Table Analysis (COTAN) aims to estimate the UMI detection efficiency (UDE) of each cell, finds an approximation of the probability of zero read counts for a gene in a cell, and test the null hypothesis of independent expression for gene pairs, by counting zero/non-zero UMI counts in single cells (co-submitted paper). Briefly, mitochondrial genes and genes expressed in less than 0.3% of cells were eliminated. UDE for each cell and average expression for each gene were estimated as described (Galfrè et al. 2020) (*linear* method was used). PCA and hierarchical clustering (two clusters) were then carried out on UMI counts normalized dividing them by UDE. After removal of cell outliers resulting from PCA and hierarchical clustering, UDE and average expression were estimated again. Cells with very low UDE values were also removed. Together the two cleaning steps removed in all the datasets less than 3% of the cells (E11.5 dropped from 1,418 cells to 1,379 cells, E13.5 dropped from 1,137 to 1,119, E15.5 dropped from 2,955 to 2,921, E17.5 dropped from 880 to 863 cells).

Expected values for contingency table analysis were obtained as described (Galfrè et al. 2020) using cells UDE and genes average expression estimated with linear method, and genes dispersion estimated by fitting the observed number of cells with zero UMI count. COTAN then provided both an approximate p-value for the test of independence and a signed co-expression index (COEX), which measures the direction and intensity of the deviation from the independence hypothesis. The heatmaps in Figure 1D are colored by COEX value (blue for co-expression and red for disjoint expression).

For each gene, GDI was computed by normalizing P, the 0.001 quantile of the p-values of COTAN test for co-expression with all other genes. Our chosen normalization is ln(-ln(pval)). Genes with GDI > 2.2, which corresponds to $\ln(-\ln(10^{-4}))$, were generally non constitutive genes (Galfrè et al., 2020). Plots were generated with ggplot2 in R environment. The following R packages were employed: matrixStats, ggfortify, dplyr, rray, propagate, data.table, ggsci, gmodels, parallel, tibble, ggrepel.

scRNA-seq bidimensional analysis

UMI counts were divided by COTAN UDE for normalization. PCA was performed with normalized counts in R environment. Eigenvalues were plotted for selection by "elbow" point analysis (the number of components used were: 10 for E11.5, 10 for E13.5, 15 for E15.5 and 10 for E17.5). Selected components were employed as input for t-SNE function in sklearn.manifold python package (Loo et al., 2019), using the following parameters: perplexity 30, number of iterations 7000 and learning rate 700. Plots were obtained by ggplot2 R package.

Cell cluster analysis by Seurat

The datasets from GSE107122 series were used and, in detail, the "Combined_Only_Cortical_Cells" matrixes were analysed for each time point of development. For the single cell RNAseq data clustering the standard workflow of the R package Seurat 4.0 was followed. No cleaning was needed and the detection of nearest neighbours was performed using respectively 10, 15, 15 and 20 principal components for the E11.5, E13.5, E15.5 and E17.5 datasets. For all samples, the original Louvain algorithm was used for the clustering with a resolution of 1. The same number of principal components were used to perform the Uniform Manifold Approximation and Projection (UMAP) dimensional reduction.

Exon-Intron split analysis (EISA)

EISA on mouse cortex transcriptomes of cortical progenitor cells at E11.5, E13.5, E15.5 and E17.5 (Chui et al., 2020) was performed as previously described in (Gaidatzis et al., 2015; La Manno et al., 2018), with modifications. Mapping of datasets to mouse genome annotation GRCm38.98 was carried out as described in https://www.kallistobus.tools/velocity_index_tutorial.html (La Manno et al., 2018). Briefly, by using USCS table

browser we obtained intron BED file, cDNA file and genome fasta files. A mouse GTF file was obtained from the Ensembl.t2g utility and used to map transcripts to gene map (https://github.com/sbooeshaghi/tools/releases/tag/t2g_v0.24.0). Intron BED file was converted to fasta format by bedtools (v2.25; https://github.com/arq5x/bedtools2/releases). Association of intron and exon identifiers was performed modifying the fasta file headers as described in (https://www.kallistobus.tools/velocity_index_tutorial.html). An index was eventually produced by Salmon (version 1.1.0) (Patro et al., 2017) using the modified fasta files. Read pseudo-counts obtained by Salmon were normalized as reads per million (RPM). Log_2 (CPM) expression levels (exonic and intronic) were calculated and the exons/introns ratio was defined as the difference between log_2 exonic pseudo-counts and log_2 intronic pseudo-counts for each experimental condition.

In Utero Electroporation (IUE)
All animal procedures were approved by the internal Ethical Committee for Animal Experimentation (OPBA) of the Ospedale Policlinico San Martino and by the Italian Ministry of Health according to the Italian law D. lgs 26/2014 and the European Directive 2010/63/EU of the European Parliament. In all the experiments, the C57BL/6J strain from Jackson Laboratory was used.
In utero intraventricular electroporation was performed on E13 mouse embryos following laparotomy of deeply anesthetized pregnant females. Embryos were injected within the telencephalic ventricles with approximately 2 µL (2 µg) of pEGFP-C1 (Clontech; control) or pEGFP-C1 bearing normal *Satb2* 3' UTR, which were immediately electroporated at 35V with 4 pulses lasting 50 ms and spaced by 950 ms with a NEPA21 (NepaGene, Chiba, Japan) electroporator. Brains were dissected 7 days after electroporation and fixed overnight at 4°C in 4% paraformaldehyde in PBS. Brains were then cryoprotected overnight in 20% sucrose, embedded in Tissue Teck O.C.T. compound (4583, Sakura) and sectioned with a Leica CM3050 S cryostat at 12 µm thickness.

RNA Immunoprecipitation
Cross-linking Immunoprecipitation (CLIP) was carried out to enrich AGO-interacting RNA. Cells were differentiated into cortical neurons until DIV12 or DIV18. Adherent cells were rinsed twice in PBS, cross-linked 150 mJ/cm^2 at 254 nm wave length, scraped, spun down 10 seconds at top speed and lysed on ice for 10 minutes in 1 mL of fresh lysis buffer (Tris-HCl 25mM pH 8.0, NaCl 150mM, $MgCl_2$ 2mM, 0.5% NP-40, DTT 5mM) with protease inhibitors (1 tablet/10 mL lysis buffer of EDTA-free Complete Protease Inhibitor Cocktail Tablets, 11697498001, Sigma Aldrich) and RNasin (250 U/mL final, N2115, Promega). Cell lysate was centrifuged at 10000 rpm at 4ºC for 10 minutes and the supernatant was kept at 4ºC for later procedure.
In the meantime, protein A Dynabeads (10001D, ThermoFisher Scientific) were rinsed 3 times with PBS/0.5% NP40 and incubated with 5 µg rabbit monoclonal Anti-argonaute-2 antibody EPR10411 (ab186733, Abcam), or anti-GFP antibody A-6455 (A-6455, ThermoFisher Scientific) in PBD/0.5% NP40 for 1 hour. After the initial binding, antibody-protein A beads were blocked with 0.5 mg/mL yeast RNA (10 µg/µL, 10109223001, Sigma Aldrich) and 1 mg/mL BSA (20 mg/mL, A3294-100G, Sigma Aldrich) for an additional 30 minutes; beads were then washed twice in PBS/0.5% NP40 to remove the unbound IgGs and then twice in lysis buffer. The beads were resuspended in 100 µL of lysis buffer.
The lysate was subjected to preclearance by incubation with pre-blocked Protein A beads at 4ºC for 60 minutes (100 µL of total lysate after pre-clearance, but before co-IP, was separated for total RNA – input – analysis). The remaining lysates proceeded to co-IP with anti-Ago-Protein A beads at 4ºC for 90 minutes. After incubation, beads were washed three times with lysis buffer, twice with lysis buffer high-salt content (Tris-HCl pH 8.0 25mM, NaCl 0.9 M, MgCl2 1mM, NP-40 1%, DTT 5Mm) and, again, once with lysis buffer. After washes, beads were incubated with 100 µL of SDS 0.1% and Proteinase K (0.5 mg/mL, P8107S, NEB) for 15 minutes at 55ºC.
RNAs that co-immunoprecipitated with anti-AGO or anti-GFP antibodies were extracted adding 700 µL Qiazol (79306, Qiagen) and 140 µL chlorophorm according to manual and then purified using Nucleospin RNA XS purification system (740902.50, Macherey-Nagel) following manufacturer's instructions.

Semiquantitative Real-Time PCR
RNA quantity and quality was measured using NanodropTM Lite UV Visible Spectrophotometer (ThermoFisher Scientific) followed by reverse transcriptase protocol. For each sample, 100 ng of total RNA were reverse transcribed. Reverse Transcriptase Core kit (RT-RTCK-03, Eurogentec) was employed for cDNA synthesis. Primers for amplification were 5'CATGAGCCCTGGTCTTCTCT3' (*Satb2* forward) and 5'AACTGCTCTGGGAATGGGTG3' (*Satb2* reverse). Amplified cDNA was quantified using Sensi Fast SYBR Green (BIO-98050, Bioline) on Rotor-Gene 6000 (Corbett). Amplification take-off values were evaluated using the built-in Rotor-Gene 6000 "relative quantification analysis" function and relative expression was calculated with the 2-ΔCt method.

Small RNA-Seq

Total RNA was extracted with miRNeasy Mini Kit (217004, QIAGEN). Small-RNA libraries were prepared using TruSeq Small RNA Sample Preparation Kit (RS-200-0012/24/36, Illumina) following the manufacturer's instructions starting from 1μg of total RNA per sample. Libraries were multiplexed, loaded into a V3 flow cell and sequenced in a single-reads mode (50 bp) on a MiSeq sequencer (Illumina), obtaining ~4 million reads per samples. Raw sequences were demultiplexed to FASTQ format using CASAVA v.1.8 (Illumina). Quality control checks were performed with the FastQC algorithm. Adapters were trimmed from the primary reads using Cutadapt v1.2.1 (Martin, 2011). Remaining reads, with a length of between 17bp and 35bp, were clustered by unique hits and mapped to pre-miRNA sequences (miRBase release 21) (Kozomara and Griffiths-Jones, 2014) with the miRExpress tool v 2.1.3 5 (Wang et al., 2009). Read counts were CPM normalized for comparative analyses. PCA was carried out by PCA.GENES R package.

miR-CATCH

miR-CATCH analysis (version 2.0) (Marranci et al., 2019; Vencken et al., 2015) was performed essentially as described, with minor modifications. Three biological replicas for each time of *in vitro* differentiation were included in the study. Mouse cells (>10^7/sample) were harvested at DIV12 and DIV18 of the cortical differentiation protocol by trypsinisation, washed with PBS and fixed with 1% formaldehyde for 10 minutes at room temperature. The reaction was quenched with 1.25M glycine for 5 minutes at room temperature and cells were centrifuged at 200g for 5 minutes at 4°C. The pellet was resuspended in ice cold PBS (50 mL) and centrifuged twice at the same conditions as previously. Cells were then resuspended in 1mL Lysis Buffer (50mM Tris-HCl pH 7.0, 5mM EDTA, 1% SDS) plus supplements: 1mM Phenylmethanesulfonyl fluoride (PMSF, P7626, Sigma Aldrich), 1X Protease Inhibitor Cocktail (P8340, Sigma Aldrich) and 80U/mL RNAsin (N2115, Promega); all the components were added freshly before use. Cells were sonicated in ice-cold Lysis Buffer with a Soniprep 150 ultrasonic disintegrator (MSS150.CX3.1, MSE) for 12 rounds at 70% amplitude for 30 seconds pulses with 45 second cool down pauses in between. Sonicated lysates were pooled in order to have a minimum of 1 mL to be hybridized with two probe pools, each containing 12 antisense biotin-labeled oligonucleotides, ODD or EVEN, as indicated in Table S1.

PROBE #	PROBE (5'-> 3')	PROBE POSITION *	PERCENT GC
**1**	aaagtccttggacccatcta	24	45.0%
**2**	tctgagcttactcagtctat	154	40.0%
**3**	cttccataagttggcaggaa	273	45.0%
**4**	attgtaaagttctctgtccc	408	40.0%
**5**	agtgactcactgtgaagtgg	492	50.0%
**6**	attacccattaaaagctgcc	627	40.0%
**7**	ctctggaggaattggtctta	753	45.0%
**8**	ctcgatacagtgctggcatg	835	55.0%
**9**	ggtccaacgtcaaaacgtca	928	50.0%
**10**	gaaggaaagggtaacaccct	1048	50.0%
**11**	tctaaccgggcagaaacttc	1231	50.0%
**12**	tctggctaaagtgaagggga	1336	50.0%
**13**	tcacttactttattgcctgg	1441	40.0%
**14**	tggcattagttctgctttac	1537	40.0%
**15**	ctggaaggtaatgctactgt	1635	45.0%
**16**	tgctgagtggccatctcaag	1724	55.0%
**17**	tgtattgcaacgtgtcttct	1976	40.0%
**18**	gctcatgtcaagggtaactg	2078	50.0%
**19**	ggagatcaggaagcagcaac	2196	55.0%
**20**	agagtgacttcagcaacagc	2245	50.0%
**21**	gatgccatcgatcgatgaac	2310	50.0%
**22**	aaatgcccacagattcactt	2436	40.0%
**23**	ctttgtcaagaggcactaca	2557	45.0%
**24**	acagcctaacaatgcacata	2739	40.0%

Table S1. miR-CATCH probes

Dynabeads MyOne Streptavidin C1 (65001, ThermoFisher Scientific) were washed (30 µL for each experiment) three times with 1 mL unsupplemented Lysis Buffer and resuspended in 30 µl complete Lysis Buffer. The beads were added to 1 mL lysate in a 1.5 mL tube and kept on rotation in a 37°C hybridization oven for 30 minutes. Then, the lysates were cleared from beads twice using a magnetic stand and transferred to a 5 mL round-bottom tube where 2 mL of supplemented Hybridization Buffer (750mM NaCl, 1% SDS, 50mM Tris-HCl pH 7.0, 1mM EDTA and 15% formamide plus supplements: 1mM PMSF, 1X protease inhibitor cocktail and 80U/mL RNAsin that were added fresh before use) were added. At this point, a total amount of 100pmol probes (capture ODD/EVEN or scrambled control probes, 1 µl from a 100 µl pool previously mixed) were added to each lysate and put again in rotation in a 37°C hybridization oven for 4 hours. While the probes were incubating with the lysate, 200 µL of beads were washed three times with unsupplemented Lysis Buffer and resuspended in 200 µL supplemented Lysis Buffer. 100 µL of beads were added to the lysate plus probes sample and rotated in the hybridization oven for an additional 30 minutes at 37°C. After this, beads were pelleted using the magnetic support and resuspended in 1 mL of Wash Buffer (2X SSC Buffer, 0.5% SDS and 1mM PMSF added fresh) pre-warmed at 37°C. Five washes of 5 minutes each using hybridization oven at 37°C were performed in rotation with the Wash Buffer. At the last wash, the beads were spun down and all the wash buffer was removed. Beads

were then resuspended in 185 µL Proteinase K buffer (100mM NaCl, 10mM Tris-HCl pH 7.0, 1mM EDTA, 0.5% SDS), added with 15 µL of 20 mg/mL Proteinase K and then incubated at 45°C for 1 hour under constant and vigorous agitation followed by 10 minutes incubation at 95°C. Finally, 1 mL Qiazol was added directly to the beads, vortexed and incubated for 10 minutes at room temperature. The RNA extraction was performed using Nucleospin RNA XS purification system (740902.50, Macherey-Nagel).
The RNA eluted from the ODD and EVEN samples were used to prepare cDNA libraries with the TruSeq Small RNA kit (RS-200-0012/24/36, Illumina), as per the manufacturer's suggestions. cDNA libraries were multiplexed, loaded into a V3 flow cell and sequenced in a single-reads mode (50 bp) on a MiSeq sequencer (Illumina), obtaining ~4 million reads per samples. Read counts reported in Data S3 were obtained as described in the miR-seq section method. To evaluate the enrichment of miRNA binding to *Satb2* 3'UTR, at each time of analysis (DIV12 or DIV18) *Satb2*-captured RNA from three independent experiments (DIV12: 3 EVEN and 2 ODD biological replicas; DIV18: 2 EVEN and 2 ODD biological replicas) and total RNA (DIV12 and DIV18, n=3) were compared. miRNA reads were normalized as CPM. Aiming to discover miRNAs with high biological relevance, those in the highest quartile of expression were considered for the analysis. The enrichment of miRNA binding to *Satb2* 3'UTR was evaluated as $\log_2$ captured/input fold change. The non-parametric NOISeqBIO statistical test of NOISeq R-package was applied with a probability >0.9 (Tarazona et al., 2015). In Data S3, log2FC indicates the fold change between captured and input miRNAs. Means refer to average CPM of captured (C) and input (control) miRNAs. Prob is the probability of differential expression calculated by NOIseqbio (Tarazona et al., 2015). Theta indicates differential expression statistics.

In situ hybridization

miRNA *in situ* hybridization (ISH) was performed using LNA-modified oligonucletide probes (Exiqon), according to the manufacturer protocol, with minor modifications. Cryosections were collected on slides (J1800AMNZT, Thermo Scientific) and postfixed 15 minutes with 4% paraformaldehyde (PFA) in PBS. Sections were treated with 10ng/µL proteinase K (15 minutes), washed with 2 mg/mL glycine (2x5 minutes), PBS (2x5 minutes), and postfixed 15 minutes with 4% PFA. Sections were then pre-hybridized (50 minutes) in hybridization solution containing: with 50% formamide, 5X sodium saline citrate buffer (SSC) (pH 6), 1% sodium dodecyl sulfate (SDS), 50 g/mL heparin (9041-08-1, ThermoFisher Scientific) and 500 g/mL yeast RNA (10109223001, Sigma Aldrich). Hybridization with the digoxigenin-labeled probes was performed overnight at a temperature approximately 21°C lower than the melting temperature of the probe. miRNA probes (miRNA Detection Probes, 339111, Exiqon) to mmu-miR-541-5p, and control probe with scrambled sequence, were employed. Washes were carried out in 50% formamide, 2XSSC at the hybridization temperature (1x30 minutes) and 1XSSC (2x15 minutes). Sections were blocked 30 minutes in MABT (1% BSA, A3294-100G, Sigma Aldrich; 150mM NaCl; 0.1% Tween 20, pH7.5) containing 10% sheep serum (S2263, Sigma Aldrich) and incubated with alkaline phosphatase (AP)-labeled anti-digoxigenin antibody (1:2000; 11093274910, Sigma Aldrich)) in MABT and 1% BSA, overnight at 4°C. Sections were washed 5x5 minutes in MABT and 3x5 minutes in NMNT (100mM NaCl, 100mM TrisHCl pH 9.5, 50mM MgCl, 0.1% Tween-20, 2mM Tetramisole (L9756-5G, Sigma Aldrich: 500 mg/L). Sections were eventually stained with BM-Purple AP-substrate (L9756-5G, Sigma Aldrich) at RT 0.5- 2 hours, then blocked by washes with PBS and counter-stained with anti-SATB2 antibody.

RNA-seq

RNA-seq libraries were prepared with the SMART-Seq® HT PLUS Kit (Takara) following manufacturer's instructions and sequenced on a NovaSeq instrument (Illumina), obtaining between 20-35M reads per sample.
Transcripts were quantified using Salmon (REF:10.1038/nmeth.4197) in mapping-based mode (with its default "--validateMappings" flag) taking as a reference a decoy-aware version of the Ensembl mouse transcriptome (mm10; refgenomes.databio.org).
RNA-seq analysis was performed using the R package NOISeq. Raw counts were normalized with the Trimmed Mean of M values (TMM) method. Low-count filtering was performed using the CPM method, with cpm=4 as threshold. PCA exploration was carried out to confirm that the experimental samples were clustered according to the experimental design (see Figure S5). Differential expression was calculated by the NOISeqBIO method and a significance threshold of q=0.95 was applied.

MiRNA-mRNA interaction prediction and GO enrichment

miRNA-mRNA *in silico* affinity was predicted as described (Enright et al., 2003), using score >120, energy < -18 kd as thresholds. 3'UTR sequences were obtained from Ensembl resources (Hunt et al., 2018), using Cran Biomart package. MiRNA sequences were obtained from miRBase database (v.22) (Kozomara et al., 2019). Enriched GO terms were obtained using two unranked lists of genes (target versus background) as described (Eden et al., 2009). Analysis results were visualized using Cran ggplot2 packages.
