# Supplementary material for: A eutherian-specific microRNA controls the translation of Satb2 in a model of cortical differentiation
Source: Stem Cell Reports. 2021 May 20;16(6):1496–509. doi: 10.1016/j.stemcr.2021.04.020 (PMC8190598; doi:10.1016/j.stemcr.2021.04.020)
Supplement: Document S2. Article plus supplemental information [file mmc8.pdf]

Stem Cell Reports
Article
OPEN ACCESS

A eutherian-specific microRNA controls the translation of Satb2 in a model of cortical differentiation

Manuella Martins,[1,2] Silvia Galfrè,[1,3] Marco Terrigno,[1,2] Luca Pandolfini,[1] Irene Appolloni,[4,5] Keagan Dunville,[1,2] Andrea Marranci,[6,7] Milena Rizzo,[6] Alberto Mercatanti,[6] Laura Poliseno,[6,7] Francesco Morandin,[8] Marco Pietrosanto,[3] Manuela Helmer-Citterich,[3] Paolo Malatesta,[4,5] Robert Vignali,[9,10] and Federico Cremisi[1,2,10,*]

[1]Scuola Normale, Pisa, Italy
[2]Istituto di Biofisica CNR, Pisa, Italy
[3]Dipartimento di Biologia, Università Roma Tor Vergata, Roma, Italy
[4]Dipartimento di Medicina Sperimentale, Università di Genova, Genova, Italy
[5]Ospedale Policlinico San Martino, IRCCS per l'Oncologia, Genova, Italy
[6]Istituto di Fisiologia Clinica CNR, Pisa, Italy
[7]Oncogenomics Unit, Core Research Laboratory, ISPRO, Pisa, Italy
[8]Dipartimento di Scienze Matematiche, Fisiche e Informatiche, Università di Parma, Parma, Italy
[9]Dipartimento di Biologia, Università di Pisa, Pisa, Italy
[10]These authors contributed equally
*Correspondence: federico.cremisi@sns.it
https://doi.org/10.1016/j.stemcr.2021.04.020

SUMMARY

Cerebral cortical development is controlled by key transcription factors that specify the neuronal identities in the different layers. The mechanisms controlling their expression in distinct cells are only partially known. We investigated the expression and stability of *Tbr1*, *Bcl11b*, *Fezf2*, *Satb2*, and *Cux1* mRNAs in single developing mouse cortical cells. We observe that *Satb2* mRNA appears much earlier than its protein and in a set of cells broader than expected, suggesting an initial inhibition of its translation, subsequently released during development. Mechanistically, *Satb2* 3′UTR modulates protein translation of GFP reporters during mouse corticogenesis. We select miR-541, a eutherian-specific miRNA, and miR-92a/b as the best candidates responsible for SATB2 inhibition, being strongly expressed in early and reduced in late progenitor cells. Their inactivation triggers robust and premature SATB2 translation in both mouse and human cortical cells. Our findings indicate RNA interference as a major mechanism in timing cortical cell identities.

INTRODUCTION

The mammalian neocortex consists of six cell layers (I–VI) generated by radial migration of neuroblasts following an inside-out mechanism (Greig et al., 2013). Glutamatergic projection neurons are formed after the generation of layer I neurons in two main neurogenetic waves: deep projection neurons (DPNs) of layers V–VI are generated first, followed by superficial projection neurons (SPNs) of the supra-granular layers II–III (Figure 1A). Generation of layer IV neurons follows the generation of DPNs and precedes SPNs formation. Proper regulation of this developmental process is crucial and its impairment results in various disorders such as brain malformations or psychiatric diseases (Sun and Hevner, 2014). The capability to generate distinct classes of neurons depends on the progenitor cell (PC) cycle state and neuron birth date (McConnell and Kaznowski, 1991). Epigenetic birthmarks may regulate the ability of PCs to establish neuron identity in the first hour following the last cell division (Telley et al., 2019). After this, the expression of a few cell identity transcription factors (CITFs) is necessary to impart distinct cell fates, with TBR1, BCL11B, FEZF2, SATB2, and CUX1 playing an important role among them (Alcamo et al., 2008; Cubelos et al., 2010; Hevner et al, 2001, 2003; Leone et al., 2015; Srinivasan et al., 2012). These factors may initially establish early mutual activating or repressive interactions; beyond this early phase, depending on the cell context and the timing of corticogenesis, some of these interactions may change and combinatorial action may ensue to refine terminal cell phenotype (Alcamo et al., 2008; Britanova et al., 2008; Chen et al., 2008; Harb et al., 2016; Jaitner et al., 2016; McKenna et al., 2015). A precise timing of expression of these and other factors is required to ensure appropriate differentiation of the neocortex. The exact mechanisms dictating the timely expression of CITFs in one given PC and its progeny is still under scrutiny.

The evolution of the mammalian cortex is characterized by the progressive thickening of the supra-granular cell layer(s) (Dehay et al., 2015; Dehay and Kennedy, 2007). A sudden evolutionary change during mammalian cortex evolution may be the heterochronic appearance of the cortical transcription factor SATB2 with respect to the corresponding mRNA. Indeed, it was recently shown that SATB2 protein expression is delayed in eutherians compared with metatherians, and such delay seems responsible for the development of the inter-hemispheric callosal connections generated from the supra-granular

This is an open access article under the CC BY license (http://creativecommons.org/licenses/by/4.0/).

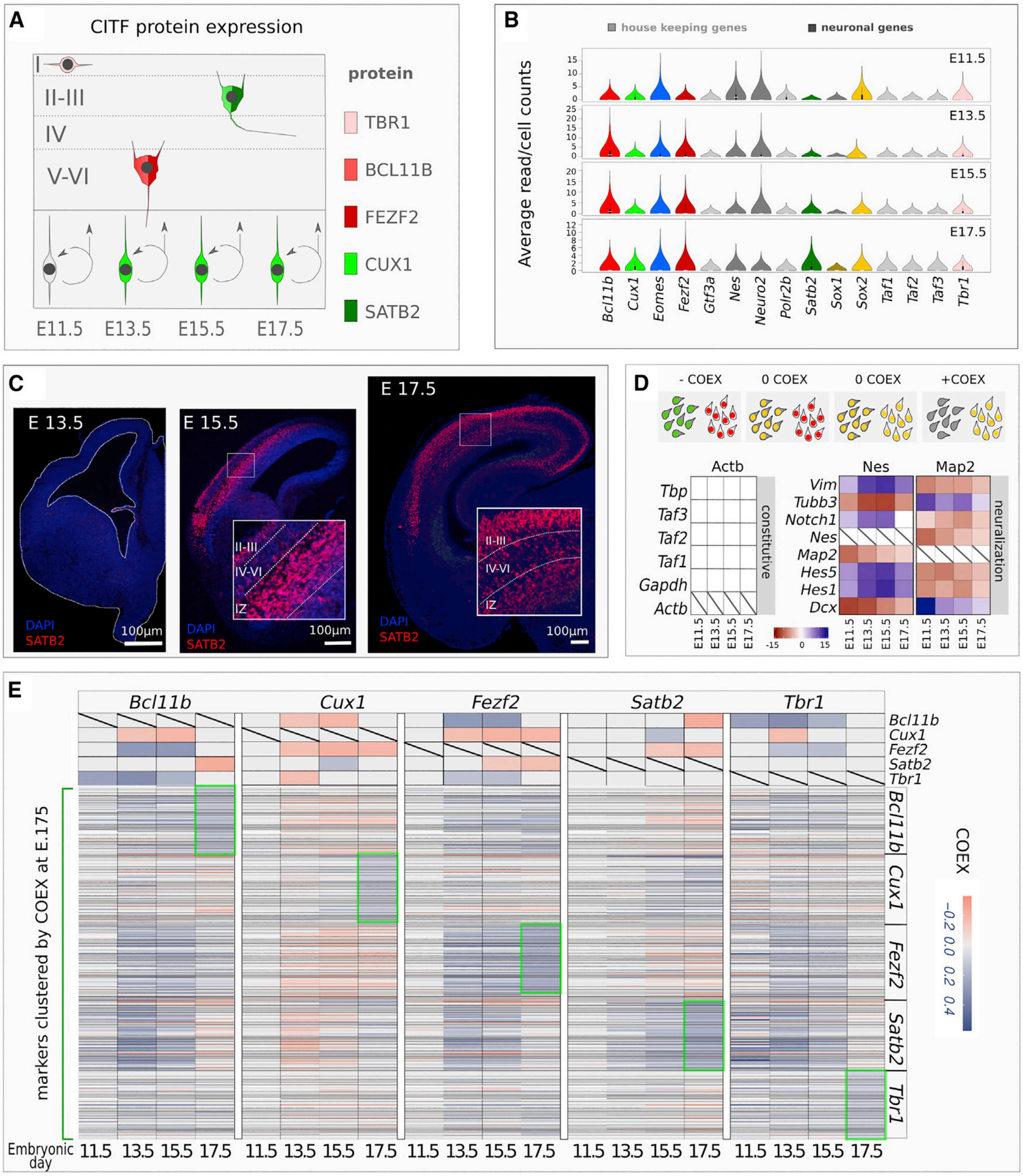

Figure 1. CITF expression analysis

(A) Simplified outline of cortical layering. Layers are labeled by Roman numerals.

(B) Violin plots show average raw counts/cell of indicated genes. Constitutive genes are in light gray.

(C) Coronal sections of mouse embryonic brain showing SATB2 immunodetection at different embryonic (E) developmental times of corticogenesis. Roman numerals indicate cortical layers. IZ, intermediate zone.

(D) Top schematic shows COTAN COEX relation to the pattern of expression of two genes (red and green) in single cell. Bottom shows COEX values of pairs of constitutive genes (left matrix) or neural differentiation markers (right matrix) at the different developmental times shown in labels.

(E) COTAN COEX values of CITFs and of their most closely associated genes at E17.5. The top side of the matrix shows the COTAN COEX relation between pairs of CITFs. The bottom part of the matrix reports COTAN COEX values between distinct CITFs and the genes that are more highly co-expressed with each of them at E17.5 (green boxes).

cells in eutherians (Paolino et al., 2020). After its evolutionary appearance, the continuous expansion of the corpus callosum (CC), and of the supra-granular cell layer it stems from, represents the distinguishing feature of the placental neocortex, including that of higher primates. Notably, in higher primates, SATB2 protein appearance is delayed over an extended period, possibly crucial for supra-granular cell layer expansion (Otani et al., 2016). In this aspect, the control of developmental timing of SATB2 during cortical neurogenesis may be of crucial importance. In this paper, we have first investigated the differential stability of mRNAs for key CITFs involved in mammalian corticogenesis, namely *Bcl11b*, *Cux1*, *Tbr1*, *Fezf2*, and *Satb2*, by exon/intron (E/I) stability analysis (EISA) (Gaidatzis et al., 2015). We find that among them only *Satb2* mRNA shows an increase in E/I ratio due to an improved stability and rate of its transcription. We then show that a post-transcriptional control is played by microRNAs (miRNAs) acting on *Satb2* 3′UTR. We isolated miRNAs that bind to this region and focus on miR-541, a new, eutherian-specific miRNA; we show that miR-541 delays, both *in vivo* and *in vitro*, SATB2 protein production with respect to *Satb2* mRNA transcription. We discuss the potential implications of miR-541 action in the scenario of cortical evolution.

RESULTS

Satb2 is co-transcribed with other CITFs in early cortical cells before its translation

Since DPNs and SPNs are sequentially generated in an inside-out fashion from embryonic day 11.5 (E11.5) to E17.5 in mouse (Figure 1A), we expect that the mRNA of CITFs is regulated in selected PCs in this time window. We tested this assumption by re-analyzing single-cell RNA sequencing (scRNA-seq) datasets of mouse cortex at E11.5, E13.5, E15.5, and E17.5, generated by droplet sequencing from dissociated whole embryonic cortices (average depth more than 50,000 reads/cell; transcriptomes from 2,000 cells at E11.5, E13.5, and E17.5; 5,000 cells at E15.5) (Yuzwa et al., 2017). We compared the average expression levels of the 5 CITFs (raw counts/cell) with those of constitutively expressed transcription factors (Figure 1B). The mRNA expression levels of all 5 CITFs are comparable with those of constitutive transcription factors since E11.5, indicating that these five mRNAs could have a biological relevance since very early stages of corticogenesis. However, we did not detect SATB2 translation before E15.5 (Figure 1C), although a dynamic pattern of *Satb2* transcriptional activation in the dorsal telencephalon starts from E11.5 (Tashiro et al., 2011). Although a minority of SATB2-positive cells were reported at E13.5 (Alcamo et al., 2008; Britanova et al., 2008), a reliable onset of SATB2 protein expression was not described earlier than E14 (Paolino et al., 2020), suggesting a post-transcriptional regulation of *Satb2* mRNA.

To get insights into the pattern of CITF transcriptional activation in specific cell subsets, we analyzed CITF co-expression in single cells by co-expression table analysis (COTAN) (Galfrè et al., 2020). COTAN can assess the co-expression of gene pairs in a cell and, by extending this analysis to all gene pairs in the whole transcriptome, can indicate the tendency of a gene to be constitutively expressed or expressed in a subset of differentiating/differentiated cells. Positive co-expression index (COEX) denotes co-expression of two genes, while negative COEX indicates disjoint expression; COEX near zero is expected if one or both are constitutive genes (Figure 1D, top) or when the statistical power is too low. Accordingly, our analysis gives COEX values close to zero for constitutive mRNA pairs (Figure 1D, left; Data S1). Conversely, high co-expression (positive COEX) is found for mRNA pairs of known molecular markers of neural PCs (*Nestin*, *Vimentin*, *Notch1*, *Hes1-5*) or post-mitotic cells and differentiating neurons (*Dcx*, *Tubb3*, *Map2*). Finally, negative COEX (disjoint expression) is detected between mRNA pairs of these two groups at all developmental stages (Figure 1D). All CITFs show reciprocal mRNA co-expression patterns consistent with their known protein expression pattern in different cell types, except *Satb2*, whose COEX with each of the other four CITFs at E11.5 and E13.5 is comparable with that of constitutive genes (compare Figures 1A and 1E, top).

We considered the genes most highly co-expressed with each CITF gene at E17.5 (Figure 1E bottom, Figure S1). At this stage, the final pattern of co-expression of each CITF gene with co-clustered markers (green boxes in Figure 1E) differs from the patterns at earlier stages (Figure 1E). This suggests that initial CITF gene expression is not cell layer specific, but cell-specific CITF gene expression is reached toward the end of layer formation.

COTAN Gene Differentiation Index (GDI) discerns between constitutive and non-constitutive genes by globally integrating COEX values (Galfrè et al., 2020) (Figure 2A). We used GDI analysis to infer the propensity of CITFs to be expressed in restricted cell subsets during corticogenesis. Notably, the global relation between GDI and mRNA levels (Figure 2B), and the global GDI distribution (Figure 2C), are comparable in the four analyzed stages. This observation supports the use of GDI analysis to evaluate whether an mRNA species changes its pattern of cell distribution during corticogenesis, and becomes restricted to a particular cell lineage/layer. Unlike constitutive genes such as *Actb*, CITFs showed marked GDI changes during corticogenesis (Figure 2D). *Tbr1* mRNA shows a peak at E11.5, consistent

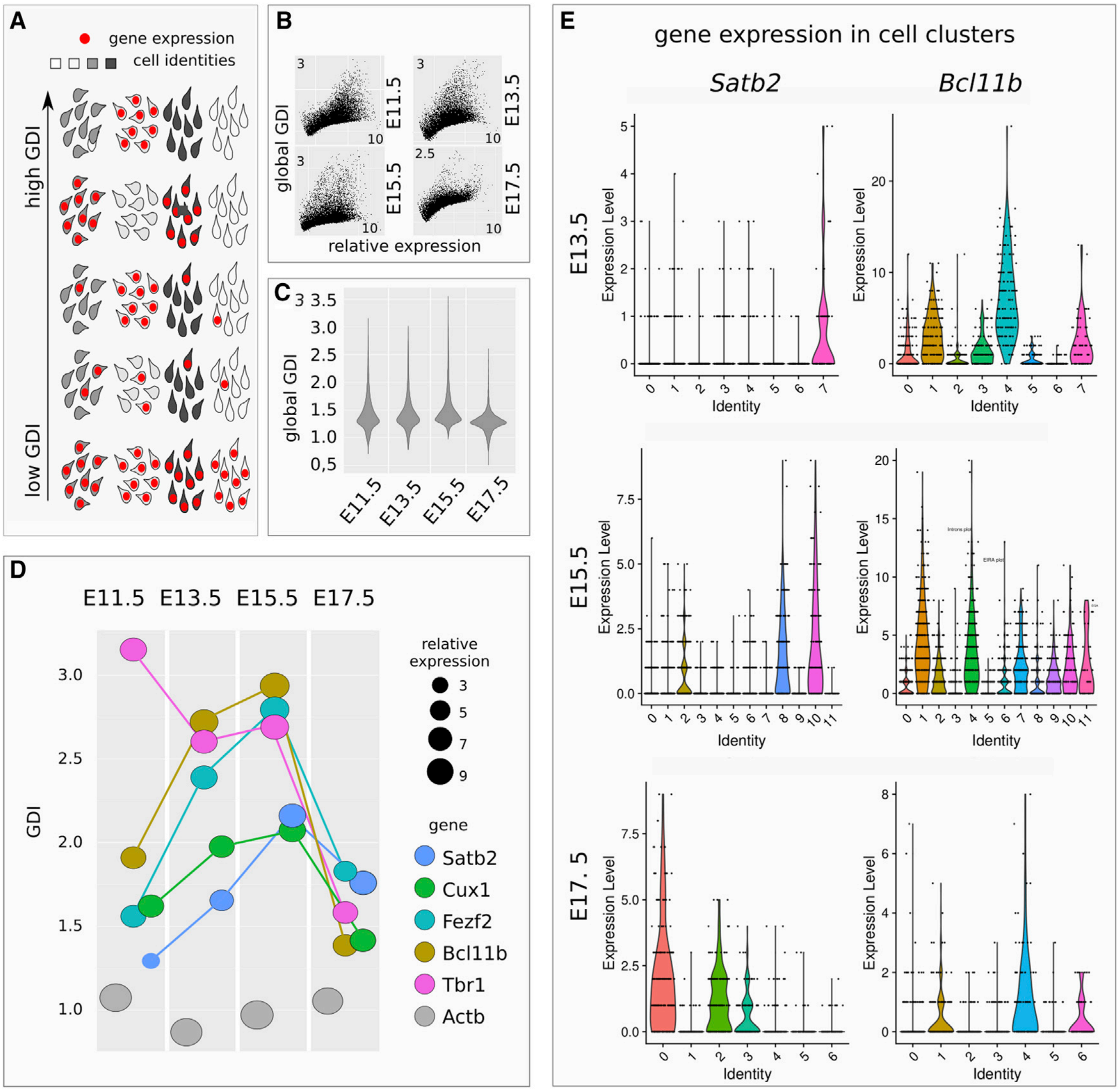

Figure 2. CITF transcription in distinct cell clusters

(A) Schematic shows how GDI can indicate the degree of gene pair co-expression in cell populations with different cell identities.

(B) Plots show GDI and gene mRNA expression levels at different developmental times.

(C) Violin plots report global GDI distribution during corticogenesis.

(D) Distinct CITFs show different GDI according to their translational onset.

(E) Clustering of DIV13.5, DIV15.5, and DIV17.5; violin plots show count distribution for the indicated gene in cell clusters. Analysis was performed by R package Seurat 4.0.

with early localized TBR1 protein expression in layer 1 neurons (Hevner et al., 2001). *Bcl11b* and *Fezf2*, followed by *Satb2* and *Cux1*, increase their GDIs until E15.5, paralleling their respective onset of protein expression (compare Figures 2D with 1A).

The drop of GDI observed at E17.5 correlates with, and might be explained by, the increased heterogeneity of the cell types co-expressing different combinations of CITF proteins at the end of corticogenesis (Lodato and Arlotta, 2015), although it may also be due to post-transcriptional CITF regulation. Notably, *Satb2* displays the lowest GDI levels among CITFs at E11.5–13.5, when its protein is not yet detectable, suggesting that post-transcriptional control accounts, at least in part, for the subsequent restricted expression of SATB2 protein in SPNs. Finally, we used a conventional scRNA-seq data clustering (Figure 2E). The lack of a cell-type restricted distribution of *Satb2* mRNA at early stages is also suggested by its partial overlap with *Bcl11b* mRNA in E13.5 and E15.5 cell clusters, compared with E17.5 clusters.

Satb2 3′UTR drives RNA-induced silencing complex-dependent translational inhibition in early cortical cells

We then took advantage of EISA (Gaidatzis et al., 2015; La Manno et al., 2018) to verify whether a time-dependent instability of *Satb2* mRNA could account for the inability to detect SATB2 protein at E13.5, when *Satb2* transcription is already robust and coincident with that of *Bcl11b*. EISA evaluates changes of stability of specific mRNAs during developmental processes, assuming that the intronic sequences are rapidly spliced and that their levels reflect the gene transcriptional rate (see schematic in Figure 3A, left). Because layer identity is assigned before neuron birth date (McConnell and Kaznowski, 1991; Telley et al., 2019), we analyzed RNA-seq datasets of PCs (Chui et al., 2020). We observed that *Satb2* E/I ratio significantly increases from E11.5 to E17.5, *Bcl11b* E/I increases from E11.5 to E13.5, and *Fezf2* E/I increases from E13.5 to E15.5, while the E/Is of the other CITFs and of *Actb* show no significant changes (Figure 3A, middle panel). Notably, *Satb2* E/I increase is paralleled by a dramatic increase of its transcription levels from E11.5 to E17.5 (Figure 3A, right), as measured by intron read abundance, making its E/I increase more relevant than that of *Bcl11b* and *Fezf2*. *Satb2* E/I fold change between E13.5 and E15.5 settles in the highest quartile of the E/I increase (Figure 3B and Data S2), suggesting high biological relevance and supporting a close relationship between the increase of *Satb2* mRNA stability and the onset of SATB2 translation. We thus focused our attention on *Satb2* post-transcriptional regulation.

We reasoned that changes in *Satb2* mRNA stability could be induced by miRNAs. Indeed, by high-throughput analysis of miRNA-mRNA interactions at single-cell level, distinct miRNAs were recently associated to functional modules involved in the control of cortical cell identities (Nowakowski et al., 2018). To gain insights on RNA interference in early corticogenesis, we employed mouse embryonic stem cells (mESCs), whose *in vitro* neural differentiation can closely reproduce the early stages of cortical development, including time-regulated expression of TBR1, BCL11B, and SATB2 protein (Bertacchi et al., 2015; Gaspard et al., 2008). In these corticalized mESCs, we measured the enrichment of *Satb2* mRNA after AGO2 immunoprecipitation. By qRT-PCR, a significant enrichment of AGO2-bound *Satb2* mRNA is detected after 12 days *in vitro* (DIV) compared with control anti-GFP immunoprecipitation, indicating a strong miRNA silencing activity in early *in vitro* corticogenesis (Figure 3C). Notably, we found no enrichment at DIV18, consistent with a significant increase of SATB2-positive cells at this time (Bertacchi et al., 2015).

The changing ability of *Satb2* mRNA to bind AGO2 during development is in line with the ability of its 3′UTR to inhibit protein translation in early, but not late, cortical cells. Indeed, at DIV12 the transfection of a GFP reporter carrying *Satb2* 3′UTR yields decreased fluorescence levels compared with control, while at DIV18 the reporter activity is not significantly affected (Figure 3D), consistent with robust SATB2 translation at this late stage (Bertacchi et al., 2015). *Satb2* 3′UTR is able to control translation also *in vivo*, as shown by *in utero* electroporation (IUE) of a GFP reporter/sponge. At stage E13.5, the ratio of SATB2-GFP double-positive cells to GFP-positive cells is significantly higher in a cortex electroporated with a 3′UTR-bearing sensor compared with a control cortex (Figure S2). These results show that *Satb2* 3′UTR can inhibit the translation of its mRNA in early-generated neurons.

MiRNAome time trajectories describe cortical development progression

We then set out to identify miRNA candidates regulating *Satb2* expression. With this aim, we sorted *Sox1*::GFP corticalized mESCs, which are enriched in PCs, and first compared their global miRNA profiles with those of non-neuralized mESCs, of post-mitotic corticalized mESCs obtained by AraC treatment, or of mouse cortex, at different developmental times (Figures 4A–4D and Data S3). MiRNAome principal component analysis (PCA) shows high consistency between miRNA profile and cell identity. MiRNAomes of non-neuralized mESCs are well separated from those of corticalized mESCs and of cortex, which instead cluster together, confirming that our *in vitro* protocol mimics a genuine cortical identity (Figure 4A). The time of *in vitro* differentiation distributes both PC (Figure 4B) and neuron (Figure 4C) miRNAomes along PC3, in agreement with the relative position of E12 and P0 cortex miRNAomes, denoting high conservation of the mechanisms accounting for the timing of layer formation in our *in vitro* conditions. Finally, PC3 distinguishes between progenitor and neuron miRNAomes (Figure 4D), indicating that these distinct cell states are maintained throughout the differentiation process.

Selected miRNAs directly bind *Satb2* 3′UTR in early cortical cells

To select miRNAs that directly interact with *Satb2* 3′UTR at DIV12 and DIV18, we employed miR-CATCH analysis, which recovers mRNA/RNA-induced silencing complex (RISC)/miRNA complex by biotin-labeled probes complementary to the target mRNA (Marranci et al., 2019; Vencken et al., 2015). Bound miRNAs were quantified through small RNA-seq, and miRNA enrichment was measured with respect to the input (total miRNAs) (Figure 5A). We found that 12 miRNAs bind to *Satb2* mRNA and are significantly enriched at DIV12; of these, miR-541 and miR-3099 are not enriched at DIV18, thus representing candidates for

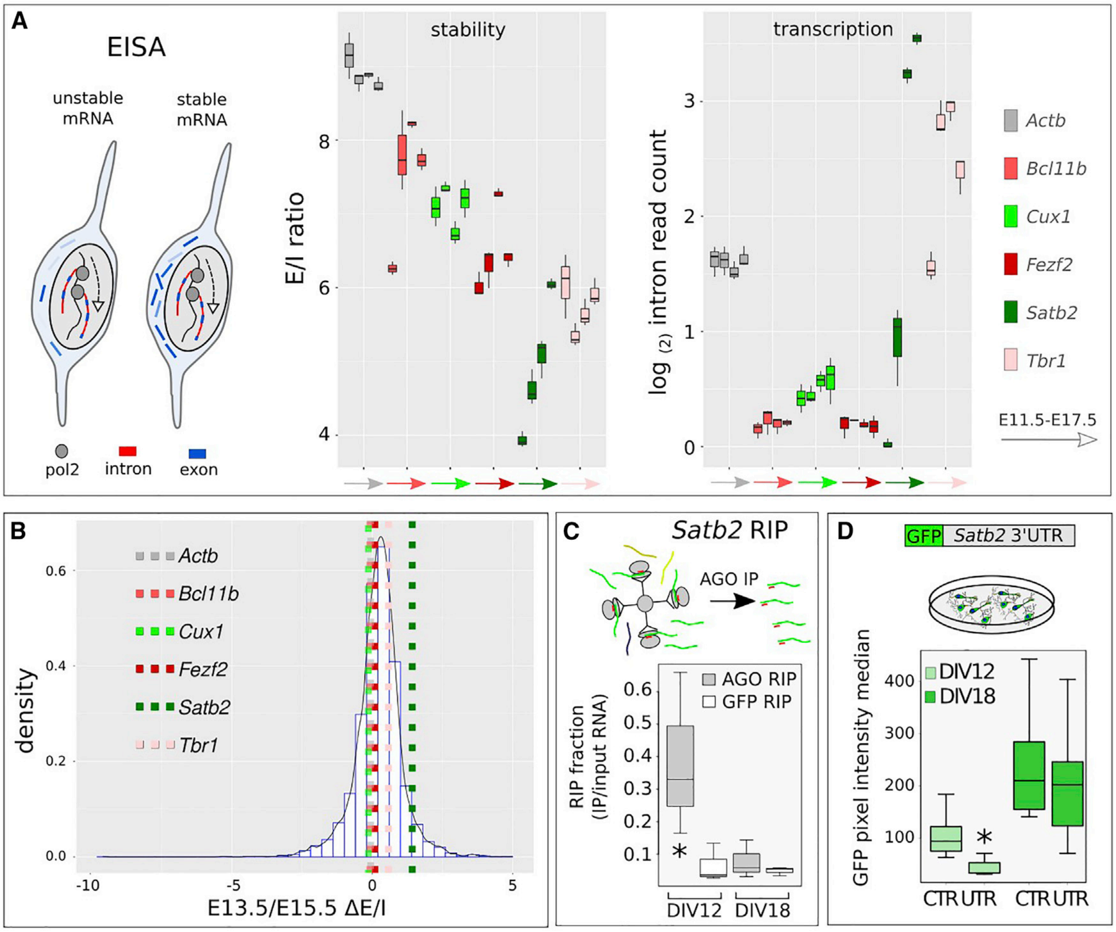

Figure 3. Cortical mRNA E/I analysis and *Satb2* translational inhibition

(A) EISA of CITF mRNAs. Outline shows different ratios of exonic and intronic sequences in relation to mRNA stability as rationale at the basis of EISA. Box plots show the ratio of E/I read counts, and intron read counts, for distinct CITFs and *Actb* (constitutive control gene) in cortical progenitors at different *in vivo* embryonic times.

(B) Density plot of E/I ratio fold change between E13.5 and E15.5.

(C) qRT-PCR evaluation of Argonaute (AGO)-interacting *Satb2* mRNA. Values on y axis report the ratio of RT-PCR-detected, immunoprecipitated *Satb2* mRNA with respect to the input (AGO RIP). GFP RIP, control immunoprecipitation with anti-GFP Ab. N = 3 independent experiments. Asterisk indicates p-value = 0.049 (Student's t test).

(D) Expression of *Satb2* 3′ UTR-bearing GFP reporter after lipofection in corticalized mESCs. N = 3 independent experiments. Cells were transfected 48 h before the time of analysis indicated in labels. Asterisk indicates p-value = 0.000061 (Wilcoxon signed rank test).

SATB2 inhibition in early, but not late, cortical cells (Figures 5B and S3 and Data S3). Because of its extremely low expression (Figure 5C), we did not further investigate miR-3099 and focused on the other miRNAs.

We analyzed the abundance of the captured miRNAs in PCs and found that only miR-92a/b and miR-541 show robust decrease between DIV12 and DIV16, when SATB2 translation is de-inhibited (Figure 5C). We thus focused our attention on these three miRNAs. miR-92 was already shown to play a major role in inhibiting EOMES (TBR2) translation and preventing early generation of basal PCs, which in mouse give rise to supra-granular neurons (Bian et al., 2013; Nowakowski et al., 2013). Conversely, miR-541 has never been involved in cortical development and belongs to an evolutionary new miRNA cluster (mir-379-mir-410 in mouse, mir-379-656 in humans), located in a large miRNA-containing gene (*Mirg*) inside the *DLK-DIO3* locus (Edwards et al., 2008; Glazov et al., 2008; Winter, 2015) (see discussion). *Mirg* orthologues have been found in all eutherians, which hold inter-hemispheric cortical connections forming the CC, but not in metatherians, prototherians, or any other vertebrates, which lack CC.

miR-541 *in vitro* pattern of expression closely matches the time-dependent inhibition of SATB2 translation and

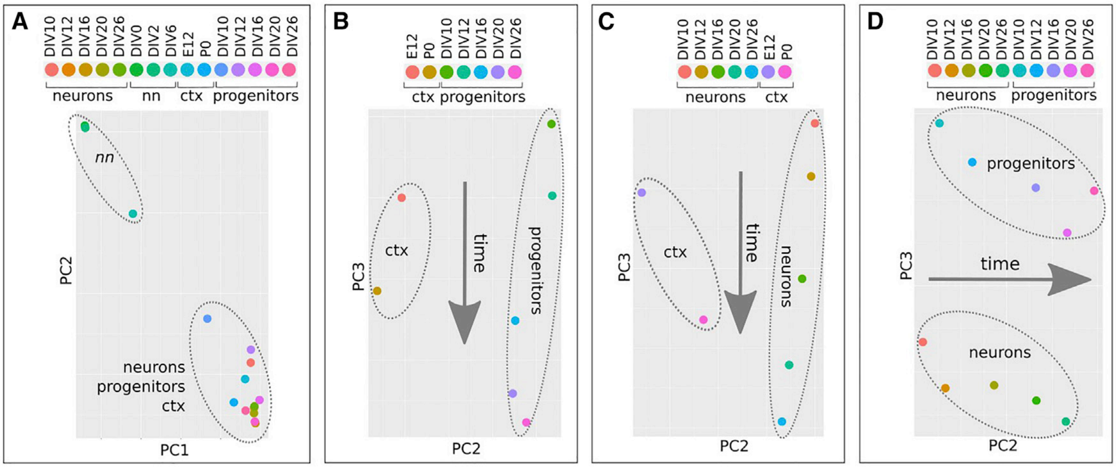

Figure 4. MiRNAome time trajectories in corticogenesis
(A–C) PCA of miRNA global profiles of non-neuralized mESCs (nn), neural progenitors (*Sox1*::GFP corticalized mESCs), post-mitotic neurons (Ara-C-treated corticalized mESCs) and mouse cortex (ctx) at different developmental times. Four different combinations of the four groups are shown.

follows a sudden downregulation between DIV12 and DIV16 (Figure 5C). In addition, at E13.5, miR-541 is widely expressed in the ventricular zone (VZ), subventricular zone (SVZ), and mantle zone (MZ), when SATB2 protein is undetectable; at E15.5, the miRNA is expressed in the cortical plate (CP), when the protein is detected in VZ, SVZ, intermediate zone (IZ), and migrating cells (Figure S4). Finally, miR-541 developmental decrease is comparable with that of the most heavily downregulated miRNAs from DIV12 to DIV16 (Figure 5D), strengthening its candidacy for the control of SATB2 inhibition in early corticogenesis.

miR-541 and miR-92a/b inhibit SATB2 translation in mouse and human early cortical cells

We then inhibited miR-541 and mir-92a/b by transfection of a complementary locked-RNA (antago-miR) in mouse ES corticalized cultures (Figure 6A). This results in a premature onset of SATB2 protein detection and in a massive increase of SATB2-positive cells compared with control-transfected cells, as found in transfection at DIV10, and in an increase of the efficiency of translation at later time points, as found in DIV12 transfection (Figures 6B top, 6C). Notably, miR-541 has no predicted binding site on *Eomes* 3′UTR; thus, its effect on SATB2 translation is unlikely mediated by increased EOMES translation and consequent induction of basal PC identity (Sessa et al., 2008), as may be the case with miR-92a/b inhibition. We observed similar effects when downregulating miR-541 and miR-92a/b in corticalized human induced pluripotent stem cells (hiPSCs) (Figures 6B bottom, 6C), denoting evolutionary conservation of this control mechanism.

We then transfected antago-miR-541 and control antago-miR at DIV12 and analyzed transcriptomes at DIV17, a time of *in vitro* development when many markers of terminal differentiation can be evaluated. We found that 489 mRNA species significantly change their expression compared with control (Figure 6D and S5 and Data S4). Gene Ontology (GO) analysis of these genes shows that many of them are significantly enriched in terms related to cell differentiation and neuron projection (Figure 6E and Data S4). Notably, many of the genes are involved in CC development and malformation (Data S4).

GO enrichment of miR-541 target genes

To infer the biological relevance of miR-92a/b and miR-541, we evaluated their degree of miRNA-mRNA target affinity using miRanda (Enright et al., 2003) as *in silico* prediction tool. miRanda was more sensitive than TargetScan (Bartel, 2009), TargetSpy (Sturm et al., 2010) and TarPmiR (Ding et al., 2016) in predicting miRNA interactions with *Satb2* 3′UTR (Figure S6A and Data S5), and predicted two sites of miR-541 interaction that were validated by the transfection of a GFP reporter carrying a mutated seed sequence in *Satb2* 3′UTR (Figures S6B–S6D). First, we analyzed the affinity of miRNA-*Satb2* 3′UTR interaction in relation to the average expression of mouse embryonic cortical miRNAs. Among the annotated miRNAs with significant affinity to *Satb2* 3′UTR (Data S6), miR-92a/b and miR-541 show high expression in cortical PCs (miR-92a/b) or high *in silico* affinity to *Satb2* 3′UTR (miR-541) (Figure 7A), in line with their high miR-CATCH enrichment (Figure 5B). miR-541 shares less than half of its targets

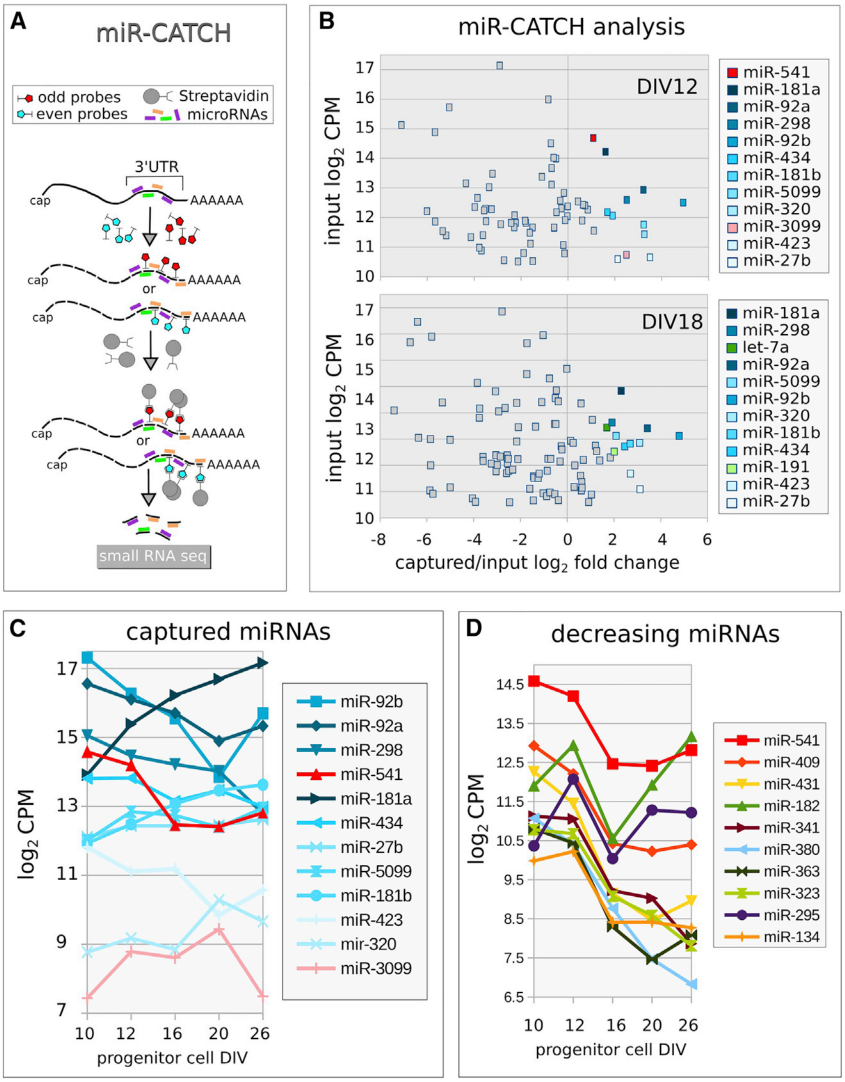

Figure 5. *Satb2* interacting miRNAs

(A) Outline of the miR-CATCH method.

(B) Enrichment of captured miRNAs (x axis) with respect to input (y axis) at the indicated time. CPM, counts per million. Color labels indicate significantly enriched miRNAs (non-parametric noiseqbio test probability >0.9) (Tarazona et al., 2012).

(C) Developmental expression patterns of *Satb2*-captured miRNAs in *Sox1*::GFP PCs.

(D) Developmental expression of miRNAs with highest monotonic developmental decrease in *Sox1*::GFP PCs.

with miR-92a/b, while miR-92a/b share most of theirs with miR-541 (Figure S6E). We then compared miR-92a/b and miR-541 targets with those of three recently described miRNAs of corticogenesis, namely let7, miR-9, and miR-128 (Shu et al., 2019). For this, we selected a subset of 395 genes associated with an embryonic cortical marker signature (Galfrè et al., 2020). Among the six miRNAs analyzed, let-7 and miR-541 showed *in silico* affinity with more than half of the signature genes (Figure 7B and Data S6), suggesting a more relevant role for them in corticogenesis. Interestingly, among the mRNAs with the highest *in silico* affinity (total score higher than 400) for the six miRNAs, only the putative targets of miR-541 showed significant enrichment in GO terms. It may be notable that terms related to neuronal projection development (axogenesis, neuron projection morphogenesis, cell projection morphogenesis, plasma membrane cell projection) (Figure 7C) are the most represented and that at least eight out of the 11 putative target genes are related to cortical neuronal layering and migration, axon guidance, and CC disturbances (Figure S7). Interestingly, all these 8 genes might be involved in basic processes controlling polarization, proliferation, and migration of late cortical PCs (Figure 7D; Figure S7 and references therein). Figure 7E compares the change of E/I read counts by EISA of seven out of the eight genes (not enough *Cdk5r* read counts were available for a significant analysis) with those of the genes of the embryonic cortical marker signature. The results indicate that all these seven genes increase their E/I read count ratios between E13.5 and E17.5 and that there is a general correlation between E/I read count increase and mir-541/mRNA affinity score, supporting a relevant role of miR-541 in their post-transcriptional control during early corticogenesis.

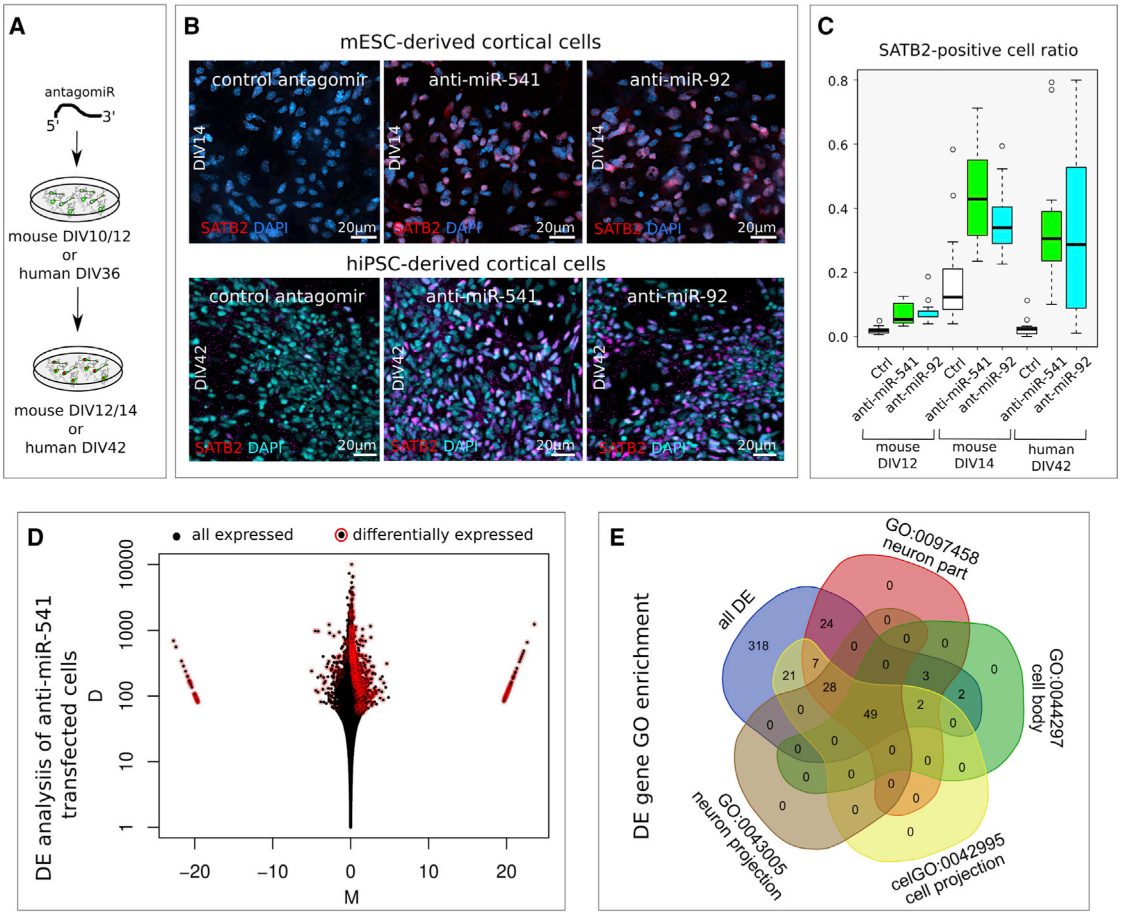

Figure 6. miR-92a/b and miR-541 function in mouse and human cortical cells

(A) Outline of the *in vitro* assay of miR-541 inhibition by locked nucleic acid (LNA)-antisense oligonucleotide lipofection in corticalized mESCs (n = 3 independent experiments) or hiPSCs (n = 3 independent experiments).

(B) Immunocytodetection shows SATB2-positive nuclei 2 days after mESC lipofection and 6 days after hiPSC lipofection, respectively.

(C) Box plots report SATB2-positive nuclei proportion. Ctr, scrambled sequence LNA lipofection. An anti-miR-92a/b LNA oligonucleotide was used to inhibit both miR-92a and miR-92b, which share the seed sequence.

(D) Mean-difference plot showing log-fold change (M) and the absolute value of the difference in mRNA expression (D) between antago-miR-541 and control antago-miR transfections (n = 3 independent experiments).

(E) Venn diagram showing the distribution of the genes differentially expressed after antago-miR transfection in the four most enriched GO terms.

DISCUSSION

Translational control exerted by RNA-binding proteins or miRNAs plays a crucial role for the appropriate time of production of key proteins that govern the potential of cortical PCs as well as the differentiative program of the post-mitotic neurons (Kosik and Nowakowski, 2018; Nowakowski et al., 2018; Shu et al., 2019; Zahr et al., 2018). For example, cortical PCs express *Brn1* and *Tle4* mRNAs, for both deep and superficial layer fates, respectively, but translation into their corresponding proteins is initially repressed and subsequently released in due time (Zahr et al., 2018). miRNAs are especially interesting as heterochronic modulators of vertebrate development (Gulman et al., 2019; Robinton et al., 2019), also in the nervous system (Chiu et al., 2014; Nowakowski et al., 2018; Zahr et al., 2019).

In this paper, we found evidence for differential stability of *Satb2* mRNA compared with other key mRNAs. SATB2 protein plays a central role in cortical neurogenesis, both in the early embryo and at later postnatal stages. Early *Satb2* inactivation leads to absence of CC, with upper layer neurons diverting their axons to subcortical targets (Alcamo et al., 2008; Britanova et al., 2008; Leone et al., 2015; McKenna et al., 2015; Srinivasan et al., 2012). Conversely, later *Satb2* inactivation leaves the CC intact,

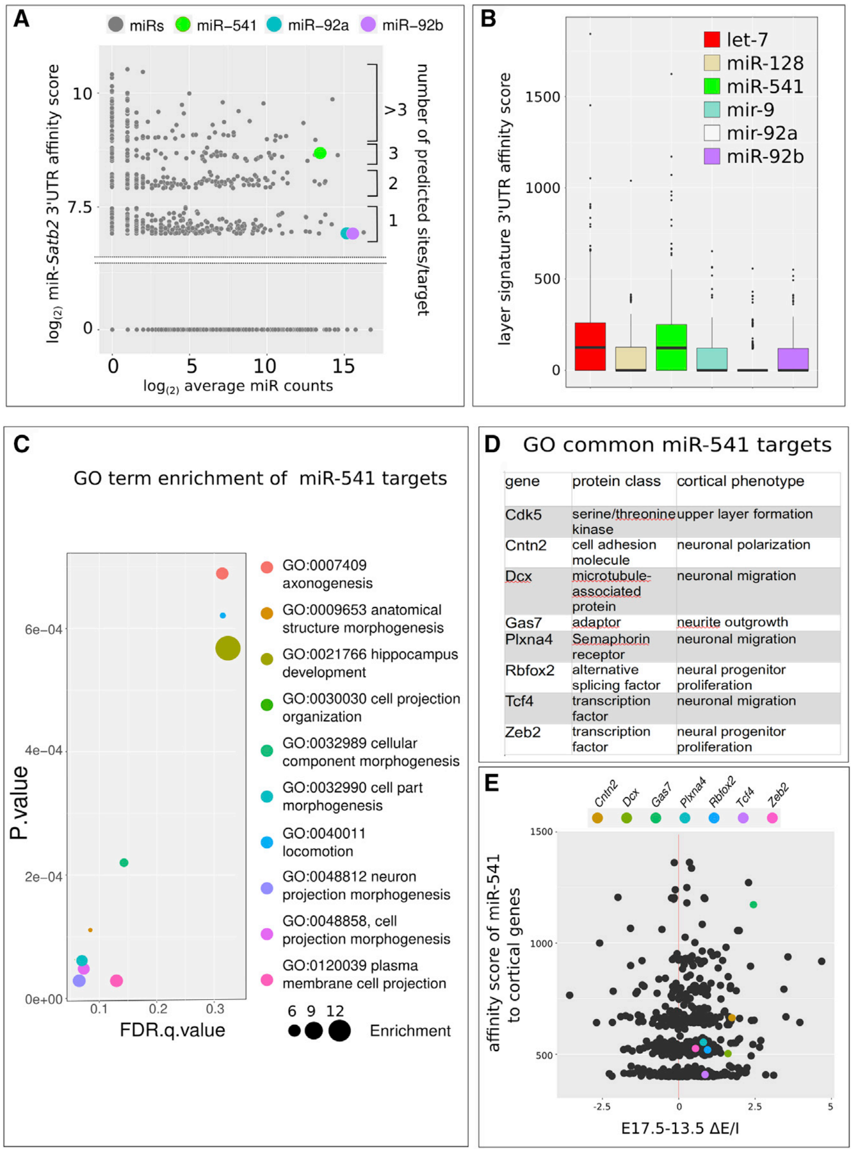

Figure 7. *In silico* analysis of miRNA/mRNA interactions

(A) *In silico* comparison of the affinity of mouse miRNAome (gray dots), miR-92a/b, and miR-541 (colored dots) with *Satb2* 3′UTR (Ensembl *Mus musculus Satb2*-201 cDNA 3′UTR), in relation to the average miRNA expression levels during corticogenesis.

(B) *In silico* affinity of cortical miRNAs to the 3′UTR of an embryonic cortical layer gene signature (395 genes) (Galfrè et al., 2020).

(C) GO enrichment of the mir-541 gene targets with high *in silico* affinity to *Satb2* 3′UTR (cumulative score higher than 400, n = 48) (Enright et al., 2003) with respect to the layer gene signature employed in (B).

(D) List of the eight genes common to all the GO terms shown in (C).

(E) Plot showing E/I read counts developmental increase (x axis) with respect to miR-541/mRNA affinity score (y axis) to genes of the embryonic cortical marker signature (Galfrè et al., 2020). Colored dots indicate genes listed in (D). Names in labels indicate the five genes with the highest E/I read count ratio increase and mir-541/mRNA affinity score.

although there are effects on plasticity and long-term memory storage (Jaitner et al., 2016). Finally, *Satb2* also plays a role in layer V subcortical projection neurons (Srinivasan et al., 2012). These data indicate that SATB2 acts in a cell context- and time-dependent multifaceted way, and that precise control of its expression may be relevant for cortical development. Significantly, Paolino et al. (2020) have shown that accurate timing of SATB2 protein appearance in mouse is crucial for axonal projection of layer II–III neurons through the CC. In fact, while SATB2 protein is readily translated from its mRNA in the dunnart marsupial model (where layer II–III axons travel through the anterior commissure and the CC is absent), in the mouse SATB2 protein appearance is delayed with respect to its mRNA expression (and axons go through the CC). Strikingly, anticipated SATB2 protein production in the mouse reroute layer II–III commissural axons toward the anterior commissure instead of the CC. Thus, a post-transcriptional control may be relevant in timing SATB2 protein appearance within the developing early placental neocortex (Paolino et al., 2020).

Our results provide evidence that *Satb2* 3′UTR contributes to this control. *Satb2* 3′UTR drives a significant translational inhibition of a GFP reporter at an early (DIV12), but not at a late (DIV18), stage of *in vitro* differentiation. Moreover, it is bound by the AGO/RISC complex in a much stronger way at an early (DIV12) than at a late (DIV18) stage, suggesting its regulation by miRNAs. We identified miR-92a/b and miR-541 as candidate miRNAs to modulate SATB2 onset of translation, on the basis of

their temporal dynamics of expression and of direct miR-CATCH biochemical selection. Significantly, antagonizing these miRNAs anticipates the appearance of SATB2-positive cells in both mESCs and hiPSCs induced to cortical differentiation *in vitro*. While the antagonism of miR-92a/b might exert this effect by anticipating the translation of EOMES (TBR2), and then the differentiation of intermediate PC progeny expressing SATB2 (Bian et al., 2013; Nowakowski et al., 2013), miR-541 has no predicted binding sites on *Eomes* mRNA. Thus, miR-541's effect on the onset of appearance of SATB2-positive neurons is directly due to its binding to *Satb2* 3′UTR. miR-541 is likely targeting a high number of genes, as suggested by the transcriptome change observed after its inhibition by antago-miR transfection and the *in silico* analysis of its targets.

Unlike miR-92a/b, let-7b, miR-128, and miR-9, and other evolutionarily conserved miRNAs involved in cortical development (Chiu et al., 2014; Nowakowski et al, 2013, 2018; Shu et al., 2019; Zahr et al., 2018), mir-541 is only present in eutherians (see below) and the only functional report shows its role in inhibiting neurite growth in PC2 cells (Zhang et al., 2011). miR-541 expression declines during corticogenesis in a temporal pattern opposite to that of SATB2 protein, and its presence in eutherians, but not in metatherians or any other vertebrates, suggests that it might be involved in the heterochronic shift of SATB2 translation between dunnart and mouse (Paolino et al., 2020). Our demonstration that miR-541 can bind *Satb2* 3′UTR and inhibit translation both *in vitro* and *in vivo* provides a molecular mechanism contributing to this heterochronic shift.

Together with about 40 miRNAs, miR-541 is encoded by *Mirg* (miRNA-containing gene), present only in eutherians inside the *Dlk1-Dio3* locus (Edwards et al., 2008; Glazov et al., 2008; Marty and Cavaillé, 2019; da Rocha et al., 2008; Winter, 2015). *Mirg* mRNA was detected in the developing early nervous system and in other organs, including the liver (Han et al., 2012). Constitutive *Mirg* deletion affects energy homeostasis, causing neonatal lethality (Labialle et al., 2014), and behavioral disturbances (Lackinger et al., 2019; Marty et al., 2016). However, the overall role of *Mirg* and its individual miRNAs in the early nervous system and cortical layering has not been deeply defined, with few exceptions (Marty and Cavaillé, 2019; Winter, 2015). For some of these miRNAs, a neurogenic function has been shown or proposed, but several seem involved in brain disorders (Gallego et al., 2016; Shi et al., 2015; Tsan et al., 2016; Winter, 2015). An overall GO analysis of the targets of these miRNAs pointed to embryonic and neural development and especially at axon guidance as key enriched terms; the possible involvement of *Mirg* in the regulation of key factors for CC formation was suggested by *in silico* target analysis (Glazov et al., 2008). It may be notable that mRNAs for axon guidance molecules, identified as targets of other miRNAs of *Mirg* (Glazov et al., 2008), are also *in silico* targets of miR-541; conversely, some of miR-541's most relevant targets (Figure S7) are also targets of other miRNAs of *Mirg*. Thus, the coordinate action of *Mirg* miRNAs in endowing the eutherian brain with some of its characters is an attractive hypothesis.

Satb2 is present in all vertebrates (Sheehan-Rooney et al., 2010) and is expressed with other CITF genes in the dorsal telencephalon (pallium) of birds, reptiles, and mammals, although with different patterns of mutual co-expression (Nomura et al., 2018; Tosches and Laurent, 2019). In the early mammalian neocortex, SATB2 efficiently binds the *Bcl11b* promoter and prevents its expression, although at later stages LMO4 relieves this inhibition (Alcamo et al., 2008; Britanova et al., 2008; Harb et al., 2016). In contrast, in reptilian and avian pallial cells, SATB2 cannot silence *Bcl11b*, due to inefficient binding to *Bcl11b cis*-regulatory sequences, and SATB2 and BCL11B are co-expressed (Nomura et al., 2018). By partitioning these two proteins in separate layers, this change may have increased cortical heterogeneity in the mammalian brain (Nomura et al., 2018). It looks possible that, on top of mutual transcriptional regulation, heterochronic gene modulation also takes place in brain development. Notably, in higher primates, SATB2 appearance is delayed over an extended period, possibly crucial for cortical expansion, during which deep layer neurogenesis is balanced with the expansion of PCs (Otani et al., 2016). Altogether, these observations indicate that tight temporal control and initial repression of SATB2 expression (Paolino et al., 2020) (present work) may hold a crucial role in pallial evolution.

EXPERIMENTAL PROCEDURES

mESC corticalization *in vitro*, cell transfection, and analysis were performed as previously described (Terrigno et al., 2018a, 2018b). hiPSCs (ATCC-DYS0100 line, American Type Culture Collection) were neuralized according to Chambers et al. (2009).

COTAN was performed on previously published datasets (Yuzwa et al., 2017) according to Galfrè et al., (2020). EISA was performed as described (Gaidatzis et al., 2015; La Manno et al., 2018) on available datasets (Chui et al., 2020). RNA immunoprecipitation, small RNA-seq, and miR-CATCH were carried out as described (Marranci et al., 2019; Pandolfini et al., 2016), with minor modifications.

miRNA-mRNA *in silico* affinity was predicted as described (Enright et al., 2003), using score >120, energy < -18 kcal/mol as thresholds. 3′UTR sequences were obtained from Ensembl resources (Hunt et al., 2018), using Cran Biomart package. miRNA sequences were obtained from miRBase database (v.22) (Kozomara et al., 2019). Detailed material and methods are described in supplemental information.

Data and code availability

The accession numbers for the sequencing data reported in this paper are GEO: GSE172502 (small RNA-seq) and GEO: GSE172503 (RNA-seq).

SUPPLEMENTAL INFORMATION

Supplemental information can be found online at https://doi.org/10.1016/j.stemcr.2021.04.020.

AUTHOR CONTRIBUTIONS

M.M., R.V., L. Poliseno, and F.C. designed the experiments. M.M. performed cell culture, molecular biology, imaging, and gene expression data computation. P.M. and I.A. planned and carried out IUE. S.G. and F.M. performed COTAN and EISA. M.P. and M.H.C. advised on computational methods of COTAN, EISA, and small RNA-seq analysis. K.D. performed hiPSC cultures. M.T. and L. Pandolfini. performed small RNA-seq. L. Pandolfini. carried out AGO RIP and RNA-seq. M.R. and A. Marranci carried out miR-CATCH. A. Mercatanti performed the computational analysis of captured miRNAs. R.V. and F.C. wrote the paper.

DECLARATION OF INTERESTS

The authors declare no competing interests.

ACKNOWLEDGMENTS

We thank G. Lupo, M.A. Tosches, R. Harland, and M. Gotz for critical review of the manuscript; M.A. Calvello and E. Sozzi for technical support; L. Conti for induced pluripotent stem cell neural differentiation methods advice; and A. Smith for embryonic stem cell lines. This work was supported by Ministry of University and Research grant PRIN-2102 (F.C.), by the EU Commission FP7 PAINCAGE Project grant number 603191, and the H2020-ICT-2016 MADIA Project grant number 732678.

Received: October 26, 2020
Revised: April 26, 2021
Accepted: April 26, 2021
Published: May 20, 2021

REFERENCES

Alcamo, E.A., Chirivella, L., Dautzenberg, M., Dobreva, G., Fariñas, I., Grosschedl, R., and McConnell, S.K. (2008). Satb2 regulates callosal projection neuron identity in the developing cerebral cortex. Neuron *57*, 364–377.

Bartel, D.P. (2009). MicroRNAs: target recognition and regulatory functions. Cell *136*, 215–233.

Bertacchi, M., Pandolfini, L., D'Onofrio, M., Brandi, R., and Cremisi, F. (2015). The double inhibition of endogenously produced BMP and Wnt factors synergistically triggers dorsal telencephalic differentiation of mouse ES cells: in vitro mouse ES cell corticalization. Dev. Neurobiol. *75*, 66–79.

Bian, S., Hong, J., Li, Q., Schebelle, L., Pollock, A., Knauss, J.L., Garg, V., and Sun, T. (2013). MicroRNA cluster miR-17-92 regulates neural stem cell expansion and transition to intermediate progenitors in the developing mouse neocortex. Cell Rep. *3*, 1398–1406.

Britanova, O., de Juan Romero, C., Cheung, A., Kwan, K.Y., Schwark, M., Gyorgy, A., Vogel, T., Akopov, S., Mitkovski, M., Agoston, D., et al. (2008). Satb2 is a postmitotic determinant for upper-layer neuron specification in the neocortex. Neuron *57*, 378–392.

Chambers, S.M., Fasano, C.A., Papapetrou, E.P., Tomishima, M., Sadelain, M., and Studer, L. (2009). Highly efficient neural conversion of human ES and iPS cells by dual inhibition of SMAD signaling. Nat. Biotechnol. *27*, 275–280.

Chen, B., Wang, S.S., Hattox, A.M., Rayburn, H., Nelson, S.B., and McConnell, S.K. (2008). The Fezf2-Ctip2 genetic pathway regulates the fate choice of subcortical projection neurons in the developing cerebral cortex. Proc. Natl. Acad. Sci. U S A *105*, 11382–11387.

Chiu, H., Alqadah, A., and Chang, C. (2014). The role of microRNAs in regulating neuronal connectivity. Front. Cell. Neurosci. *7*, 283.

Chui, A., Zhang, Q., Dai, Q., and Shi, S.-H. (2020). Oxidative stress regulates progenitor behavior and cortical neurogenesis. Development *147*, dev184150.

Cubelos, B., Sebastián-Serrano, A., Beccari, L., Calcagnotto, M.E., Cisneros, E., Kim, S., Dopazo, A., Alvarez-Dolado, M., Redondo, J.M., Bovolenta, P., et al. (2010). Cux1 and Cux2 regulate dendritic branching, spine morphology, and synapses of the upper layer neurons of the cortex. Neuron *66*, 523–535.

Dehay, C., and Kennedy, H. (2007). Cell-cycle control and cortical development. Nat. Rev. Neurosci. *8*, 438–450.

Dehay, C., Kennedy, H., and Kosik, K.S. (2015). The outer subventricular zone and primate-specific cortical complexification. Neuron *85*, 683–694.

Ding, J., Li, X., and Hu, H. (2016). TarPmiR: a new approach for microRNA target site prediction. Bioinformatics *32*, 2768–2775.

Edwards, C.A., Mungall, A.J., Matthews, L., Ryder, E., Gray, D.J., Pask, A.J., Shaw, G., Graves, J.A.M., Rogers, J., the SAVOIR consortium, et al. (2008). The evolution of the DLK1-DIO3 imprinted domain in mammals. PLoS Biol. *6*, e135.

Enright, A.J., John, B., Gaul, U., Tuschl, T., Sander, C., and Marks, D.S. (2003). MicroRNA targets in *Drosophila*. Genome Biol. *14*, R1.

Gaidatzis, D., Burger, L., Florescu, M., and Stadler, M.B. (2015). Analysis of intronic and exonic reads in RNA-seq data characterizes transcriptional and post-transcriptional regulation. Nat. Biotechnol. *33*, 722–729.

Galfrè, S.G., Morandin, F., Pietrosanto, M., Cremisi, F., and Helmer-Citterich, M. (2020). COTAN: co-expression table analysis for scRNA-seq data. BioRxiv. https://doi.org/10.1101/2020.05.11.088062.

Gallego, A., Melé, M., Balcells, I., García-Ramallo, E., Torruella-Loran, I., Fernández-Bellon, H., Abelló, T., Kondova, I., Bontrop, R., Hvilsom, C., et al. (2016). Functional implications of human-specific changes in great ape microRNAs. PLoS One *11*, e0154194.

Gaspard, N., Bouschet, T., Hourez, R., Dimidschstein, J., Naeije, G., van den Ameele, J., Espuny-Camacho, I., Herpoel, A., Passante, L., Schiffmann, S.N., et al. (2008). An intrinsic mechanism of corticogenesis from embryonic stem cells. Nature *455*, 351–357.

Glazov, E.A., McWilliam, S., Barris, W.C., and Dalrymple, B.P. (2008). Origin, evolution, and biological role of miRNA cluster in DLK-DIO3 genomic region in placental mammals. Mol. Biol. Evol. *25*, 939–948.

Greig, L.C., Woodworth, M.B., Galazo, M.J., Padmanabhan, H., and Macklis, J.D. (2013). Molecular logic of neocortical projection neuron specification, development and diversity. Nat. Rev. Neurosci. *14*, 755–769.

Gulman, N.K., Armon, L., Shalit, T., and Urbach, A. (2019). Heterochronic regulation of lung development via the Lin28-Let-7 pathway. FASEB J. *33*, 12008–12018.

Han, Z., He, H., Zhang, F., Huang, Z., Liu, Z., Jiang, H., and Wu, Q. (2012). Spatiotemporal expression pattern of Mirg, an imprinted non-coding gene, during mouse embryogenesis. J. Mol. Hist. *43*, 1–8.

Harb, K., Magrinelli, E., Nicolas, C.S., Lukianets, N., Frangeul, L., Pietri, M., Sun, T., Sandoz, G., Grammont, F., Jabaudon, D., et al. (2016). Area-specific development of distinct projection neuron subclasses is regulated by postnatal epigenetic modifications. ELife *5*, e09531.

Hevner, R.F., Shi, L., Justice, N., Hsueh, Y.-P., Sheng, M., Smiga, S., Bulfone, A., Goffinet, A.M., Campagnoni, A.T., and Rubenstein, J.L.R. (2001). Tbr1 regulates differentiation of the preplate and layer 6. Neuron *29*, 353–366.

Hevner, R.F., Daza, R.A.M., Rubenstein, J.L.R., Stunnenberg, H., Olavarria, J.F., and Englund, C. (2003). Beyond laminar fate: toward a molecular classification of cortical projection/pyramidal neurons. Dev. Neurosci. *25*, 139–151.

Hunt, S.E., McLaren, W., Gil, L., Thormann, A., Schuilenburg, H., Sheppard, D., Parton, A., Armean, I.M., Trevanion, S.J., Flicek, P., et al. (2018). Ensembl variation resources. Database *2018*, 1–12. https://doi.org/10.1093/database/bay119.

Jaitner, C., Reddy, C., Abentung, A., Whittle, N., Rieder, D., Delekate, A., Korte, M., Jain, G., Fischer, A., Sananbenesi, F., et al. (2016). Satb2 determines miRNA expression and long-term memory in the adult central nervous system. ELife *5*, e17361.

Kosik, K.S., and Nowakowski, T. (2018). Evolution of new miRNAs and cerebro-cortical development. Annu. Rev. Neurosci. *41*, 119–137.

Kozomara, A., Birgaoanu, M., and Griffiths-Jones, S. (2019). miRBase: from microRNA sequences to function. Nucleic Acids Res. *47*, D155–D162.

La Manno, G., Soldatov, R., Zeisel, A., Braun, E., Hochgerner, H., Petukhov, V., Lidschreiber, K., Kastriti, M.E., Lönnerberg, P., Furlan, A., et al. (2018). RNA velocity of single cells. Nature *560*, 494–498.

Labialle, S., Marty, V., Bortolin-Cavaillé, M., Hoareau-Osman, M., Pradère, J., Valet, P., Martin, P.G., and Cavaillé, J. (2014). The miR-379/miR-410 cluster at the imprinted Dlk1-Dio3 domain controls neonatal metabolic adaptation. EMBO J. *33*, 2216–2230.

Lackinger, M., Sungur, A.Ö., Daswani, R., Soutschek, M., Bicker, S., Stemmler, L., Wüst, T., Fiore, R., Dieterich, C., Schwarting, R.K., et al. (2019). A placental mammal-specific micro RNA cluster acts as a natural brake for sociability in mice. EMBO Rep. *20*, e46429.

Leone, D.P., Heavner, W.E., Ferenczi, E.A., Dobreva, G., Huguenard, J.R., Grosschedl, R., and McConnell, S.K. (2015). Satb2 regulates the differentiation of both callosal and subcerebral projection neurons in the developing cerebral cortex. Cereb. Cortex *25*, 3406–3419.

Lodato, S., and Arlotta, P. (2015). Generating neuronal diversity in the mammalian cerebral cortex. Annu. Rev. Cell Dev. Biol. *31*, 699–720.

Marranci, A., D'Aurizio, R., Vencken, S., Mero, S., Guzzolino, E., Rizzo, M., Pitto, L., Pellegrini, M., Chiorino, G., Greene, C.M., et al. (2019). Systematic evaluation of the microRNAome through miR-CATCHv2.0 identifies positive and negative regulators of BRAF -X1 mRNA. RNA Biol. *16*, 865–878.

Marty, V., and Cavaillé, J. (2019). Imprinted small noncoding RNA genes in brain function and behaviour. Curr. Opin. Behav. Sci. *25*, 8–14.

Marty, V., Labialle, S., Bortolin-Cavaillé, M.-L., Ferreira De Medeiros, G., Moisan, M.-P., Florian, C., and Cavaillé, J. (2016). Deletion of the miR-379/miR-410 gene cluster at the imprinted Dlk1-Dio3 locus enhances anxiety-related behaviour. Hum. Mol. Genet. *25*, 728–739.

McConnell, S.K., and Kaznowski, C.E. (1991). Cell cycle dependence of laminar determination in developing neocortex*254*, 4.

McKenna, W.L., Ortiz-Londono, C.F., Mathew, T.K., Hoang, K., Katzman, S., and Chen, B. (2015). Mutual regulation between Satb2 and Fezf2 promotes subcerebral projection neuron identity in the developing cerebral cortex. Proc. Natl. Acad. Sci. U S A *112*, 11702–11707.

Nomura, T., Yamashita, W., Gotoh, H., and Ono, K. (2018). Species-specific mechanisms of neuron subtype specification reveal evolutionary plasticity of amniote brain development. Cell Rep. *22*, 3142–3151.

Nowakowski, T.J., Fotaki, V., Pollock, A., Sun, T., Pratt, T., and Price, D.J. (2013). MicroRNA-92b regulates the development of intermediate cortical progenitors in embryonic mouse brain. Proc. Natl. Acad. Sci. U S A *110*, 7056–7061.

Nowakowski, T.J., Rani, N., Golkaram, M., Zhou, H.R., Alvarado, B., Huch, K., West, J.A., Leyrat, A., Pollen, A.A., Kriegstein, A.R., et al. (2018). Regulation of cell-type-specific transcriptomes by microRNA networks during human brain development. Nat. Neurosci. *21*, 1784–1792.

Otani, T., Marchetto, M.C., Gage, F.H., Simons, B.D., and Livesey, F.J. (2016). 2D and 3D stem cell models of primate cortical development identify species-specific differences in progenitor behavior contributing to brain size. Cell Stem Cell *18*, 467–480.

Pandolfini, L., Luzi, E., Bressan, D., Ucciferri, N., Bertacchi, M., Brandi, R., Rocchiccioli, S., D'Onofrio, M., and Cremisi, F. (2016). RISC-mediated control of selected chromatin regulators stabilizes ground state pluripotency of mouse embryonic stem cells. Genome Biol. *17*, 94.

Paolino, A., Fenlon, L.R., Kozulin, P., Haines, E., Lim, J.W.C., Richards, L.J., and Suárez, R. (2020). Differential timing of a conserved transcriptional network underlies divergent cortical projection routes across mammalian brain evolution. Proc. Natl. Acad. Sci. U S A *117*, 10554–10564, 201922422.

Robinton, D.A., Chal, J., Lummertz da Rocha, E., Han, A., Yermalovich, A.V., Oginuma, M., Schlaeger, T.M., Sousa, P., Rodriguez, A.,

Urbach, A., et al. (2019). The lin28/let-7 pathway regulates the mammalian caudal body axis elongation program. Dev. Cell *48*, 396–405.e3.

da Rocha, S.T., Edwards, C.A., Ito, M., Ogata, T., and Ferguson-Smith, A.C. (2008). Genomic imprinting at the mammalian Dlk1-Dio3 domain. Trends Genet. *24*, 306–316.

Sessa, A., Mao, C., Hadjantonakis, A.-K., Klein, W.H., and Broccoli, V. (2008). Tbr2 directs conversion of radial glia into basal precursors and guides neuronal amplification by indirect neurogenesis in the developing neocortex. Neuron *60*, 56–69.

Sheehan-Rooney, K., Pálinkášová, B., Eberhart, J.K., and Dixon, M.J. (2010). A cross-species analysis of Satb2 expression suggests deep conservation across vertebrate lineages. Dev. Dyn. *239*, 3481–3491.

Shi, X., Yan, C., Liu, B., Yang, C., Nie, X., Wang, X., Zheng, J., Wang, Y., and Zhu, Y. (2015). miR-381 regulates neural stem cell proliferation and differentiation via regulating Hes1 expression. PLoS One *10*, e0138973.

Shu, P., Wu, C., Ruan, X., Liu, W., Hou, L., Fu, H., Wang, M., Liu, C., Zeng, Y., Chen, P., et al. (2019). Opposing gradients of microRNA expression temporally pattern layer formation in the developing neocortex. Dev. Cell *49*, 764–785.e4.

Srinivasan, K., Leone, D.P., Bateson, R.K., Dobreva, G., Kohwi, Y., Kohwi-Shigematsu, T., Grosschedl, R., and McConnell, S.K. (2012). A network of genetic repression and derepression specifies projection fates in the developing neocortex. Proc. Natl. Acad. Sci. U S A *109*, 19071–19078.

Sturm, M., Hackenberg, M., Langenberger, D., and Frishman, D. (2010). TargetSpy: a supervised machine learning approach for microRNA target prediction. BMC Bioinformatics *11*, 292.

Sun, T., and Hevner, R.F. (2014). Growth and folding of the mammalian cerebral cortex: from molecules to malformations. Nat. Rev. Neurosci. *15*, 217–232.

Tarazona, S., García, F., Ferrer, A., Dopazo, J., and Conesa, A. (2012). NOIseq: a RNA-seq differential expression method robust for sequencing depth biases. EMBnet J. *17*, 18.

Tashiro, K., Teissier, A., Kobayashi, N., Nakanishi, A., Sasaki, T., Yan, K., Tarabykin, V., Vigier, L., Sumiyama, K., Hirakawa, M., et al. (2011). A mammalian conserved element derived from SINE displays enhancer properties recapitulating Satb2 expression in early-born callosal projection neurons. PLoS One *6*, e28497.

Telley, L., Agirman, G., Prados, J., Amberg, N., Fièvre, S., Oberst, P., Bartolini, G., Vitali, I., Cadilhac, C., Hippenmeyer, S., et al. (2019). Temporal patterning of apical progenitors and their daughter neurons in the developing neocortex. Science *364*, eaav2522.

Terrigno, M., Busti, I., Alia, C., Pietrasanta, M., Arisi, I., D'Onofrio, M., Caleo, M., and Cremisi, F. (2018a). Neurons generated by mouse ESCs with hippocampal or cortical identity display distinct projection patterns when co-transplanted in the adult brain. Stem Cell Reports *10*, 1016–1029.

Terrigno, M., Bertacchi, M., Pandolfini, L., Baumgart, M., Calvello, M., Cellerino, A., Studer, M., and Cremisi, F. (2018b). The microRNA miR-21 is a mediator of FGF8 action on cortical COUP-TFI translation. Stem Cell Reports *11*, 756–769.

Tosches, M.A., and Laurent, G. (2019). Evolution of neuronal identity in the cerebral cortex. Curr. Opin. Neurobiol. *56*, 199–208.

Tsan, Y., Morell, M.H., and O'Shea, K.S. (2016). miR-410 controls adult SVZ neurogenesis by targeting neurogenic genes. Stem Cell Res. *17*, 238–247.

Vencken, S., Hassan, T., McElvaney, N.G., Smith, S.G.J., and Greene, C.M. (2015). miR-CATCH: MicroRNA capture affinity technology. In RNA Interference, M. Sioud, ed. (Springer New York), pp. 365–373.

Winter, J. (2015). MicroRNAs of the miR379–410 cluster: new players in embryonic neurogenesis and regulators of neuronal function. Neurogenesis *2*, e1004970.

Yuzwa, S.A., Borrett, M.J., Innes, B.T., Voronova, A., Ketela, T., Kaplan, D.R., Bader, G.D., and Miller, F.D. (2017). Developmental emergence of adult neural stem cells as revealed by single-cell transcriptional profiling. Cell Rep. *21*, 3970–3986.

Zahr, S.K., Yang, G., Kazan, H., Borrett, M.J., Yuzwa, S.A., Voronova, A., Kaplan, D.R., and Miller, F.D. (2018). A translational repression complex in developing mammalian neural stem cells that regulates neuronal specification. Neuron *97*, 520–537.e6.

Zahr, S.K., Kaplan, D.R., and Miller, F.D. (2019). Translating neural stem cells to neurons in the mammalian brain. Cell Death Differ. *26*, 2495–2512.

Zhang, J., Zhang, J., Liu, L., Zhou, Y., Li, Y., Shao, Z., Wu, Y., Li, M., Fan, Y., and Shi, H. (2011). Effects of miR-541 on neurite outgrowth during neuronal differentiation. Cell Biochem. Funct. *29*, 279–286.

Stem Cell Reports, Volume *16*

Supplemental Information

A eutherian-specific microRNA controls the translation of Satb2 in a model of cortical differentiation

Manuella Martins, Silvia Galfrè, Marco Terrigno, Luca Pandolfini, Irene Appolloni, Keagan Dunville, Andrea Marranci, Milena Rizzo, Alberto Mercatanti, Laura Poliseno, Francesco Morandin, Marco Pietrosanto, Manuela Helmer-Citterich, Paolo Malatesta, Robert Vignali, and Federico Cremisi

Supplemental Figures

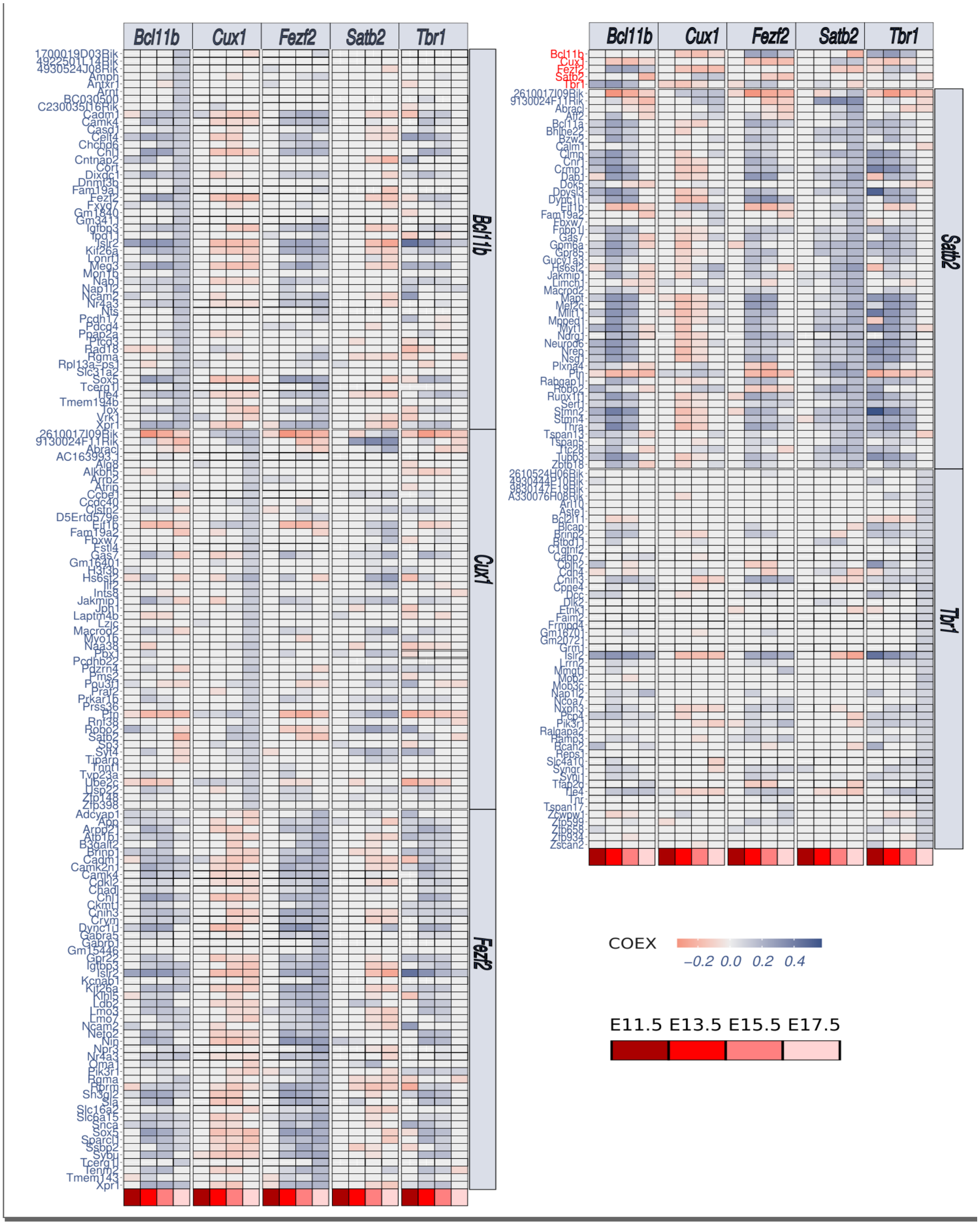

Figure S1. COEX values of genes related to CITFs, Related to Figure 1. Heatmap shows details of Figure 1D.

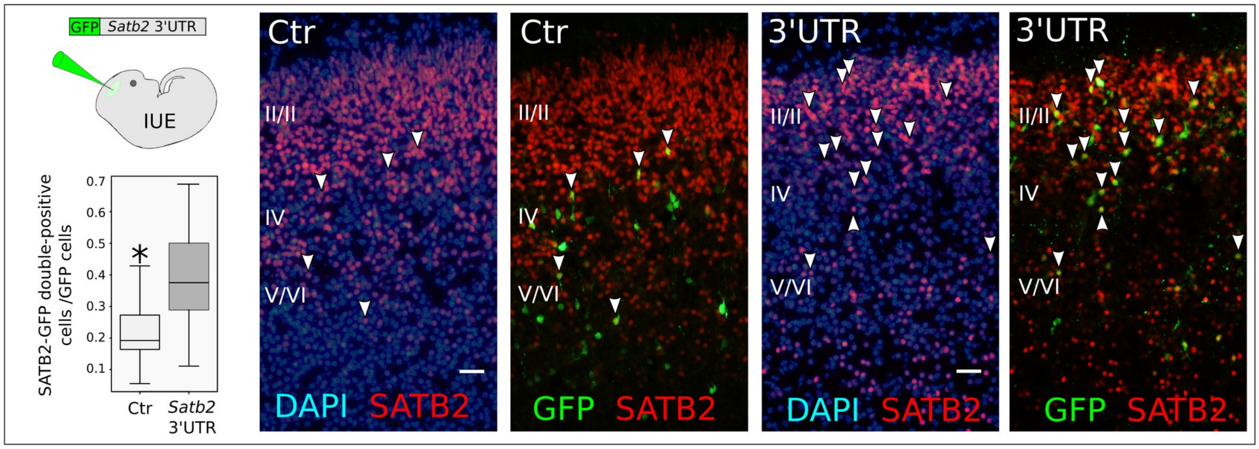

Figure S2. Expression of a GFP reporter bearing *Satb2* 3’ UTR, Related to Figure 3. IUE at E13.5 of a reporter as in the scheme. Box plot indicates the ratio of GFP-SATB2 double-positive cells out of all GFP-positive cells. Pictures show examples of IUE cells at E19. Arrowheads point to GFP-SATB2 double positive cells. Roman numbers indicate layers. Scale bar, 50 µm. Data from n= 3 animals for control IUE and n=3 animals for 3’ UTR IUE.

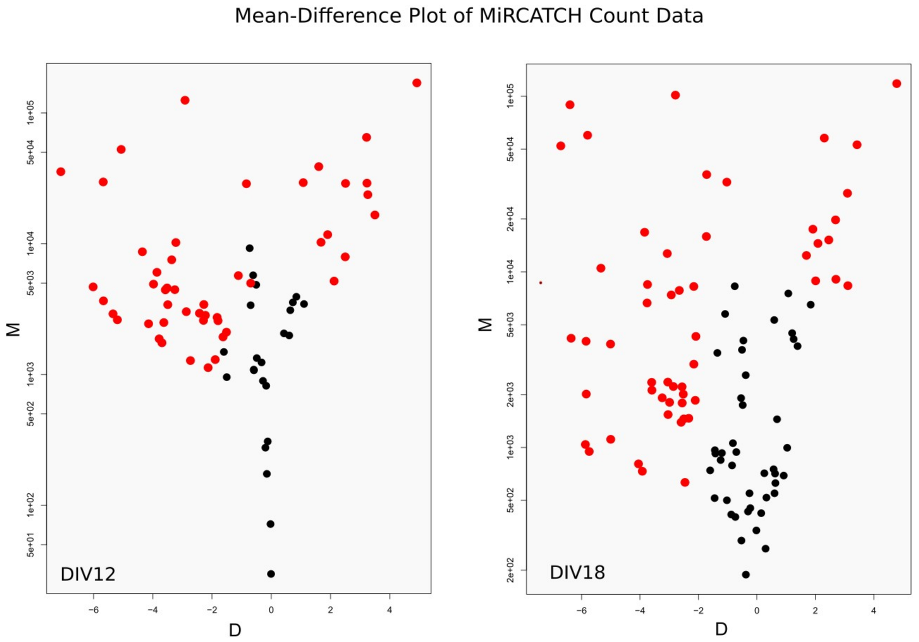

Figure S3. miR-CATCH M-D plot, Related to Figure 5. The Mean (M) - Difference (D) plot reported in figure shows the results of miRNA capturing by miR-CATCH as obtained by analysis with the NOIseqbio R package. Plots show the distribution of miRNAs enriched (positive $\log_2$ fold change, D) or depleted (negative $\log_2$ fold change) after miR-CATCH capturing, with respect to average expression (M, mean CPM), at the indicated time of differentiation (DIV12, DIV18). In red, miRNAs with significant fold change (probability > 0.9).

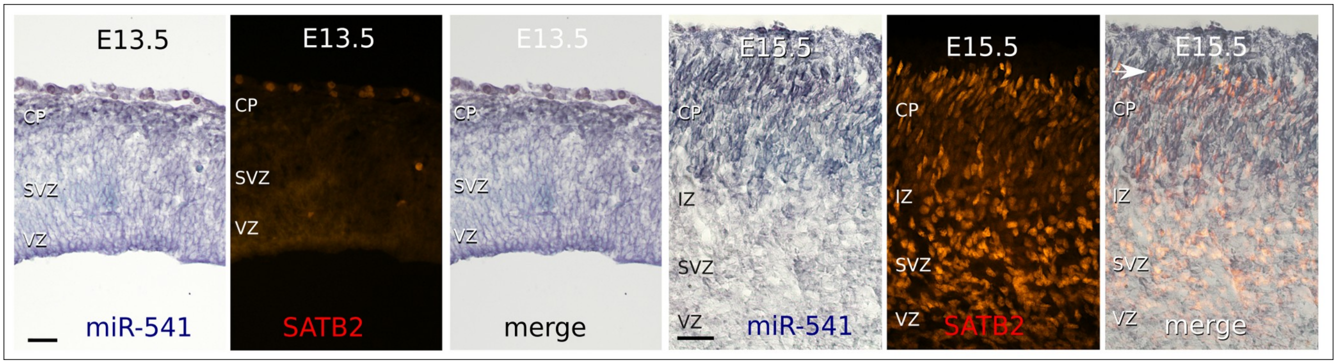

Figure S4. mir-541 expression, Related to Figure 5. *In situ* hybridization shows miR-541 distribution (BM-purple staining) compared to SATB2 immunodetection (red fluorescence). CP, cortical plate; IZ, intermediate zone; SVZ, subventricular zone; VZ, ventricular zone. The arrow indicates newly migrated SATB2-positive cells. Scale bar, 50 µm.

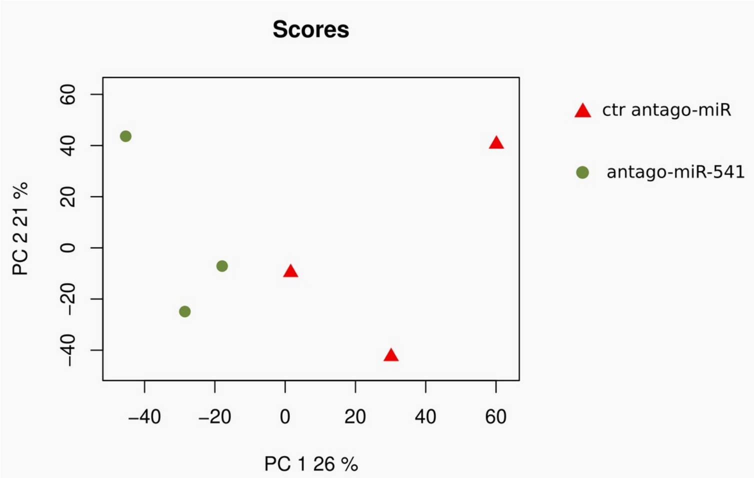

Figure S5. Transcriptome PCA, Related to Figure 6. PCA of corticalized ES cells transfected with antago-miR-541 and control antago-miR at DIV12 and analyzed by RNA-seq at DIV17. N= 3 independent experiments. The first component discriminates between control and experimental cells.

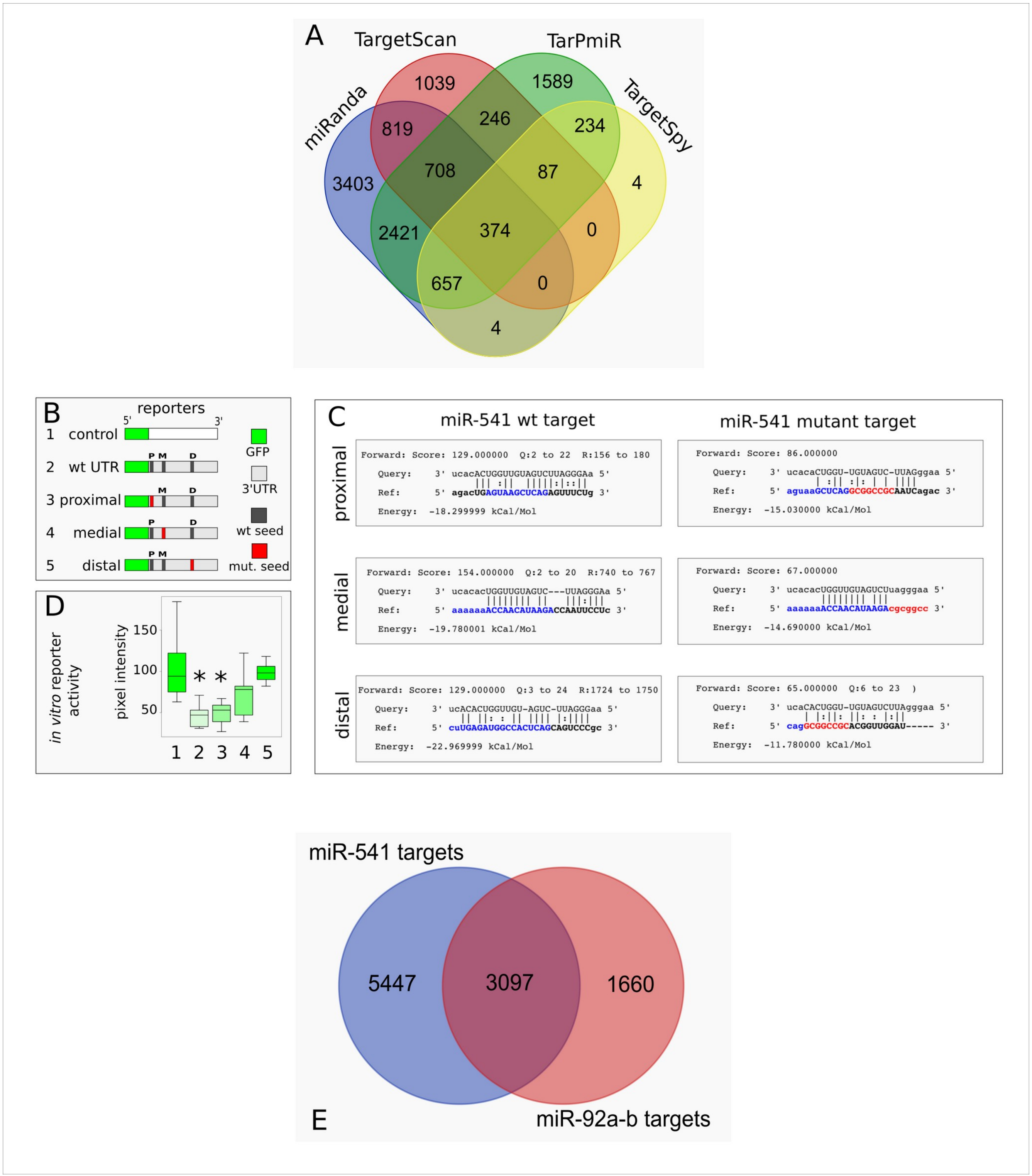

Figure S6. microRNA target analysis, Related to Figure 7. A**,** Venn diagram shows the intersections between genes targeted *in silico* by miR-541 as predicted by the four indicated tools (see Data S5). miRanda was compared to: TargetScan, which especially rewards the seed region (Bartel, 2009); TargetSpy, which predicts targets regardless of the presence of a seed match (Sturm et al., 2010); the ultimate tool TarPmiR, which can utilize miRNA-mRNA binding experiment data such as CLASH and applies a random-forest-based approach to integrate both conventional and new features (Ding et. al, 2016). B-D, GFP reporter analysis of *Satb2* 3'UTR**.** B, bars outline the reporter sequences used in the analysis of the *Satb2* 3' UTR. Wt and mutated sites of miRNA-mRNA interaction predicted by miRanda are depicted in black and red, respectively. The relative position of GFP

coding sequence is in green. C, panels show the interactions predicted by miRanda between mmu-miR-541-5p and *Satb2* 3'UTR outlined in B. Score estimates the probability of interaction, energy reports the predicted chemical binding affinity. Query: miRNA sequence. Ref: UTR sequence, seed in red, upstream bases of interaction in blue. D, box plots report GFP pixel intensity in DIV 12 cells (3 independent experiments), 2 days after transfection of the constructs shown in B. Asterisks indicate significant decrease compared to control (Wilcoxon test). E. Venn diagram shows the intersection between miR541 and miR92a-b gene targets as predicted by miRanda.

Name	Biochemical role	Biological process	Phenotype	References
Gas7	Adaptor protein	Neurite outgrowth, growth arrest	AD, schizophrenia	doi:10.1074/jbc.M113.513119<br>doi:10.1073/pnas.95.19.11423<br>doi: 10.1074/jbc.M109.051094<br>doi: 10.1186/s13041-016-0238-y
Rbfox2	RNA binding protein	Neuronal mRNA splicing		doi:10.1128/MCB.25.22.10005–10016.2005<br>doi:10.1371/journal.pcbi.0030196<br>doi.org/10.1016/j.neuron.2018.01.020
PlexinA4	Semaphorin receptor	Cortical neuronal migration, axon guidance		doi: 10.1016/j.isci.2019.10.034<br>doi:10.1523/JNEUROSCI.4480-04.2005
Cntn2	GPI-linked member of the immunoglobulin superfamily, cell adhesion molecule	Neurite outgrowth, axonal adhesion	Smaller cortex, reduced corticothalamic axons, callosal and commissural defects	doi: 10.3389/fncel.2019.00454
Dcx	Brain-specific microtubule associated protein	Cytoskeletal regulator involved in migration, cortical layering, neurite extension	Lissencephaly, subcortical band heterotopia, epilepsy, cognitive disability	doi:10.1016/s0092-8674(00)80898-3 et al., 1998;<br>doi:10.1038/mp.2017.175
Zeb2	Transcription factor	Regulation of gene expression	Mowat-Wilson syndrome; defects in axonal growth; hippocampal and callosal defects	doi: 10.1093/hmg/ddv350<br>doi:10.1016/j.neuron.2015.01.018
Cdk5	Serine/threonine kinase	phosphorylation of various cytoskeletal proteins	Corticogenesis, neuronal migration, axonal projections, dendrite branching; callosal dysgenesis	doi: 10.1073/pnas.93.20.11173<br>doi: 10.1523/JNEUROSCI.18-16-06370.1998<br>doi:10.1242/dev.02854<br>doi:10.1523/JNEUROSCI.1014-07.2007
TCF4	Transcription factor	Regulation of gene expression	Pitt-Hopkins syndrome; schizophrenia; autism; callosal dysgenesis	doi: 10.1086/515582<br>doi:10.1007/s00439-011-1020-y<br>doi:10.1093/schbul/sbx164<br>doi: 10.1111/ejn.14674

Figure S7. miR541 target genes enriched in GO analysis, Related to Figure 7.

Supplemental Experimental Procedures

Mouse ES cell-derived neural cell cultures

Murine ES cell lines E14Tg2A (passages 25-38) and 46 C (transgenic *Sox1*-GFP ESC kindly provided by A. Smith, University of Cambridge, UK, passages 33–39) were used for *in vitro* corticalization. For expansion, ES cells were grown on gelatin-coated tissue culture dishes (pre-treated 10 minutes with 0.1% gelatin in PBS) at a density of $4x10^4$ cells/cm^2. ES cell medium, changed daily, contained GMEM (G5154, Sigma-Aldrich), 10% Fetal Calf Serum (12133C, Sigma-Aldrich), 2mM Glutamine (25030, ThermoFisher Scientific), 1mM sodium Pyruvate (25030, ThermoFisher Scientific), 1mM non-essential amino acids (NEAA, 11140, Sigma Aldrich), 0.05mM β-mercaptoethanol (M3148, Sigma Aldrich), 100 U/mL Penicillin/Streptomycin (15140, ThermoFisher Scientific) and 1000 U/mL recombinant mouse LIF (PMC9484, ThermoFisher Scientific). Chemically defined minimal medium (CDMM) for neural induction consisted of DMEM/F12 (21331-046, ThermoFisher Scientific), 2mM Glutamine, 1mM sodium Pyruvate, 0.1Mm NEAA, 0.05mM β-mercaptoethanol, 100 U/mL Penicillin/Streptomycin supplemented with N-2 Supplement 100X (175020, ThermoFisher Scientific), and B-27 Supplement minus Vitamin A 50X (125870, ThermoFisher Scientific). ES neuralization was performed in three steps. In Step-I, dissociated ES cells were washed with DMEM/F12, seeded on gelatin-coated culture dishes ($6.5x10^4$ cells per cm^2) and cultured in CDMM plus 2.5μM 53AH Wnt inhibitor (C5324-10, Cellagen Technology) and 0.25μM BMP inhibitor (SML0559, Sigma Aldrich), for 3 days. In Step-II, ES cells were dissociated and seeded ($6.5x10^4$ cells per cm^2) on Poly-ornithine (P3655 Sigma-Aldrich; 20 μg/mL in sterile water, 24 hours coating at 37°C) and natural mouse Laminin (23017015, ThermoFisher Scientific; 2.5 μg/mL in PBS, 24 hours coating at 37°C). Cells were cultured for 4 additional days in CDMM plus Wnt/BMP inhibitors, with daily medium change. Serum employed for Trypsin inactivation was removed by two washes in DMEM/F12. In Step-III, cells were dissociated and seeded ($1.25x10^5$ cells per cm^2) on Poly-Ornithine and Laminin coated wells. Subsequently, isocortical cultures were kept in CDMM plus Wnt/BMP inhibitors for four additional days. On the eleventh day of differentiation, DMEM/F12 was replaced with Neurobasal and NEAA were removed from the CDMM to avoid glutamate-induced excitotoxicity. Medium was changed daily until the day of cell fixation.

hiPSC-derived neural cell cultures

Neural cell cultures were differentiated from a commercial reprogrammed fibroblast line (ATCC-DYS0100 line, American Type Culture Collection). Cell neuralization was carried out essentially as described (Chambers et al., 2009), with minor modifications. Reprogrammed stem cells were seeded at $3x10^4$ cells/cm^2 cultured on 1:100 geltrex and maintained in Essential 8 medium for two days. After two days incubation, cultures were switched to neural differentiation media: DMEM/F12 1:1 (21331-046, ThermoFisher Scientific) containing 2mM Glutamine (25030, ThermoFisher Scientific), 1mM Sodium Pyruvate (11360070, ThermoFisher Scientific), 100 U/mL Penicillin-streptomycin (15140, ThermoFisher Scientific), 1mM Non-essential amino acids (11140, Sigma Aldrich), 0.05mM β-mercaptoethanol (M3148, Sigma Aldrich), 10μM 53AH (C5324-10, Cellagen Technology), 10μM LDN193189 hydrochloride (SML0559, Sigma Aldrich), 1μM RepSox (R0158, Sigma Aldrich), N-2 Supplement 100X (175020, ThermoFisher Scientific), and B-27 Supplement minus Vitamin A 50X (125870, ThermoFisher Scientific). After 10 days in neural differentiation medium, cells were displaced from substrate via incubation at 37ºC for 20 minutes in Accutase solution (A6964, Sigma Aldrich). Cells were harvested, diluted in 5 volumes of 1X PBS, centrifuged for 4 minutes, and replated at 10^5 cells/cm^2 on poly-ornithine (P3655, Sigma Aldrich)/recombinant human Laminin (AMS.892 021, Amsbio) in half volume of neural differentiation media + 5 μM Y-27632 (SM02, Cell Guidance Systems). Cells were maintained for 4 days in fresh neural differentiation media without ROCK inhibitor followed by an expansion of 7 days in neural differentiation media without TGFβ, WNT, and BMP inhibitors. After 11 days, cells were displaced again from substrate via incubation at 37ºC for 20 minutes in Accutase solution. Cells were harvested, diluted in 1X PBS at a volume 5 times that of Accutase, centrifuged for 4 minutes, and replated at $2.5x10^5$ cells/cm^2 on poly-ornithine (P3655, Sigma Aldrich)/purified mouse Laminin (CC095-M, Merck Millipore) in Eppendorf glass bottom dishes (H 0030 741 021, Eppendorf). Cells were maintained in neural differentiation media without inhibitors for 12 days and then switched to neuronal maintenance media based on Neurobasal (21103049, ThermoFisher Scientific) and containing 2mM Glutamine (25030, ThermoFisher Scientific), 1 mM Sodium Pyruvate (11360070, ThermoFisher Scientific), 100 U/mL Penicillin-streptomycin (15140, ThermoFisher Scientific), 0.05mM β-mercaptoethanol (M3148, Sigma Aldrich), Ascorbate, 0.5mM (A92902, Sigma Aldrich), Recombinant human BDNF, 20 ng/mL (NBP2-52006, Novus Biologicals), and B-27 Supplement minus Vitamin A 50X (125870, ThermoFisher Scientific) until fixation at DIV 42. Cells were fixed with 2% PFA warmed to 37ºC for 15 minutes at room temperature.

Cell transfection

Plasmid transfections in mouse cortical cells were performed in 24-multiwell plate using 1 μg plasmid DNA diluted in 2.5 μL/well of Lipofectamine 2000 (12566014, ThermoFisher Scientific) in a final volume of 0.5 mL/well

OPTI-MEM (31985062, ThermoFisher Scientific). Reporter activity plasmids were pEGFP-C1 (Clontech; control) and pEGFP-C1 fused to 3' UTR of *Satb2* between HindIII and XbaI sites.
LNA anti-miRNA (antagoMir) transfections in mouse cortical cells were performed using Lipofectamine 2000 according to the manufacturer's instructions. miRCURY LNA™ microRNA Inhibitors to miR-541-5p, miR92-3p and control antagomiR (MIMAT0003170, YI00199006 and MIMAT0000539, respectively) were resuspended in TE buffer (10mM Tris pH 7.5, 1mM EDTA) to a final concentration of 50µM. Cells were transfected in 24-well plate using 25pmol of LNA diluted in 2.5 µL/well of Lipofectamine 2000 in a final volume of 0.5 mL/well OPTI-MEM. After transfection, cells were incubated at 37°C and 5% CO2 for 4-6 hours and then the medium was replaced with complete Neurobasal medium (mouse cortical cells) or complete McCoy medium (HCT-116 cells).

Satb2 3'UTR cloning
The entire *Satb2*-3'UTR sequence (2802 bp) was obtained from Genome Reference Consortium Mouse Build 38 patch release 6 (GRCm38.p6) and amplified by PCR with Q5 High-Fidelity DNA Polymerase (M0491, NEB) with a forward and reverse primer carrying, correspondingly, a HindIII and XbaI restriction site at their 5'end (forward, CACAAAGCTTGTGAACTCCGCAGGCAGAGC; reverse, CACATCTAGAGCGTTTTATTTAACAACCAAAAAATTCTAACAGCC). The plasmid carrying the *Satb2*-3'UTR was constructed using mammalian expression vector pEGFP-C1 (Clontech) cut at HindIII position and XbaI positions inside the multiple cloning site and ligated with HindIII/XbaI restricted amplification product by T4 DNA ligase (M0202, NEB).
In order to identify potential target sites for our miRNA of interest (mmu-miR-541-5p) in the *Satb2* sequence, miRanda algorithm (v3.3a) (27) was used. MiRanda selected miR-541/*Satb2* binding sites with score >120 and energy < -18kd. Mutations in the three predicted sites were performed. The seed sequence of miR-541 at +156, +740 and +1724 were replaced with NotI restriction sequence (GCGGCCGC). To this aim, upstream and downstream halves of mutated 3'UTR were generated by PCR through external forward or reverse primer NotI together with a mutated internal reverse of forward primer, respectively. The mutated internal primers for miR-541 mutation at position +156 were miR-541_mutA_fw CACAGCGGCCGCAATCAGACGTCACCTTGGCAAAG and miR-541_mutA_rev CACAGCGGCCGCCTGAGCTTACTCAGTCTATAGGCTATCCTGTG. The mutated internal primers for miR-541 mutation at position +740 were mir-541_mutb_fw CACAGCGGCCGCCAGAGGACATAATGCACACCTTAAGAC and miR-541_mutB_rev CACAGCGGCCGCGGTCTTATGTTGGTTTTTTTGACATGCCC. The mutated internal primers for miR-541 mutation at position +1724 were miR-541_mutC_fw CACAGCGGCCGCGAGTTGTATCCTCATGCAACCTTGTC and miR-541_mutC_rev CACAGCGGCCGCCTGAGTGGCCATCTCAAGCC. After PCRs, both upstream and downstream mutated halves were digested with NotI enzyme, ligated and used as a template for PCR together with external forward and reverse primers (forward, CGCAGGCAGAGCAATAGATGG; reverse, GGCGGGAAATTGTGCTTTGTCAAGA). PCR products were cut with HindIII/XbaI restriction enzymes, purified and re-inserted in the pEGFP-C1 vector.

Immunocytodetection (ICD) and imaging
Cells prepared for immunocytodetection experiments were cultured on Poly-Ornithine/Laminin coated round glass coverslips. Cells were fixed using 2% paraformaldehyde for 12 minutes, washed twice with PBS, permeabilized using 0.1% Triton X100 in PBS and blocked using 0.5% BSA in PBS for 1 hr at RT. Embryonic cortical sections were thawed and let dry at room temperature 1 hr, then briefly washed three times (5 minutes each) in PBS before antibody staining.
Cells/slices were pre-treated 1 hr at RT with blocking solution: 1% BSA, 10% goat serum, 0.1% Triton X100 in PBS. Primary antibodies used for microscopy were SATB2 ab (1:1000; ab92446, Abcam), GFP ab (1:1000, ab13970, Abcam). Primary antibodies were incubated overnight at 4°C in PBS containing 1% BSA and 10% goat serum in PBS; cells/slices were then washed three times with PBS (10 minutes each). Alexa Fluor 488 and Alexa Fluor 546 anti-mouse, anti-rabbit or anti-chicken IgG conjugates (1:500; A32723, A-11034, A-11039, A-11003, A-11010, A11040, Molecular Probes) were incubated 1 hour at RT in PBS containing 1% BSA and 10% goat serum, followed by three PBS washes (10' each). Nuclear staining was obtained with DAPI (D1306, ThermoFisher Scientific). Cells/slices were coverslipped with Aqua Poly-mount (18606-100, Polysciences).
Mouse neural cells were imaged using a Nikon Eclipse E600 epifluorescence microscope with a 20X objective and a Photometrics Coolsnap CF camera. Five to ten optic fields from two or more biological replicates were acquired. In the experiments of EGFP pixel intensity quantification, all the pictures were acquired with the same parameters and the median of pixel intensity of the entire acquired field was analysed. For cell counting, double blind analysis was performed.
Human neural cells were imaged using a Leica SP2 confocal microscope with a 40X oil objective. Z-stacks were attained between 9-12 µm thick optical sections. Three biological replicates were attained per treatment group

and subdivided into 5 technological replicate Z-stacks resulting in 15 total acquisitions. Stacks were flattened in ImageJ (RRID:SCR_002285) using the Z-stack projection function, set as a representation of standard deviation, and backgrounds were subtracted as a function of disabled smoothing and rolling ball radius of 20 px2. The resultant Hoechst+ and SATB2+ images were then subjected to an automated cell counter in ImageJ macros which analyzed separate channels at a 16-bit threshold set between 30-65355, and individual cells were counted using the “Analyze Particle” function set at circularity 35-150 px2 and circularity 0.33-0.99 to only include positive nuclei and minimize false positives.

ScRNA-seq datasets

ScRNA-seq datasets available in literature (Yuzwa et al., 2017) were used to analyse cortical gene expression at E11.5, E13.5, E15.5 and E17.5. Raw counts were obtained from GEO:GSE107122 and used to plot counts/cell values by the vioplot R package.

COTAN

Co-expression Table Analysis (COTAN) aims to estimate the UMI detection efficiency (UDE) of each cell, finds an approximation of the probability of zero read counts for a gene in a cell, and test the null hypothesis of independent expression for gene pairs, by counting zero/non-zero UMI counts in single cells (co-submitted paper). Briefly, mitochondrial genes and genes expressed in less than 0.3% of cells were eliminated. UDE for each cell and average expression for each gene were estimated as described (Galfrè et al. 2020) (*linear* method was used). PCA and hierarchical clustering (two clusters) were then carried out on UMI counts normalized dividing them by UDE. After removal of cell outliers resulting from PCA and hierarchical clustering, UDE and average expression were estimated again. Cells with very low UDE values were also removed. Together the two cleaning steps removed in all the datasets less than 3% of the cells (E11.5 dropped from 1,418 cells to 1,379 cells, E13.5 dropped from 1,137 to 1,119, E15.5 dropped from 2,955 to 2,921, E17.5 dropped from 880 to 863 cells).

Expected values for contingency table analysis were obtained as described (Galfrè et al. 2020) using cells UDE and genes average expression estimated with linear method, and genes dispersion estimated by fitting the observed number of cells with zero UMI count. COTAN then provided both an approximate p-value for the test of independence and a signed co-expression index (COEX), which measures the direction and intensity of the deviation from the independence hypothesis. The heatmaps in Figure 1D are colored by COEX value (blue for co-expression and red for disjoint expression).

For each gene, GDI was computed by normalizing P, the 0.001 quantile of the p-values of COTAN test for co-expression with all other genes. Our chosen normalization is ln(-ln(pval)). Genes with GDI > 2.2, which corresponds to $\ln(-\ln(10^{-4}))$, were generally non constitutive genes (Galfrè et al., 2020). Plots were generated with ggplot2 in R environment. The following R packages were employed: matrixStats, ggfortify, dplyr, rray, propagate, data.table, ggsci, gmodels, parallel, tibble, ggrepel.

scRNA-seq bidimensional analysis

UMI counts were divided by COTAN UDE for normalization. PCA was performed with normalized counts in R environment. Eigenvalues were plotted for selection by “elbow” point analysis (the number of components used were: 10 for E11.5, 10 for E13.5, 15 for E15.5 and 10 for E17.5). Selected components were employed as input for t-SNE function in sklearn.manifold python package (Loo et al., 2019), using the following parameters: perplexity 30, number of iterations 7000 and learning rate 700. Plots were obtained by ggplot2 R package.

Cell cluster analysis by Seurat

The datasets from GSE107122 series were used and, in detail, the “Combined_Only_Cortical_Cells” matrixes were analysed for each time point of development. For the single cell RNAseq data clustering the standard workflow of the R package Seurat 4.0 was followed. No cleaning was needed and the detection of nearest neighbours was performed using respectively 10, 15, 15 and 20 principal components for the E11.5, E13.5, E15.5 and E17.5 datasets. For all samples, the original Louvain algorithm was used for the clustering with a resolution of 1. The same number of principal components were used to perform the Uniform Manifold Approximation and Projection (UMAP) dimensional reduction.

Exon-Intron split analysis (EISA)

EISA on mouse cortex transcriptomes of cortical progenitor cells at E11.5, E13.5, E15.5 and E17.5 (Chui et al., 2020) was performed as previously described in (Gaidatzis et al., 2015; La Manno et al., 2018), with modifications. Mapping of datasets to mouse genome annotation GRCm38.98 was carried out as described in https://www.kallistobus.tools/velocity_index_tutorial.html (La Manno et al., 2018). Briefly, by using USCS table

browser we obtained intron BED file, cDNA file and genome fasta files. A mouse GTF file was obtained from the Ensembl.t2g utility and used to map transcripts to gene map (https://github.com/sbooeshaghi/tools/releases/tag/t2g_v0.24.0). Intron BED file was converted to fasta format by bedtools (v2.25; https://github.com/arq5x/bedtools2/releases). Association of intron and exon identifiers was performed modifying the fasta file headers as described in (https://www.kallistobus.tools/velocity_index_tutorial.html). An index was eventually produced by Salmon (version 1.1.0) (Patro et al., 2017) using the modified fasta files. Read pseudo-counts obtained by Salmon were normalized as reads per million (RPM). Log_2 (CPM) expression levels (exonic and intronic) were calculated and the exons/introns ratio was defined as the difference between log_2 exonic pseudo-counts and log_2 intronic pseudo-counts for each experimental condition.

In Utero Electroporation (IUE)

All animal procedures were approved by the internal Ethical Committee for Animal Experimentation (OPBA) of the Ospedale Policlinico San Martino and by the Italian Ministry of Health according to the Italian law D. lgs 26/2014 and the European Directive 2010/63/EU of the European Parliament. In all the experiments, the C57BL/6J strain from Jackson Laboratory was used.
In utero intraventricular electroporation was performed on E13 mouse embryos following laparotomy of deeply anesthetized pregnant females. Embryos were injected within the telencephalic ventricles with approximately 2 µL (2 µg) of pEGFP-C1 (Clontech; control) or pEGFP-C1 bearing normal *Satb2* 3' UTR, which were immediately electroporated at 35V with 4 pulses lasting 50 ms and spaced by 950 ms with a NEPA21 (NepaGene, Chiba, Japan) electroporator. Brains were dissected 7 days after electroporation and fixed overnight at 4°C in 4% paraformaldehyde in PBS. Brains were then cryoprotected overnight in 20% sucrose, embedded in Tissue Teck O.C.T. compound (4583, Sakura) and sectioned with a Leica CM3050 S cryostat at 12 µm thickness.

RNA Immunoprecipitation

Cross-linking Immunoprecipitation (CLIP) was carried out to enrich AGO-interacting RNA. Cells were differentiated into cortical neurons until DIV12 or DIV18. Adherent cells were rinsed twice in PBS, cross-linked 150 mJ/cm^2 at 254 nm wave length, scraped, spun down 10 seconds at top speed and lysed on ice for 10 minutes in 1 mL of fresh lysis buffer (Tris-HCl 25mM pH 8.0, NaCl 150mM, $MgCl_2$ 2mM, 0.5% NP-40, DTT 5mM) with protease inhibitors (1 tablet/10 mL lysis buffer of EDTA-free Complete Protease Inhibitor Cocktail Tablets, 11697498001, Sigma Aldrich) and RNasin (250 U/mL final, N2115, Promega). Cell lysate was centrifuged at 10000 rpm at 4ºC for 10 minutes and the supernatant was kept at 4ºC for later procedure.
In the meantime, protein A Dynabeads (10001D, ThermoFisher Scientific) were rinsed 3 times with PBS/0.5% NP40 and incubated with 5 µg rabbit monoclonal Anti-argonaute-2 antibody EPR10411 (ab186733, Abcam), or anti-GFP antibody A-6455 (A-6455, ThermoFisher Scientific) in PBD/0.5% NP40 for 1 hour. After the initial binding, antibody-protein A beads were blocked with 0.5 mg/mL yeast RNA (10 µg/µL, 10109223001, Sigma Aldrich) and 1 mg/mL BSA (20 mg/mL, A3294-100G, Sigma Aldrich) for an additional 30 minutes; beads were then washed twice in PBS/0.5% NP40 to remove the unbound IgGs and then twice in lysis buffer. The beads were resuspended in 100 µL of lysis buffer.
The lysate was subjected to preclearance by incubation with pre-blocked Protein A beads at 4ºC for 60 minutes (100 µL of total lysate after pre-clearance, but before co-IP, was separated for total RNA – input – analysis). The remaining lysates proceeded to co-IP with anti-Ago-Protein A beads at 4ºC for 90 minutes. After incubation, beads were washed three times with lysis buffer, twice with lysis buffer high-salt content (Tris-HCl pH 8.0 25mM, NaCl 0.9 M, MgCl2 1mM, NP-40 1%, DTT 5Mm) and, again, once with lysis buffer. After washes, beads were incubated with 100 µL of SDS 0.1% and Proteinase K (0.5 mg/mL, P8107S, NEB) for 15 minutes at 55ºC.
RNAs that co-immunoprecipitated with anti-AGO or anti-GFP antibodies were extracted adding 700 µL Qiazol (79306, Qiagen) and 140 µL chlorophorm according to manual and then purified using Nucleospin RNA XS purification system (740902.50, Macherey-Nagel) following manufacturer's instructions.

Semiquantitative Real-Time PCR

RNA quantity and quality was measured using NanodropTM Lite UV Visible Spectrophotometer (ThermoFisher Scientific) followed by reverse transcriptase protocol. For each sample, 100 ng of total RNA were reverse transcribed. Reverse Transcriptase Core kit (RT-RTCK-03, Eurogentec) was employed for cDNA synthesis. Primers for amplification were 5'CATGAGCCCTGGTCTTCTCT3' (*Satb2* forward) and 5'AACTGCTCTGGGAATGGGTG3' (*Satb2* reverse). Amplified cDNA was quantified using Sensi Fast SYBR Green (BIO-98050, Bioline) on Rotor-Gene 6000 (Corbett). Amplification take-off values were evaluated using the built-in Rotor-Gene 6000 "relative quantification analysis" function and relative expression was calculated with the 2-ΔCt method.

Small RNA-Seq

Total RNA was extracted with miRNeasy Mini Kit (217004, QIAGEN). Small-RNA libraries were prepared using TruSeq Small RNA Sample Preparation Kit (RS-200-0012/24/36, Illumina) following the manufacturer's instructions starting from 1µg of total RNA per sample. Libraries were multiplexed, loaded into a V3 flow cell and sequenced in a single-reads mode (50 bp) on a MiSeq sequencer (Illumina), obtaining ~4 million reads per samples. Raw sequences were demultiplexed to FASTQ format using CASAVA v.1.8 (Illumina). Quality control checks were performed with the FastQC algorithm. Adapters were trimmed from the primary reads using Cutadapt v1.2.1 (Martin, 2011). Remaining reads, with a length of between 17bp and 35bp, were clustered by unique hits and mapped to pre-miRNA sequences (miRBase release 21) (Kozomara and Griffiths-Jones, 2014) with the miRExpress tool v 2.1.3 5 (Wang et al., 2009). Read counts were CPM normalized for comparative analyses. PCA was carried out by PCA.GENES R package.

miR-CATCH

miR-CATCH analysis (version 2.0) (Marranci et al., 2019; Vencken et al., 2015) was performed essentially as described, with minor modifications. Three biological replicas for each time of *in vitro* differentiation were included in the study. Mouse cells (>10^7/sample) were harvested at DIV12 and DIV18 of the cortical differentiation protocol by trypsinisation, washed with PBS and fixed with 1% formaldehyde for 10 minutes at room temperature. The reaction was quenched with 1.25M glycine for 5 minutes at room temperature and cells were centrifuged at 200g for 5 minutes at 4°C. The pellet was resuspended in ice cold PBS (50 mL) and centrifuged twice at the same conditions as previously. Cells were then resuspended in 1mL Lysis Buffer (50mM Tris-HCl pH 7.0, 5mM EDTA, 1% SDS) plus supplements: 1mM Phenylmethanesulfonyl fluoride (PMSF, P7626, Sigma Aldrich), 1X Protease Inhibitor Cocktail (P8340, Sigma Aldrich) and 80U/mL RNAsin (N2115, Promega); all the components were added freshly before use. Cells were sonicated in ice-cold Lysis Buffer with a Soniprep 150 ultrasonic disintegrator (MSS150.CX3.1, MSE) for 12 rounds at 70% amplitude for 30 seconds pulses with 45 second cool down pauses in between. Sonicated lysates were pooled in order to have a minimum of 1 mL to be hybridized with two probe pools, each containing 12 antisense biotin-labeled oligonucleotides, ODD or EVEN, as indicated in Table S1.

PROBE #	PROBE (5'-> 3')	PROBE POSITION *	PERCENT GC
**1**	aaagtccttggacccatcta	24	45.0%
**2**	tctgagcttactcagtctat	154	40.0%
**3**	cttccataagttggcaggaa	273	45.0%
**4**	attgtaaagttctctgtccc	408	40.0%
**5**	agtgactcactgtgaagtgg	492	50.0%
**6**	attacccattaaaagctgcc	627	40.0%
**7**	ctctggaggaattggtctta	753	45.0%
**8**	ctcgatacagtgctggcatg	835	55.0%
**9**	ggtccaacgtcaaaacgtca	928	50.0%
**10**	gaaggaaagggtaacaccct	1048	50.0%
**11**	tctaaccgggcagaaacttc	1231	50.0%
**12**	tctggctaaagtgaagggga	1336	50.0%
**13**	tcacttactttattgcctgg	1441	40.0%
**14**	tggcattagttctgctttac	1537	40.0%
**15**	ctggaaggtaatgctactgt	1635	45.0%
**16**	tgctgagtggccatctcaag	1724	55.0%
**17**	tgtattgcaacgtgtcttct	1976	40.0%
**18**	gctcatgtcaagggtaactg	2078	50.0%
**19**	ggagatcaggaagcagcaac	2196	55.0%
**20**	agagtgacttcagcaacagc	2245	50.0%
**21**	gatgccatcgatcgatgaac	2310	50.0%
**22**	aaatgcccacagattcactt	2436	40.0%
**23**	ctttgtcaagaggcactaca	2557	45.0%
**24**	acagcctaacaatgcacata	2739	40.0%

Table S1. miR-CATCH probes

Dynabeads MyOne Streptavidin C1 (65001, ThermoFisher Scientific) were washed (30 µL for each experiment) three times with 1 mL unsupplemented Lysis Buffer and resuspended in 30 µl complete Lysis Buffer. The beads were added to 1 mL lysate in a 1.5 mL tube and kept on rotation in a 37°C hybridization oven for 30 minutes. Then, the lysates were cleared from beads twice using a magnetic stand and transferred to a 5 mL round-bottom tube where 2 mL of supplemented Hybridization Buffer (750mM NaCl, 1% SDS, 50mM Tris-HCl pH 7.0, 1mM EDTA and 15% formamide plus supplements: 1mM PMSF, 1X protease inhibitor cocktail and 80U/mL RNAsin that were added fresh before use) were added. At this point, a total amount of 100pmol probes (capture ODD/EVEN or scrambled control probes, 1 µl from a 100 µl pool previously mixed) were added to each lysate and put again in rotation in a 37°C hybridization oven for 4 hours. While the probes were incubating with the lysate, 200 µL of beads were washed three times with unsupplemented Lysis Buffer and resuspended in 200 µL supplemented Lysis Buffer. 100 µL of beads were added to the lysate plus probes sample and rotated in the hybridization oven for an additional 30 minutes at 37°C. After this, beads were pelleted using the magnetic support and resuspended in 1 mL of Wash Buffer (2X SSC Buffer, 0.5% SDS and 1mM PMSF added fresh) pre-warmed at 37°C. Five washes of 5 minutes each using hybridization oven at 37°C were performed in rotation with the Wash Buffer. At the last wash, the beads were spun down and all the wash buffer was removed. Beads

were then resuspended in 185 µL Proteinase K buffer (100mM NaCl, 10mM Tris-HCl pH 7.0, 1mM EDTA, 0.5% SDS), added with 15 µL of 20 mg/mL Proteinase K and then incubated at 45°C for 1 hour under constant and vigorous agitation followed by 10 minutes incubation at 95°C. Finally, 1 mL Qiazol was added directly to the beads, vortexed and incubated for 10 minutes at room temperature. The RNA extraction was performed using Nucleospin RNA XS purification system (740902.50, Macherey-Nagel).

The RNA eluted from the ODD and EVEN samples were used to prepare cDNA libraries with the TruSeq Small RNA kit (RS-200-0012/24/36, Illumina), as per the manufacturer's suggestions. cDNA libraries were multiplexed, loaded into a V3 flow cell and sequenced in a single-reads mode (50 bp) on a MiSeq sequencer (Illumina), obtaining ~4 million reads per samples. Read counts reported in Data S3 were obtained as described in the miR-seq section method. To evaluate the enrichment of miRNA binding to *Satb2* 3'UTR, at each time of analysis (DIV12 or DIV18) *Satb2*-captured RNA from three independent experiments (DIV12: 3 EVEN and 2 ODD biological replicas; DIV18: 2 EVEN and 2 ODD biological replicas) and total RNA (DIV12 and DIV18, n=3) were compared. miRNA reads were normalized as CPM. Aiming to discover miRNAs with high biological relevance, those in the highest quartile of expression were considered for the analysis. The enrichment of miRNA binding to *Satb2* 3'UTR was evaluated as $\log_2$ captured/input fold change. The non-parametric NOISeqBIO statistical test of NOISeq R-package was applied with a probability >0.9 (Tarazona et al., 2015). In Data S3, log2FC indicates the fold change between captured and input miRNAs. Means refer to average CPM of captured (C) and input (control) miRNAs. Prob is the probability of differential expression calculated by NOIseqbio (Tarazona et al., 2015). Theta indicates differential expression statistics.

In situ hybridization

miRNA *in situ* hybridization (ISH) was performed using LNA-modified oligonucletide probes (Exiqon), according to the manufacturer protocol, with minor modifications. Cryosections were collected on slides (J1800AMNZT, Thermo Scientific) and postfixed 15 minutes with 4% paraformaldehyde (PFA) in PBS. Sections were treated with 10ng/µL proteinase K (15 minutes), washed with 2 mg/mL glycine (2x5 minutes), PBS (2x5 minutes), and postfixed 15 minutes with 4% PFA. Sections were then pre-hybridized (50 minutes) in hybridization solution containing: with 50% formamide, 5X sodium saline citrate buffer (SSC) (pH 6), 1% sodium dodecyl sulfate (SDS), 50 g/mL heparin (9041-08-1, ThermoFisher Scientific) and 500 g/mL yeast RNA (10109223001, Sigma Aldrich). Hybridization with the digoxigenin-labeled probes was performed overnight at a temperature approximately 21°C lower than the melting temperature of the probe. miRNA probes (miRNA Detection Probes, 339111, Exiqon) to mmu-miR-541-5p, and control probe with scrambled sequence, were employed. Washes were carried out in 50% formamide, 2XSSC at the hybridization temperature (1x30 minutes) and 1XSSC (2x15 minutes). Sections were blocked 30 minutes in MABT (1% BSA, A3294-100G, Sigma Aldrich; 150mM NaCl; 0.1% Tween 20, pH7.5) containing 10% sheep serum (S2263, Sigma Aldrich) and incubated with alkaline phosphatase (AP)-labeled anti-digoxigenin antibody (1:2000; 11093274910, Sigma Aldrich)) in MABT and 1% BSA, overnight at 4°C. Sections were washed 5x5 minutes in MABT and 3x5 minutes in NMNT (100mM NaCl, 100mM TrisHCl pH 9.5, 50mM MgCl, 0.1% Tween-20, 2mM Tetramisole (L9756-5G, Sigma Aldrich: 500 mg/L). Sections were eventually stained with BM-Purple AP-substrate (L9756-5G, Sigma Aldrich) at RT 0.5- 2 hours, then blocked by washes with PBS and counter-stained with anti-SATB2 antibody.

RNA-seq

RNA-seq libraries were prepared with the SMART-Seq® HT PLUS Kit (Takara) following manufacturer's instructions and sequenced on a NovaSeq instrument (Illumina), obtaining between 20-35M reads per sample.

Transcripts were quantified using Salmon (REF:10.1038/nmeth.4197) in mapping-based mode (with its default "--validateMappings" flag) taking as a reference a decoy-aware version of the Ensembl mouse transcriptome (mm10; refgenomes.databio.org).

RNA-seq analysis was performed using the R package NOISeq. Raw counts were normalized with the Trimmed Mean of M values (TMM) method. Low-count filtering was performed using the CPM method, with cpm=4 as threshold. PCA exploration was carried out to confirm that the experimental samples were clustered according to the experimental design (see Figure S5). Differential expression was calculated by the NOISeqBIO method and a significance threshold of q=0.95 was applied.

MiRNA-mRNA interaction prediction and GO enrichment

miRNA-mRNA *in silico* affinity was predicted as described (Enright et al., 2003), using score >120, energy < -18 kd as thresholds. 3'UTR sequences were obtained from Ensembl resources (Hunt et al., 2018), using Cran Biomart package. MiRNA sequences were obtained from miRBase database (v.22) (Kozomara et al., 2019). Enriched GO terms were obtained using two unranked lists of genes (target versus background) as described (Eden et al., 2009). Analysis results were visualized using Cran ggplot2 packages.
